# Application Scenarios for Artificial Intelligence in Nursing Care: Rapid Review

**DOI:** 10.2196/26522

**Published:** 2021-11-29

**Authors:** Kathrin Seibert, Dominik Domhoff, Dominik Bruch, Matthias Schulte-Althoff, Daniel Fürstenau, Felix Biessmann, Karin Wolf-Ostermann

**Affiliations:** 1 Institute of Public Health and Nursing Research High Profile Area Health Sciences University of Bremen Bremen Germany; 2 Auf- und Umbruch im Gesundheitswesen UG Bonn Germany; 3 School of Business and Economics, Department of Information Systems Freie Universität Berlin Einstein Center Digital Future Berlin Germany; 4 Department of Digitalization Copenhagen Business School Frederiksberg Denmark; 5 Institute of Medical Informatics Charité - Universitätsmedizin Berlin Berlin Germany; 6 Faculty VI - Informatics and Media Beuth University of Applied Sciences Einstein Center Digital Future Berlin Germany

**Keywords:** nursing care, artificial intelligence, machine learning, expert system, hybrid system

## Abstract

**Background:**

Artificial intelligence (AI) holds the promise of supporting nurses’ clinical decision-making in complex care situations or conducting tasks that are remote from direct patient interaction, such as documentation processes. There has been an increase in the research and development of AI applications for nursing care, but there is a persistent lack of an extensive overview covering the evidence base for promising application scenarios.

**Objective:**

This study synthesizes literature on application scenarios for AI in nursing care settings as well as highlights adjacent aspects in the ethical, legal, and social discourse surrounding the application of AI in nursing care.

**Methods:**

Following a rapid review design, PubMed, CINAHL, Association for Computing Machinery Digital Library, Institute of Electrical and Electronics Engineers Xplore, Digital Bibliography & Library Project, and Association for Information Systems Library, as well as the libraries of leading AI conferences, were searched in June 2020. Publications of original quantitative and qualitative research, systematic reviews, discussion papers, and essays on the ethical, legal, and social implications published in English were included. Eligible studies were analyzed on the basis of predetermined selection criteria.

**Results:**

The titles and abstracts of 7016 publications and 704 full texts were screened, and 292 publications were included. Hospitals were the most prominent study setting, followed by independent living at home; fewer application scenarios were identified for nursing homes or home care. Most studies used machine learning algorithms, whereas expert or hybrid systems were entailed in less than every 10th publication. The application context of focusing on image and signal processing with tracking, monitoring, or the classification of activity and health followed by care coordination and communication, as well as fall detection, was the main purpose of AI applications. Few studies have reported the effects of AI applications on clinical or organizational outcomes, lacking particularly in data gathered outside laboratory conditions. In addition to technological requirements, the reporting and inclusion of certain requirements capture more overarching topics, such as data privacy, safety, and technology acceptance. Ethical, legal, and social implications reflect the discourse on technology use in health care but have mostly not been discussed in meaningful and potentially encompassing detail.

**Conclusions:**

The results highlight the potential for the application of AI systems in different nursing care settings. Considering the lack of findings on the effectiveness and application of AI systems in real-world scenarios, future research should reflect on a more nursing care–specific perspective toward objectives, outcomes, and benefits. We identify that, crucially, an advancement in technological-societal discourse that surrounds the ethical and legal implications of AI applications in nursing care is a necessary next step. Further, we outline the need for greater participation among all of the stakeholders involved.

## Introduction

### Background

Despite a surge in funded research in the application of digital technologies toward a higher assurance of quality nursing care, in times of aging societies and skill shortages [1], the application of artificial intelligence (AI) in nursing practice is still scarce. In this context, AI can be defined as algorithms that enable learning from data sets to achieve intelligent, goal-oriented action.

Recent systematic and scoping reviews on the application of AI in nursing research (as well as in practice and emerging trends), covering original research published until October 2019, identified papers listed in medical and multidisciplinary databases. These included studies focused on machine learning (ML) methods, such as deep learning [2], or on health technologies that incorporate AI approaches themselves, such as robots or clinical decision support systems [3]. Various application scenarios have been identified, including clinical or organizational outcomes (eg, falls), admission decisions in emergency medicine, high-definition image recognition, as well as socially assistive robots or health care assistant chatbots [2,3]. In addition, recent years have seen an increase in research highlighting possibilities for the future development of AI in nursing care while underscoring the importance of collaborative, interdisciplinary research, and representative, robust data sets [2].

However, as of today, a universally accepted classification of AI subfields relevant to health, which could act as a vantage point for AI in nursing practice, is missing [4]. Prominent AI approaches include ML, expert, and hybrid systems. ML, as a method of data analysis guided by algorithms, identifies patterns in data and learns from them using different approaches [4]. This is utilized in medical diagnostics, for example [5]. Expert systems build on a knowledge base and a rule-based reasoning engine [4], which, in combination, mimic the reasoning of a human expert who would solve a complex problem by applying predefined if-then rules drawing on a specific knowledge base [6]. These systems can be found in tools that support clinical decision-making and case-based reasoning [7,8]. Hybrid systems combine different AI capabilities by integrating ML with expert systems [9-11]. AI applications aimed at determining the meaning of texts, such as clinical notes, can be found in the AI subfield of natural language processing (NLP) [4,12]. AI applications for automated planning and scheduling can be used to improve the efficiency of human procedures [4], such as generating nursing staff rosters or care-related scheduling decisions [13,14]. Applications that target image and signal processing use algorithms that typically include signal feature analysis and data classification to analyze images or data produced by movement or sound [4]. These can, for example, aim at activity and health monitoring, wound detection, or pressure injury and fall prediction or prevention [15-19].

### Opportunities and Challenges for AI in Nursing Care

Turning our attention specifically to nursing care settings, the primary opportunities for applying AI include application scenarios such as decision support in complex care situations [3,18,20,21]. AI also holds great promise for supporting nurses in tasks considered to take place remotely from direct patient interactions [3,21]. High expenditures of nurses’ working hours are frequently reported as being used for the documentation of care processes, with some care facilities reporting up to almost a third of daily working hours being expended for documentation processes [3]. This represents one of the many starting points from which to develop AI solutions to consistently improve nursing care processes and support nurses efficiently in their daily tasks. AI applications for the direct support of care-dependent persons and their informal caregivers are another starting point, as studies with AI approaches in different community and home care settings have shown [4-6]. This is of particular need, given that most long-term care recipients in Germany are being cared for in their own homes [22]. Until now, little knowledge on the practical relevance and applicability of AI systems with setting-specific requirements in nursing care, for example, when introduced in care processes involving persons with limited cognitive abilities, exists thus far.

Furthermore, the transformative effect of AI, resulting from its ability to change the intrinsic nature of health care delivery, is accompanied by ethical risks, namely, concerning the validity of evidence, the fairness of outcomes, and the traceability of harm caused by algorithmic activity [23]. Furthermore, although consensus on the potential of health technologies powered by AI to enhance nursing practice has been reported [3], the critical ideological and ethical nature of nursing practice still needs to be considered, and the role of decision-making, enhanced and burdened by an amplified understanding of opportunities granted by AI applications remains uncertain in the context of providing ethical and transparent nursing care [21]. To our knowledge, an extensive overview of the evidence base and status quo of research on AI for application in nursing practice, including evidence from medical and computer science databases, is missing. By identifying promising application scenarios for AI in nursing practice, such an overview contributes to the systematic enhancement of research and development for AI in nursing practice.

### Objectives

This rapid review aims to synthesize the evidence base of application scenarios for AI in nursing care settings, namely, ambulatory and stationary (long-term) care, acute hospital care, and nursing education. We also address prominent adjacent aspects within the ethical, legal, and social discourse concerning AI in nursing care by addressing the following review questions:

1. Which application scenarios for AI systems in nursing practice are reported, considering that different care settings are described in the literature?
2. What kinds of AI approaches have been researched, or are being discussed in the literature, and for which kinds of care settings?
3. What requirements or barriers have been reported for the application of AI in nursing practice?
4. Which ethical, legal, and social aspects—concerning AI and nursing—are discussed in the national and international literature?

Although our approach is broader than those of similar reviews that have focused exclusively on ML algorithms [24], it is also more broadly scoped in that it considers the ethical and regulatory context of the AI system deployed but does not focus exclusively on these aspects as other reviews have done [25].

## Methods

### Criteria for Considering Publications for This Review

We conducted a rapid review to identify and synthesize publications promptly [26]. A protocol describing the rationale and methods of this review was published in May 2020 [27]. This paper follows the guidelines outlined in the PRISMA (Preferred Reporting Items for Systematic Reviews and Meta-Analyses) Statement [28].

We included all designs of quantitative and qualitative original research, systematic reviews, and discussion papers or essays on ethical, legal, and social aspects that address the application of AI, specifically in:

1. The support of decision or work processes in direct nursing care or,
2. The organization of nursing care processes,
3. The support of knowledge and competencies in nurses’ (further) education or,
4. The support of persons in need of care (explicitly referred to as needing care) or,
5. Persons of all ages in need of support in their activities of daily living.

We included publications in the English language from 2005 onward, as we expect publications on AI to become quickly outdated and updated. Publications focusing on improving the functionality of medical diagnostic or therapeutic technologies, without clearly describing nurses as a relevant target group being affected by the application of the AI system or being directly involved in the application process, were excluded.

### Types of Participants, Settings, and AI Systems

Publications had to either designate at least 1 of the following groups of persons as main users or as benefactors of an AI application:

1. Nurses or nursing students
2. Care-dependent persons or their informal caregivers (either explicitly referred to as needing care or being referred to as needing, or benefiting from physical, cognitive, or mental support).

Publications using inconclusive terms, such as *the elderly* or *health care professionals*, without further information on target groups of users or benefactors, were also assessed for inclusion.

Care settings encompassed ambulatory and stationary long-term as well as acute outpatient and hospital care (including rehabilitation facilities) and nursing education settings. Studies assessing care in community settings or assessing the populations mentioned above, but in a laboratory setting, were also included.

As there is no conclusive definition of specific AI abilities or subfields that are relevant for health [4] or nursing care as of yet, all types of AI systems or approaches, ranging from clearly stated types (ML, expert system, hybrid system), to any type of approach combining ML and an expert system and algorithms, across to rather vague descriptions, such as *smart system* or *AI in health care*, were deemed as eligible.

### Search Methods for Identification of Studies

We searched the following databases in June 2020: PubMed, CINAHL (including Embase), Association for Computing Machinery Digital Library, Institute of Electrical and Electronics Engineers Xplore, Digital Bibliography & Library Project, computer science bibliography, and Association for Information Systems Library. In addition, we searched digital libraries of leading conferences identified through expert consensus within the study team. These conferences were specifically the Association for the Advancement of AI conference, the Association for Computational Linguistics Conference, the Conference on Computer Vision and Pattern Recognition, the International Conference on Machine Learning, the International Joint Conferences on AI Organization, the conference of the Association for Computing Machinery’s Special Interest Group on Knowledge Discovery and Data Mining, the Conference on Neural Information Processing Systems, the International Conference on Principles of Knowledge Representation and Reasoning, the Conference on Uncertainty in AI, the International Conference on Autonomous Agents and Multiagent Systems, and the European Conference on AI. The search strategy based on the block-building approach [29], combined terms for *nursing* and *artificial intelligence* and their respective synonyms. If applicable, we searched the titles, abstracts, and all fields of publication. In the first step, single terms for each block were searched. Second, all terms of a single block were combined using the Boolean operator *OR*. We initially deemed publications in German or English to be eligible. As no publications in the German language fulfilled the inclusion criteria, we focused only on the English. Finally, the results from the second step were combined for the 2 blocks using the Boolean operator AND. The hits were recorded for each step. To circumvent imprecisions between concepts described in the titles or abstracts, regarding the components of the search strategy, and to identify a large number of potentially eligible publications, we developed a preferably sensitive search strategy. Multimedia Appendix 1 contains the search strategy, search terms, and the number of hits for all databases and conference libraries.

### Data Collection and Analysis

#### Selection of Publications, Data Management, and Extraction

Two review authors independently screened all the titles and abstracts. Full texts were screened by a single person. Discrepancies were resolved through discussion or by referral to a third review author. Citations identified by the third search step described above were exported to an EndNote library after excluding duplicates. Titles and abstracts were screened using the web-based resource, Rayyan [30]. Full text screening was conducted in EndNote and documented using a spreadsheet program. Data extraction was conducted by a single reviewer and included the following data for all publications:

1. Author, year, country of origin
2. Setting
3. Target group of users or benefactors
4. Methods used or addressed
5. Purpose of the AI application

Furthermore, we extracted information on study design, type of data sets used, number of participants, outcomes assessed, results, and reported requirements or barriers for the application of AI for a subsample of studies that we considered as studies that incorporated real-world settings. For studies focusing on research of a more basic nature and describing laboratory scenarios or which used pre-existing data sets, either without transfer of results to real-world nursing scenarios or without evaluation of real-world outcomes, or focused on algorithm qualities or proof-of-concept studies, no information on results were extracted.

#### Assessment of Risk of Bias and Level of Evidence

As the rating of the effectiveness of AI applications in nursing care was not a primary research interest of this review, we did not assess the risk of bias of the results within the original research studies. To map the advancement of research regarding reliability, external validity, and generalization of results, a level of evidence (LOE) was assigned to each publication. We used established evidence-based nursing and evidence-based medicine hierarchies [31,32] and ranked LOEs from level I (highest evidence) to level VII (lowest evidence), as shown in Textbox 1. As we did not assess the risk of bias, the characteristic *well designed* in the LOE description is enclosed in brackets. Studies using a nonrandomized control group design, in which one group of participants did not receive an AI-supported intervention, or where a before-and-after design was implemented, were assigned to level III. Publications providing an overview without using a systematic review design were assigned to level VII. Publications not reporting results obtained by a specific research design were then labeled as *concept only* and were not assigned an LOE.

**Level of evidence rating categories.**

**Level of evidence**

- Level I
  Evidence from a systematic review or meta-analysis of all relevant randomized controlled trials (RCTs) or evidence-based clinical practice guidelines, based on systematic reviews of RCTs, or of 3 or more RCTs of good quality that have similar results
- Level II
  Evidence obtained from at least 1 (well-designed) RCT
- Level III
  Evidence obtained from (well-designed) controlled trials without randomization (eg, quasi-experimental)
- Level IV
  Evidence from (well-designed) case-control or cohort studies
- Level V
  Evidence from systematic reviews of descriptive and qualitative studies (metasynthesis)
- Level VI
  Evidence from a single descriptive or qualitative study
- Level VII
  Evidence from the opinion of authorities or reports of expert committees
- No applicable level
  Concept only

#### Analysis and Synthesis

Publications were grouped into basic research studies (category *basic or experimental*) or those incorporating real-world scenarios. The country of origin was coded into a country code, as defined in ISO 3166-1; it refers to the country in which the analyzed data were generated. We classified studies as either directly addressing nurses, care dependents, patients, or informal caregivers as being the main users or benefactors of the AI system. An AI system can solve complex problems that have been previously reserved for humans. This is done by breaking these problems into a number of simple prediction tasks [33]. We coded the types of AI systems and application contexts for each publication on the basis of the categories given in Wahl et al [4], which we expanded after determining the final sample of publications to be included. The category *Type of AI Approach* comprises the codes *machine learning* and *expert system*, as defined above. In addition, we also considered *hybrid systems*, defined as a combination of expert systems with ML [9-11]. Studies using deep learning approaches have been included in ML. AI systems can be defined as self-training structures of ML predictors, which automate and accelerate human tasks, and consist of *domain structure*, *data generation,* and a *general purpose prediction algorithm* [33]; information on the domain structure, which needs to be attributable to the nursing care context, was mandatory for inclusion. Studies lacking information on the data generation, as well as the prediction algorithm dimension, using rather generalized terms, were categorized as not specified in the category *Type of AI Approach*.

The application context category is also derived from Wahl et al [4] and comprises *automated planning and scheduling*, *image and signal processing,* and *NLP*. Both categories also entail codes for unclear and nonspecific information or restricted applicability. Originating from the data extracted for the purpose of the AI application, we inductively developed codes for the *setting* category and 22 codes for the *purpose* category that summarize the domain of health or nursing activity affected by the AI system (eg, nurse rostering and scheduling, tracking or monitoring of activity and health tracking, falls or quality of life, and well-being of caregivers). In addition, we inductively derived 7 codes for a more generalized *application scenario* (support of direct nursing care, support of the care organization, support of independent living care-dependent people, health of the caretaker, formal and informal education, risk estimation and prevention, etc). Systematic reviews and other types of publications were coded as described above if possible, or rated as not applicable for some categories.

Study characteristics and target groups of users or benefactors are descriptively summarized and displayed in tables and figures. To answer the first and second research questions, we descriptively summarized the categories *purpose*, *application scenario, type of AI approach,* and *application context* in relation to the *setting* category. The results will be summarized, as well as differentiated, for studies considered to be of a more basic nature, such as laboratory experiments or proof-of-concept papers (category *basic or experimental*) and real-world scenario studies (category *real-world setting*) and displayed in tables or figures. To answer the third research question, we narratively summarized the requirements and barriers reported in real-world scenario studies, as well as systematic reviews or publications focusing on the ethical, legal, and social implications (ELSIs) of AI in nursing care. The latter also provides the basis for answering the fourth research question in the form of narrative synthesis.

## Results

### Included Publications

#### Overview

**Figure 1:**
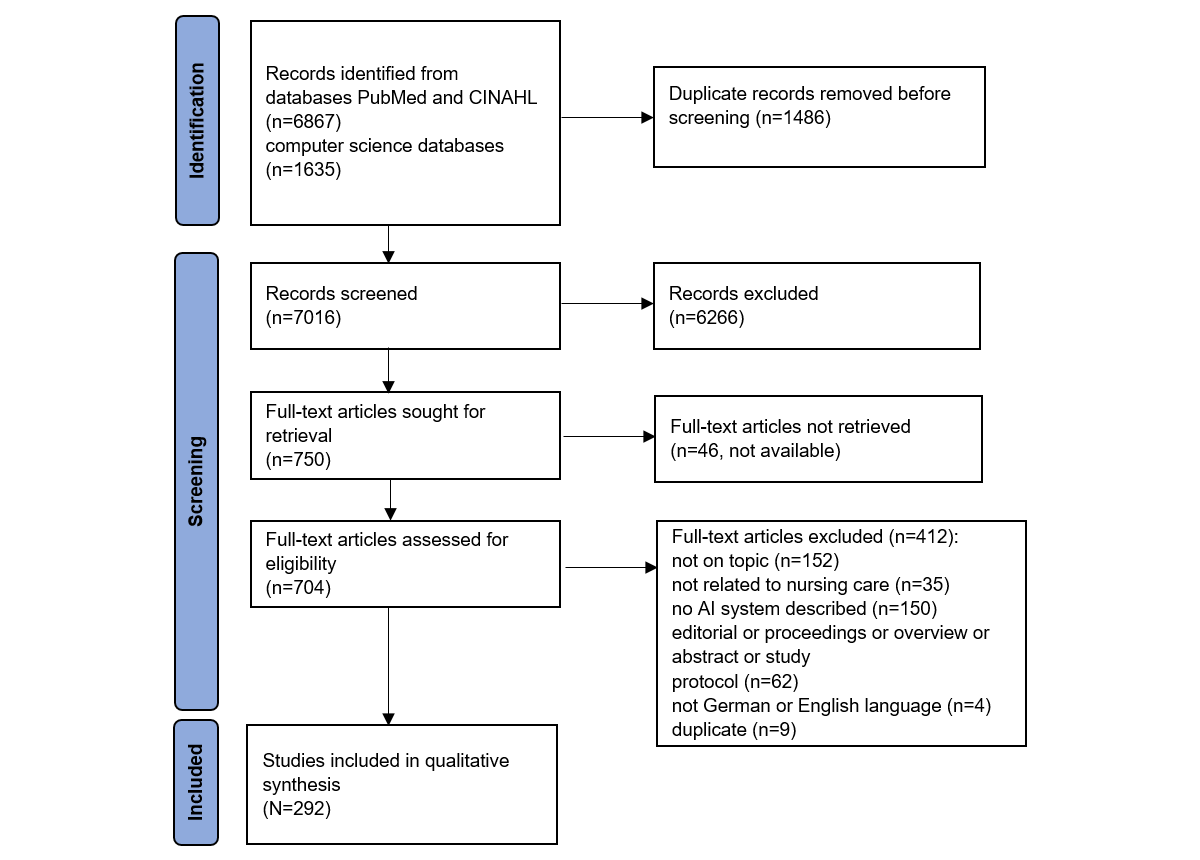
PRISMA (Preferred Reporting Items for Systematic Reviews and Meta-Analyses) [34] flowchart of the publication screening process and study selection. AI: artificial intelligence.

Searches performed in databases for nursing and health sciences yielded 6867 matches. Databases containing publications from computer science publications added an additional 1635 matches. The handling of the included publications and the numbers of included and excluded records are depicted in Figure 1. In the first step, we eliminated duplicate records (n=1486), resulting in 7016 publications proceeding into the screening of their titles and abstracts, which led to the further exclusion of 6266 publications. For the remaining 704 available publications, the full texts were screened, and a further 412 publications were excluded in this step, leaving 292 publications to be incorporated in this review (Multimedia Appendix 2 [7-13,15-19,21,34-310] describes an overview of all 292 references and the selected characteristics for these included publications).

#### Characteristics of Included Studies

##### Publication Date, Country, and Publication Language

Of the 292 studies, 155 (53.1%) were published between 2016 and June 2020, with the remaining 137 (46.9%) originating from 2005 to 2015. The included studies used data generated in 39 countries, which in most cases corresponded with the country affiliation of the first author. The 10 countries with the most publications were the United States (n=72), Japan (n=45), Canada (n=23), China (n=16), Taiwan (n=15), the United Kingdom (n=11), Australia (n=10), India (n=9), Spain (n=9), South Korea (n=8), and Germany (n=8). All studies were published in English.

##### Research Setting

We classified 83.2% (243/292) of studies as basic or experimental and 11.6% (34/292) as studies in real-world settings. In addition, 8 scoping or systematic reviews, 6 publications on ethical, legal, and social aspects, and 1 survey on AI in nursing practice were included.

#### Level of Evidence

The LOE assigned most often was level VI (evidence from a single descriptive or qualitative study), which applied to 77.1% (225/292) of studies. An evidence level of III (evidence obtained from well-designed controlled trials without randomization) or higher was assigned to 2.1% (6/292) of studies. Table 1 shows the number of studies assigned to each level of the evidence category.

**Table 1:** Numbers of publications by level of evidence and research setting (N=292).

Level of evidence	Basic or experimental (n=243), n (%)	Real-world setting (n=34), n (%)	Other (n=15), n (%)	Total (N=292), n (%)
Level I	0 (0)	0 (0)	1 (6.7)	1 (0.3)
Level II	0 (0)	1 (2.9)	0 (0)	1 (0.3)
Level III	0 (0)	4 (11.8)	0 (0)	4 (1.4)
Level IV	7 (2.9)	7 (20.6)	0 (0)	14 (4.8)
Level V	0 (0)	0 (0)	7 (46.7)	7 (2.4)
Level VI	199 (81.9)	21 (61.8)	5 (33.3)	225 (77.1)
Level VII	4 (1.6)	0 (0)	2 (13.3)	6 (2.1)
No applicable level	33 (13.6)	1 (2.9)	0 (0)	34 (11.6)

#### Beneficiaries and Setting

We found that 46.2% (135/292) of publications specifically addressed care-dependent persons as the target group of the proposed or examined AI solutions, 39.4% (115/292) targeting nurses, and 9.6% (28/292) stating informal caregivers as the target group. Two or more of the aforementioned groups were addressed in 49 studies, and all of them in 6 publications. In addition, 23.3% (68/292) of the publications did not state either of the 3 groups as their primary target group. These studies frequently proposed AI approaches with nurses targeted as potential beneficiaries among other health care professionals.

Hospitals are the most prominent research setting, followed by independent living at home, with nursing homes, ambulatory long-term care, and outpatient health care being less frequently addressed (Table 2). Other settings, including the community, rehabilitation, daycare, and education facilities have been the subject of only a few studies. Multiple settings were the subject of 10.6% (31/292) of the publications, and 11.3% (33/292) did not state any setting. Studies employing a real-world setting also predominantly focused on hospitals. Other settings were only referred to infrequently.

**Table 2:** Numbers of publications by application and research setting (N=292).

Setting	Basic or experimental (n=243), n (%)	Real-world setting (n=34), n (%)	Other (n=15), n (%)	Total (N=292), n (%)
Hospital	70 (28.8)	13 (38.2)	4 (26.7)	86 (29.8)
Independent living	64 (26.3)	0 (0)	2 (13.3)	66 (22.6)
Nursing home	21 (8.6)	12 (35.3)	0 (0)	32 (11.3)
Ambulatory long-term care	11 (4.1)	6 (17.6)	0 (0)	18 (5.8)
Outpatient health care	10 (4.1)	0 (0)	0 (0)	10 (3.4)
Community	6 (2.5)	1 (2.9)	1 (6.7)	9 (2.7)
Rehabilitation	2 (0.8)	0 (0)	0 (0)	2 (0.7)
Daycare	0 (0)	1 (2.9)	0 (0)	1 (0.3)
Education facility	0 (0)	1 (2.9)	0 (0)	1 (0.3)
Multiple	26 (10.7)	0 (0)	5 (33.3)	31 (10.6)
N/A^a^	3 (1.2)	0 (0)	0 (0)	3 (1)
Not stated	30 (12.3)	0 (0)	3 (20)	33 (11.3)

^a^N/A: not applicable.

### Type and Subtype of AI Approaches

Considering the type of AI approach, we found the vast majority (228/292, 78.1%) of studies employing ML approaches. Rule-based expert systems were used in 11.6% (34/292) of the publications, whereas hybrid systems were used in only 3 studies. For the remainder of the publications, the specific AI approach used was either not identifiable or this attribute was not applicable, mostly because of publications not employing specific AI approaches in basic or applied research. Studies incorporating real-world settings made use of ML approaches comparably in 71% (24/34) of cases, whereas expert systems were only covered in 2 studies and hybrid systems not at all (Table 3).

Most AI approaches have been described as solutions for image and signal processing (178/292, 60.9%), that is, the processing of large amounts of signals, such as audio and video data, for feature analysis and data classification [4]. AI approaches have been used for automated planning and scheduling. This category entails approaches used to organize and prioritize activities and that “can be used to improve the efficiency of human procedures” [4]. Studies have focused less often on the processing of human language (NLP). Image and signal processing were also performed in most studies conducted in a real-world setting with automated planning and scheduling, and research on NLP has been less frequently reported (Table 4).

**Table 3:** Numbers of publications by type of artificial intelligence (AI) system and research setting (N=292).

Type of AI system	Basic or experimental (n=243), n (%)	Real-world setting (n=34), n (%)	Other (n=15), n (%)	Total (n=292), n (%)
Machine learning	197 (81.1)	24 (70.6)	7 (46.7)	228 (78.1)
Expert system	29 (11.9)	4 (11.8)	1 (6.7)	34 (11.6)
Not specified	12 (4.9)	6 (17.6)	7 (46.7)	25 (8.6)
Hybrid system	5 (2.1)	0 (0)	0 (0)	5 (1.7)

**Table 4:** Numbers of publications by subfield of artificial intelligence (AI) and research setting (N=292).

Subfield of AI	Basic or experimental (n=243), n (%)	Real-world setting (n=34), n (%)	Other (n=15), n (%)	Total (N=292), n (%)
Image and signal processing	155 (63.8)	19 (55.9)	4 (26.7)	178 (61)
Automated planning and scheduling	59 (24.3)	14 (41.2)	1 (6.7)	74 (25.3)
Natural language processing	26 (10.7)	1 (2.9)	0 (0)	27 (9.2)
Not specified	3 (1.2)	0 (0)	10 (66.7)	13 (4.5)

### Purpose of AI Application

The areas of support for nursing care targeted by the AI approaches are shown in Table 5. With regard to the intended effects from the described AI approaches, 47.6% (139/292) of the studies focused on the support of the direct, immediate process of care. The support of the organization of care services and the support of care-dependent people themselves, as well as risk estimation and prevention, are further prominent purposes. Risk estimation and prevention potentially pose a cross-sectional topic, where the type of support manifests at multiple levels. The health of the caregiver and education were addressed in only a few cases. For studies in real-world settings, risk estimation or prevention, support of direct care, and support of care organization were each a focus of 29% (10/34) of the studies, whereas the support of care-dependent people and education did not play a prominent role, and no research conducted in a real-world setting focused on the health of caregivers.

**Table 5:** Numbers of publications by area of support and research setting (N=292).

Area of support	Basic or experimental (n=243), n (%)	Real-world setting (n=34), n (%)	Other (n=15), n (%)	Total (N=292), n (%)
Support of direct care	125 (51.4)	10 (29.4)	4 (26.7)	139 (47.6)
Support of care organization	42 (17.3)	10 (29.4)	0 (0)	52 (17.8)
Risk estimation or prevention	32 (13.2)	10 (29.4)	0 (0)	42 (14.4)
Support of care-dependent people	33 (13.6)	3 (8.8)	3 (20)	39 (13.4)
N/A^a^	5 (2.1)	0 (0)	6 (40)	11 (3.8)
Health of caretaker	4 (1.6)	0 (0)	0 (0)	4 (1.4)
Various	1 (0.4)	0 (0)	2 (13.3)	3 (1)
Education	1 (0.4)	1 (2.9)	0	2 (0.7)

^a^N/A: not applicable.

A more detailed summary of the purpose of the AI approaches is presented in Table 6. The most prominent purpose was activity and health tracking (monitoring or classification) in 30.1% (88/292) studies. Care coordination and communication are frequent topics, which, among others, include AI approaches classifying information in nursing documentation, supporting decision-making, and yielding information for coordination and continuity of care. Fall detection, fall prevention, and fall risk classification are also frequently mentioned purposes for topics in AI. In contrast to falls, other mobility-related aspects were of lesser interest and were mentioned in only a few studies. Further purposes with a high degree of specificity are the recognition, classification, reduction of alarms, and risk prediction and classification of pressure ulcers. Addressing nurse rostering or scheduling problems was the purpose of an AI solution in 4.1% (12/292) studies.

**Table 6:** Frequencies of stated purposes (monitoring, tracking, classification, prediction, and support) of artificial intelligence solutions (N=292).

Purpose	Frequency, n (%)
Activity and health	88 (30.1)
Care coordination and communication	53 (18.2)
Falls	36 (12.3)
Nursing assessment or care needs assessment	21 (7.2)
Alarms	14 (4.8)
Nurse rostering or scheduling	12 (4.1)
Pressure ulcers	11 (3.8)
Social integration and participation	10 (3.4)
Parenteral or enteral nutrition and fluid intake	7 (2.4)
Quality of life and well-being of caregivers	6 (2.1)
Mobility, other	5 (1.7)
Speech	5 (1.7)
Distribution of medication	3 (1)
Wound management (excluding pressure ulcers)	3 (1)
Bladder control	2 (0.7)
Infection control	2 (0.7)
Respiratory care or weaning	2 (0.7)
Clinical education	1 (0.3)
COPD^a^ care	1 (0.3)
Digestion management	1 (0.3)
Pain assessment or management	1 (0.3)
N/A^b^	8 (2.7)

^a^COPD: chronic obstructive pulmonary disease.

^b^N/A: not applicable.

### Subsample of Studies in Real-world Settings

#### Overview

We classified 34 publications as studies that employed real-world settings. Multimedia Appendix 3 [13,18,19,34-58,63,69,82,95,100,310,312] summarizes the characteristics of these publications. The data used originated from the United States in 12 studies, from Canada in 5 studies, and from Spain in 3 studies. Australia, Germany, and Japan contributed 2 studies each to the subsample, and the remaining single studies used data from Brazil, Finland, Greece, Hong Kong, Ireland, Italy, and Singapore. A Saudi Arabian survey on health care employees’ perceptions of the use of AI applications that involved 121 nurses as participants [34] was also included in the subsample. In the 27 studies that reported on the number of participants, sample sizes ranged from small samples including <10 people [35,36] to large data sets holding information from >200,000 individuals [13,37]. Of the 31 studies reporting more details on participant characteristics, data from patients in hospitals were analyzed in 13 studies, of which 5 focused on pediatric or adult intensive care unit (ICU) patients [13,38-40,312]. Residents of long-term care institutions were included in 12 studies that sometimes also included caregivers and other health professionals [35,41-44]. People with dementia or cognitive impairment were included in 3 studies [36,42,45]. Home care clients or community-dwelling elderly were included in 6 studies [46-51,313], 3 studies specifically focused on nurses or nursing students [34,52,53], and 1 study targeted elderly people at a daycare facility [54]. The more or less detailed reporting of heterogeneous study designs included experimental designs, field experiments [54], real-life use-cases [55], case studies with a single subject design [36], cross-sectional and longitudinal observational designs as well as comparative designs, and an economic evaluation nested within a cluster-randomized controlled trial [18,45], and different mixed methods designs (such as the studies by Amato et al [35], Ala-Kitula et al [46], and Alwan et al [47]). However, some studies did not state a specific design. In that case, they were classified according to the nature of the reported results (such as observational data).

#### Reported Effects Referring to Clinical or Organizational Outcomes

Of the studies in the subsample reporting results in varying degrees of detail, 22 studies reported effects in terms of algorithm eligibility or technological functionality. For example, Chen et al [42] developed a detector for elopement behavior in dementia care units on the basis of a hidden Markov model and concluded that the system may reduce the risk of actual unwitnessed elopement, thus preventing negative consequences of elopement, but did not report on the longitudinal implementation of the detector or changes in elopement rates or nursing work processes. In contrast, no additional benefit by applying AI technology for a use-case aimed at gathering personal health data from home monitoring sensors, activity trackers, national electronic health records, and previous home care reports to evaluate the home care need and its availability in real time was reported by Ala-Kitula et al [46]. Results for outcomes that we considered to be of a more clinical or organizational nature are reported in 12 studies and highlight the real-world effect of the contribution of AI approaches to nursing care. Of those studies, 7 were conducted in long-term care facilities, 3 in the hospital setting, and 1 study in a daycare facility or an educational setting. Outcomes mainly target some form of physical activity, movement, or response but also, among others, length of stay (LOS), mortality, pressure ulcers, and handwashing skills.

Bajo et al [52] tested the ability of a multiagent architecture for geriatric residences to provide efficient working schedules by utilizing ML in a case-based reasoning approach. In a sample of 10 nurses, the time spent on supervision and control tasks as well as the time spent on attending to false alarms was reduced, whereas the time for direct patient care increased during the observation period of 6 months [52]. Another multiagent system to enhance assistance and health care for patients with dementia living in geriatric residences with reasoning and planning mechanisms was introduced by Tapia et al [56]. The application of the system led to a reduction in the average number of minutes spent by nurses on the monitoring of residents from more than 150 daily minutes (before implementation) to approximately 90 daily minutes (after implementation). In addition, the number of nurses working simultaneously before and after the implementation of the system reduced, and unauthorized access to restricted zones of the residence was detected almost twice as often after implementation [56]. Tang et al [43] developed a cloud-based nursing care planning system and applied case-based reasoning and text mining to facilitate decision-making of nurses responsible for admissions in a nursing home. In an observational study lasting 6 months, the efficiency of nursing care plan formulation and the response time in handling new applications increased, whereas the number of revisions of the care plan decreased. The time waiting for supporting documents reduced from 24 hours before the implementation of the system to 6.75 hours after the implementation, and the time spent searching for health care information reduced from 90 to 20 minutes, whereas the adoption of traditional health care services increased, and the residents’ complaint rate decreased [43]. Xiong et al [44] examined the use of a scalable AI-enabled camera monitoring system to detect and record falls and notify nurses to perform video review of the incident immediately after each fall of residents with dementia in residential care facilities. Compared with a control group of residents who also experienced falls but were not monitored by the system, relative reductions of emergency medical team visits and emergency department visits of 75% (emergency medical team visits: *P*=.001) and 80% (emergency department visits: *P*=.003), respectively, were observed. [44]. Cho et al [18] developed a decision support intervention using a Bayesian network model to predict hospital-acquired pressure ulcers and assessed its effectiveness on the prevalence of ulcers and ICU LOS as well as on the user adoption rate and attitudes in a controlled trial. Patients in the intervention group had a decreased risk of developing hospital-acquired pressure ulcers (odds ratio 0.1; *P=*<.001) and a shorter ICU LOS (odds ratio 0.67; *P=*<.001), whereas nurses expressed favorable attitudes toward using the system [18]. Evans et al [57] developed an expert system to identify early signs of physiological deterioration in hospital patients and conducted a longitudinal evaluation of its impact on ICU transfer rates, medical emergency team calls, and mortality. During the 1-year intervention, ICU transfers and medical emergency team calls increased significantly and mortality decreased significantly when compared with the preintervention year for patients on a medical and oncology floor, whereas no significant increase was found for patients on a non-ICU surgical trauma floor that were younger and had fewer comorbidities than patients on the medical and oncology floor [57]. Yamamoto et al [53] used ML to evaluate handwashing skills in nursing students and reported that, when comparing students 3 months after their last training and beginners in handwashing, handwashing skills were almost identical, indicating the need to update practice handwashing beyond initial training. Viswanathan et al [36] observed residents of a long-term care facility with mild-to-moderate cognitive impairment while using an intelligent wheelchair and reported lower mean frontal collision with objects compared with using the wheelchair without the avoidance module preventing wheelchair motion toward nearby obstacles being activated while observing large differences in the users’ collision avoidance ability. Zampieri et al [38] used ML to assess whether ICU staffing features are associated with improved hospital mortality, ICU LOS, and duration of mechanical ventilation and identified 3 clusters with, for example, the extent of nurse autonomy as a distinguishing feature and the cluster with the highest nurse autonomy exhibited the best outcomes, with lower adjusted hospital mortality and shorter ICU LOS for patients surviving to ICU discharge and shorter durations of mechanical ventilation. For studies including robotic systems, Mervin et al [45] found marginally greater values in terms of incremental cost per Cohen-Mansfield Agitation Inventory Short Form point averted from a provider’s perspective for a plush-toy alternative than an emotional robot seal, but deemed the robot seal a cost-effective psychosocial treatment option for reducing agitation in people with dementia in long-term care as well, as costs are much lower than those estimated for psychosocial group activities and sensory interventions. Matsuyama et al [54] introduced a communication robot to activate and improve group communication and observed an increase in participation in terms of the frequency of smiles and answers in response to the robot system. Carros et al [41] explored, among other outcomes, stakeholders’ attitudes, social and organizational practices, and expectations of the Pepper robot in individual and group-based performances and revealed the potential for humanoid robots working in nursing homes, as well as the necessity of a person in control of the robot acting as a moderator.

### Requirements and Barriers

Requirements or challenges and barriers for the application of AI in nursing care outside of technological infrastructure or reliability, precision, and validation of data have been reported in 6 studies, including real-world scenarios [18,35,37,41,58,314] and 4 reviews [59-62]. Requirements included compliance with the EU General Data Protection Regulation and preferences of target users concerning usability and complexity, and also requirements stemming from implementation in specific care settings [35,60]. Furthermore, providers need to concern themselves with their capacity, ability, and willingness to generate data inputs required to achieve high accuracy, in contrast to the clinical burden of false positive or negative results [58]. In addition, the quality of administrative databases should not be affected by AI implementation in the care context [58]. Inclusion of caregivers, user engagement, and commitment to further the participation of older adults in the development and testing of AI systems as well as successful implementation are required [18,41]. Reported challenges and barriers target accuracy of recognition, integration with sensor networks, privacy, security, human–machine interaction, and cognition impairment of users, acceptance, and costs [59,61]. The physical appearance and programmed behavior of hardware hosting AI systems when presented in a humanoid form may seem confusing, unpredictable, or frightening and limit the interpretability of the system’s action for nursing home residents [41]. In addition, the appearance of sensors when they are nondisposable and bulky may pose a burden to caregivers [63]. Underreporting of relevant events and scarce public availability of databases holding sufficient data and information to compare one’s results, as well as limitations to data sets due to regional data protection laws, constitute barriers to the accuracy of algorithms and the external validity of results [37,62].

### Ethical, Legal, and Social Aspects

We identified 6 publications [7,21,64-67], specifically focusing on the ethical, legal, and social aspects of AI in nursing care. In addition, 7 publications [35,41,57,59,60,68,69], which were either reviews or studies including real-world settings, addressed selected ELSI aspects when discussing results or limitations of their work. Recurring aspects were *consent* (of care dependents or nurses), *data privacy and safety*, *acceptance* and *implications for work processes and workforce*, such as lack of human interaction and communication skills or the fear of replacement of nurses by technology and the implications arising from choosing humanoid designs for hardware hosting intelligent technologies [41,67]. Peirce et al [21] focused on relevant ethical, legal, and social aspects of nursing as a profession and highlighted implications arising from the type of data used, such as possible sampling bias, correlational false positives, and hidden discrimination, as well as the values and interests of companies building huge data sets that should be kept in mind. They pointed out the importance of nurses’ understanding of the underlying motivations and goals for creating algorithms as well as the learning mechanisms and potential to mediate, as AI-generated knowledge should not be regarded as universally valid and the potential of algorithms to limit nursing actions and cause the loss of human dignity should be regarded of utmost importance within the discourse on AI in nursing [21].

## Discussion

### Principal Findings

The results of this rapid review explicate the application scenarios for AI systems in nursing care. Hospitals, followed by independent living at home, were the most frequently investigated settings, whereas nursing homes and ambulatory long-term care were less often examined. The vast majority of studies applied ML, whereas expert and hybrid systems were used only in about every 10th publication. This implies that the current instance of AI is mainly ML driven. For instance, Taddy [33] described the evolution of ML toward status as a general purpose technology and, consequently, as the main driver of the current rise in AI. More than half of the publications focused on image and signal processing and one-third on automated planning and scheduling, whereas NLP appeared in less than 1 in 10 publications.

In the context of direct nursing care, AI is used to organize care processes and support care-dependent people or family caregivers through tracking, monitoring, or classifying activities and health data. This was followed by applications to support care coordination or communication, as well as nurse rostering and scheduling. Detecting, classifying, and preventing falls, as well as recognizing, classifying, and reducing alarms, and predicting and classifying pressure ulcers were further purposes of introducing AI to nursing care. Only a few publications went beyond proof-of-concept studies or laboratory experiments and applied AI in real-world scenarios. Few studies have assessed the effects of AI on clinical and organizational outcomes. In addition to technical or computational requirements, further requirements concerning the specific context of nursing care are scarce and mainly tackle overarching topics, such as data privacy, safety, and acceptance. The same holds for ELSIs, which, for instance, have not been reflected or discussed in most studies using real-world scenarios.

Most studies describe AI applications in hospital settings, particularly ICUs. This may be attributed to the availability of such data. Besides electronic medical, nursing, or health record data, real-time sensor data on vital parameters are more frequently available from ICUs than from regular wards, facilitating a multidimensional approach, which is, for instance, being used to classify risks or identify care needs. This finding is in line with a previously reported increase in the publication rate of studies using ML to analyze routinely collected ICU data [70]. The availability, quality, and quantity of data might also be limited due to differences in digitalization activities in specific care settings, as well as the sufficient inclusion of study participants and duration observation periods to generate large data sets from sensor data. Furthermore, the heterogeneity of different data sets complicates the use of data for AI development.

Considering the development of digital technologies in general, nurses themselves have reported that they feel that regular hospital wards or long-term care settings are being considered too little [315], which is consistent with our results. It should be noted that some of the included application scenarios cannot be attributed unambiguously to the nursing domain. Although an impact on nurses and patient care is evident when trying to reduce false alarm rates in monitoring [71,316] or to improve mechanical ventilation and sedative dosing processes [72], there is a blurred line between AI systems to support medical diagnostics and therapy, and AI systems to support nursing care. Considering the variety of applications for tracking, monitoring, and classifying health and activity, nurse rostering and scheduling, or detecting falls or fall risks, it is remarkable that so few studies went beyond testing the efficacy of AI approaches. This points to a gap in the existing evidence regarding the effectiveness of AI in real-world scenarios. This is also reflected in the results of the LOE ranking, with only a few studies using designs that could test effects on real-world nursing or on patient outcomes.

In addition, the explicit operationalization of nursing tasks or care processes, and desired clinical, psychosocial, or organizational outcomes, has not been addressed in most of the included studies. On the one hand, this might point to undiscovered possibilities for AI support in nursing care. On the other hand, it might limit the perceived benefit of AI in nursing care and, subsequently, the participation of providers, nurses, care dependents, and family caregivers in developing (as well as sustainably integrating) AI in care processes and everyday activities. Integration of AI in nursing care might also be limited by the lack of a sound description of outcomes, benefits, or values, which will influence the adoption or nonadoption of technologies in nursing care [315,317]. Our review points to a gap in published research on possible application scenarios for AI in nursing care on the one hand, and on the availability of evaluation results regarding already implemented AI systems on the other. This raises the presumption that such evaluations have so far been of less interest. This is particularly troublesome because some studies suggest little or no extra benefit of using AI when compared with alternative or existing solutions [45]. Concerning nurses’ need for technologies providing enhanced technological support of direct nursing care tasks to reduce physical burdens and mental stressors [315], there seems to be room for research on AI-sensitive outcomes in nursing care.

Concerning the requirements and barriers for AI in nursing care, we expected to find topics such as data quality and access, as well as factors associated with measuring primary data and obtaining and sharing routine data, more frequently reflected in the included publications. However, only a few studies have addressed these concerns. Most requirements and barriers mirrored topics that are not only relevant for AI systems, but also for digital technologies in nursing and health care in general, such as data privacy, safety, and user acceptance [318]. On the one hand, this could indicate that there are few nursing-specific requirements or barriers to consider. However, this seems unlikely given the heterogeneous origins of the included studies, which have been conducted in different societal and health systems, including, for instance, different regulations on data protection or storage. In contrast, the lack of data and access-related factors could be attributed to the fact that the descriptive or conceptual nature of most studies led to authors addressing requirements or barriers less frequently.

The ELSIs discussed in the included publications addressed prominent topics in the discourse on the use of technology in health care, such as data privacy and protection, consent, acceptance, and implications for communication [20,25]. These aspects were not addressed in most studies in the subsample of studies, including real-world settings. Only one publication focused specifically on the ethical implications arising from the knowledge generated by AI systems in the context of nursing care. Other ethical principles incorporated in existing AI guidelines, such as trust, sustainability, justice, and fairness [25], are covered superficially if at all. Even though the limited uptake of ELSI aspects in published research might be biased by the fact that the remaining publications were not screened for ELSI aspects, there seems to be room for researchers to incorporate the discussion of ELSI aspects in their work, contributing to building trustworthy and trusted AI solutions [319]. Furthermore, publications describing stakeholder processes, surveys, interviews, or focus groups involving care dependents or nurses and accessing their perspective on AI, were underrepresented in our sample. This indicates that there is room for implementing and facilitating the concept of participatory development and testing, which contribute to the demand orientation and acceptance of AI systems.

### Strengths and Limitations

A major strength of this review is the sensitive design of the search strategy, which led to the inclusion of a large sample of study designs and publication types, giving an extensive overview of published works on applications of AI systems in nursing care, as well as considering publications from medical- and informatics-databases and conference archives, which to our knowledge is the first of its kind.

The decision to focus on published works limits the results, as our strategy did not include AI systems already in use for which scientific empirical evidence has not been published and which might be directly introduced to clinical practice by developers. Another notable limitation is our decision to use a rather broad definition of nursing care, care recipients, and care settings, which also included independent living of elderly people. As some of the included publications did not define nursing care as the primary application context, and often included nurses or nursing care facilities as possible users among others, publications dealing with borderline examples of AI application scenarios that might or might not be attributed to the domain of nursing depending on the originating context of the publication, such as medical diagnostics, were not included.

As we used the criterion of conducting field experiments or using real-world data to group studies into basic, experimental, or real-world scenarios, studies in the basic or experimental subsample tested applications that may be considered extending well beyond basic research topics when using other criteria to classify studies. We chose this classification primarily to show the extent of the AI solutions applied in real-world practice. It also needs to be noted that we chose, primarily, absolute and relative numbers of studies and categories to map the existing literature; however, this provides an overview of prominent research topics and points to gaps in the existing literature; however, this does not allow for an assessment of the sophistication of research on AI approaches done in the context of nursing care. Although an assessment of scientific impact, quality of content, originality, and clarity, as it had been done in the field of sensors, signals, and imaging informatics to identify research works that exemplify recent developments [320] was not part of this review, a number of papers have demonstrated useful techniques to improve the generalizability, interpretability, and reproducibility of increasingly sophisticated models. As we only included publications in English, language bias must be noted. The same holds for the decision to limit the publication range to 2005, and to the selection of the searched databases and conferences, which restrict the sensitivity of the search strategy. However, the increase in publications during the last 5 years indicates that our search managed to cover a relevant period of research and development in AI for nursing care.

### Comparison With Previous Work

To our knowledge, this is the first review of its kind to systematize a broad literature base on AI in nursing care, and previous relevant work on this topic is scarce. A direct comparison with the application of AI approaches in other related domains, such as medicine or global health, is difficult, owing to, among other things, reported heterogeneity in AI reporting and the lack of a standardized benchmark [321], which was also present in our sample of studies. Even though disciplines such as biomedicine seem to be more active than nursing in terms of publications targeting AI applications [322], overlapping areas and specific aspects to study such as ethical aspects as well as the impact of digital health interventions and the changes and requirements for the professional role can be noted, for example, in the medicine domain [323]. In relation to the nursing domain, Kikuchi [2] reviewed studies on AI technologies in nursing research that focused on clinical outcomes, such as fall prediction, surgery-related injury, nausea, depression, and survival of patients, as well as on managerial themes addressing bed allocation, decision support, communication risks, nurse burnout, nurses’ intention to quit, nursing diagnostics, and knowledge acquisition for nurses. Without including publications outside of medical databases, similar to most studies included in our review, the results indicate a focus on the performance capability of AI algorithms compared with standard statistical methods, underlining the assumption of a lack of evaluation studies on existing AI solutions. Buchanan et al [3] conducted a scoping review on emerging trends in AI-powered health technologies and their implications for domains of nursing, such as administration, clinical practice, policy, and research. In contrast to our current review, specific types of AI approaches were not reported. The described emerging trends are parallel to the application scenarios in this review and entail predictive analytics, clinical decision support systems, smart homes, and health care assistant chatbots, but lay a focus on robot applications [3], which were only sparsely included in this review, as most studies on robots included in the screening process did not meet the inclusion criteria for full text review. The potential of AI to enhance nursing practice, as well as the need for nurses to take on the shared responsibility to influence and take part in the way AI is integrated into the health system, are highlighted by the results [3], contributing to the importance of engaging in the discussion from the perspective of clinical practice and nursing science. Congruent with Shillan et al [70], who reviewed the use of ML approaches in the ICU context, a lack of methodological reporting guidelines for AI approaches using health care data or being conducted in a health care or nursing setting impedes the identification of relevant studies, as well as with the evaluation and rating of real-world relevance, confidence in reported findings, and translation into clinical practice.

### Conclusions

#### Implications for Practice

The aim of this paper was, first, to describe which application scenarios for AI systems in nursing practice were considered in the existing literature. Second, we aimed to show the kinds of AI approaches that have been researched, or are being discussed in the literature and the kinds of care settings involved. Third, we investigated the requirements for the application of AI in nursing practice. Finally, we investigated the ethical, legal, and social aspects of AI and nursing, which are being discussed in the national and international literature.

The results show a broad spectrum of possible application scenarios and facilitate the participation and piloting of existing AI solutions. Because empirical evidence generated in real-world settings is limited, more knowledge on the benefits and advantages of AI approaches, compared with alternative solutions or usual care, is of great need. To date, little is known about the perspectives and experiences of nurses, care dependents, and informal caregivers, who should seek to take an active role in the scientific and societal discourse on AI in nursing care. By educating themselves on the potential harms and benefits of AI applications, they can empower themselves to influence how AI systems will be integrated into their daily lives and practices. Care facilities can contribute to AI development and research by promoting digitalization and ensuring data quality and availability, as successful research and application of AI depends on access, quality, and quantity of data.

#### Implications for Research

Our results provide an overview of application scenarios for which empirical evidence on algorithm accuracy has been published within the last 15 years. Considering the lack of findings on the effectiveness and application of AI approaches in real-world scenarios, future research should reflect on a more nursing care-specific perspective in their objectives, outcomes, and potential benefits. Aside from clinical, organizational, and managerial outcomes, which can be operationalized from care facility perspectives, our results provide new insights for research activities. Furthermore, discourse on the ethical, legal, and societal implications of AI applications in nursing care, as well as on the participation of stakeholders, needs to be advanced.

#### Implications for Policy Makers

Half of the publications in our sample have been published during the last 4 years, indicating an increase in research and funding, specifically concerning the application of AI systems in nursing care, with a large uptick of published experimental research. Policy makers and funding bodies might want to reflect on particular priorities for their future grants and programs, against the background of limited empirical evidence of effectiveness and longitudinal evaluation of AI systems. Advanced dissemination of nursing practice with AI technologies also calls for modified qualification, education, and informing of nurses, care dependents, and caregivers. Basic knowledge of AI abilities, opportunities, and limitations, as well as limitations concerning data and AI-generated predictions, could become the subject matter for basic nursing education, as well as practice guidelines and information campaigns to enable nurses to take on a mediating role when implementing AI systems in nursing practice.

## Appendix

Multimedia Appendix 1 Search strategy.

Multimedia Appendix 2 Overview of included studies.

Multimedia Appendix 3 Overview of studies with applications in real-world settings.

## Abbreviations

AI: artificial intelligence
ELSI: ethical, legal, and social implication
ICU: intensive care unit
LOE: level of evidence
LOS: length of stay
ML: machine learning
NLP: natural language processing
PRISMA: Preferred Reporting Items for Systematic Reviews and Meta-Analyses
