# Supplementary material for: Application Scenarios for Artificial Intelligence in Nursing Care: Rapid Review
Source: J Med Internet Res. 2021 Nov 29;23(11):e26522. doi: 10.2196/26522 (PMC8669587; doi:10.2196/26522)
Supplement: Multimedia Appendix 1 [file jmir_v23i11e26522_app1.docx]

**Search terms, strategy and number of identified publications**highlighted results were exported to next screening step

**Database PubMed,** [**https://pubmed.ncbi.nlm.nih.gov/**](https://pubmed.ncbi.nlm.nih.gov/)

| **Search number** | **Query** | **Results** |
| --- | --- | --- |
| 1 | "nursing care"[Title/Abstract] | 26,050 |
| 2 | "elderly care"[Title/Abstract] | 1,369 |
| 3 | "care for the elderly"[Title/Abstract] | 1,516 |
| 4 | "home care"[Title/Abstract] | 18,682 |
| 5 | "care service"[Title/Abstract] | 5,852 |
| 6 | "geriatric care"[Title/Abstract] | 1,728 |
| 7 | "gerontolog* care"[Title/Abstract] | 0 |
| 8 | gerontolog* care[Title/Abstract] | 8,625 |
| 9 | "dementia care"[Title/Abstract] | 2,737 |
| 10 | (((((((("dementia care"[Title/Abstract]) OR (gerontolog* care[Title/Abstract])) OR ("gerontolog* care"[Title/Abstract])) OR ("geriatric care"[Title/Abstract])) OR ("care service"[Title/Abstract])) OR ("home care"[Title/Abstract])) OR ("care for the elderly"[Title/Abstract])) OR ("elderly care"[Title/Abstract])) OR ("nursing care"[Title/Abstract]) | 63,282 |
| 11 | "surgical care"[Title/Abstract] | 5,583 |
| 12 | "orthopedic care"[Title/Abstract] | 230 |
| 13 | "social care"[Title/Abstract] | 6,154 |
| 14 | "postoperative care"[Title/Abstract] | 9,107 |
| 15 | "perioperative care"[Title/Abstract] | 3,979 |
| 16 | "preoperative care"[Title/Abstract] | 2,175 |
| 17 | "intensive care"[Title/Abstract] | 142,965 |
| 18 | "critical care"[Title/Abstract] | 30,527 |
| 19 | "intermediate care"[Title/Abstract] | 1,536 |
| 20 | (((((((("intermediate care"[Title/Abstract]) OR ("critical care"[Title/Abstract])) OR ("intensive care"[Title/Abstract])) OR ("preoperative care"[Title/Abstract])) OR ("perioperative care"[Title/Abstract])) OR ("postoperative care"[Title/Abstract])) OR ("postoperative care"[Title/Abstract])) OR ("orthopedic care"[Title/Abstract])) OR ("surgical care"[Title/Abstract]) | 183,598 |
| 21 | "hospital care"[Title/Abstract] | 10,114 |
| 22 | "emergency care"[Title/Abstract] | 9,548 |
| 23 | oncolog* care[Title/Abstract] | 57,679 |
| 24 | "palliative care"[Title/Abstract] | 29,390 |
| 25 | cardiolog* care[Title/Abstract] | 27,991 |
| 26 | "psychiatric care"[Title/Abstract] | 5,190 |
| 27 | psycholog* care[Title/Abstract] | 220,852 |
| 28 | "psychosomatic care"[Title/Abstract] | 73 |
| 29 | neurolog* care[Title/Abstract] | 47,530 |
| 30 | neurosurg* care[Title/Abstract] | 16,314 |
| 31 | urolog* care[Title/Abstract] | 14,195 |
| 32 | ((((((((((urolog* care[Title/Abstract]) OR (neurosurg* care[Title/Abstract])) OR (neurolog* care[Title/Abstract])) OR ("psychosomatic care"[Title/Abstract])) OR (psycholog* care[Title/Abstract])) OR ("psychiatric care"[Title/Abstract])) OR (cardiolog* care[Title/Abstract])) OR ("palliative care"[Title/Abstract])) OR (oncolog* care[Title/Abstract])) OR ("emergency care"[Title/Abstract])) OR ("hospital care"[Title/Abstract]) | 385,103 |
| 33 | pulmon* care[Title/Abstract] | 49,617 |
| 34 | "respiratory care"[Title/Abstract] | 1,920 |
| 35 | ophthalmolog* care[Title/Abstract] | 10,834 |
| 36 | dermatolog* care[Title/Abstract] | 16,002 |
| 37 | gynecolog* care[Title/Abstract] | 29,547 |
| 38 | "obstetric care"[Title/Abstract] | 3,385 |
| 39 | otolaryngolog* care[Title/Abstract] | 9,923 |
| 40 | anesthe* care[Title/Abstract] | 52,365 |
| 41 | nephrolog* care[Title/Abstract] | 13,431 |
| 42 | diabet* care[Title/Abstract] | 65,528 |
| 43 | (((((((((diabet* care[Title/Abstract]) OR (nephrolog* care[Title/Abstract])) OR (anesthe* care[Title/Abstract])) OR (otolaryngolog* care[Title/Abstract])) OR ("obstetric care"[Title/Abstract])) OR (gynecolog* care[Title/Abstract])) OR (dermatolog* care[Title/Abstract])) OR (ophthalmolog* care[Title/Abstract])) OR ("respiratory care"[Title/Abstract])) OR (pulmon* care[Title/Abstract]) | 230,629 |
| 44 | ((((((((((((diabet* care[Title/Abstract]) OR (nephrolog* care[Title/Abstract])) OR (anesthe* care[Title/Abstract])) OR (otolaryngolog* care[Title/Abstract])) OR ("obstetric care"[Title/Abstract])) OR (gynecolog* care[Title/Abstract])) OR (dermatolog* care[Title/Abstract])) OR (ophthalmolog* care[Title/Abstract])) OR ("respiratory care"[Title/Abstract])) OR (pulmon* care[Title/Abstract])) OR (((((((((((urolog* care[Title/Abstract]) OR (neurosurg* care[Title/Abstract])) OR (neurolog* care[Title/Abstract])) OR ("psychosomatic care"[Title/Abstract])) OR (psycholog* care[Title/Abstract])) OR ("psychiatric care"[Title/Abstract])) OR (cardiolog* care[Title/Abstract])) OR ("palliative care"[Title/Abstract])) OR (oncolog* care[Title/Abstract])) OR ("emergency care"[Title/Abstract])) OR ("hospital care"[Title/Abstract]))) OR ((((((((("intermediate care"[Title/Abstract]) OR ("critical care"[Title/Abstract])) OR ("intensive care"[Title/Abstract])) OR ("preoperative care"[Title/Abstract])) OR ("perioperative care"[Title/Abstract])) OR ("postoperative care"[Title/Abstract])) OR ("postoperative care"[Title/Abstract])) OR ("orthopedic care"[Title/Abstract])) OR ("surgical care"[Title/Abstract]))) OR ((((((((("dementia care"[Title/Abstract]) OR (gerontolog* care[Title/Abstract])) OR ("gerontolog* care"[Title/Abstract])) OR ("geriatric care"[Title/Abstract])) OR ("care service"[Title/Abstract])) OR ("home care"[Title/Abstract])) OR ("care for the elderly"[Title/Abstract])) OR ("elderly care"[Title/Abstract])) OR ("nursing care"[Title/Abstract])) | 709,138 |
| 45 | "deep learning"[Title/Abstract] | 9,188 |
| 46 | "big data"[Title/Abstract] | 6,897 |
| 47 | "artificial intelligence"[Title/Abstract] | 7,760 |
| 48 | "AI"[Title/Abstract] | 32,792 |
| 49 | "AI-System"[Title/Abstract] | 94 |
| 50 | "learning system"[Title/Abstract] | 1,139 |
| 51 | "intelligent system"[Title/Abstract] | 291 |
| 52 | "machine learning"[Title/Abstract] | 29,260 |
| 53 | "supervised learning"[Title/Abstract] | 2,503 |
| 54 | "reinforcement learning"[Title/Abstract] | 3,104 |
| 55 | "neuronal network"[Title/Abstract] | 3,784 |
| 56 | "neuronal networks"[Title/Abstract] | 4,846 |
| 57 | "decision-support"[Title/Abstract] | 15,523 |
| 58 | "decision support"[Title/Abstract] | 15,523 |
| 59 | "predictive analytics"[Title/Abstract] | 437 |
| 60 | "bayes learning"[Title/Abstract] | 0 |
| 61 | "Bayes learning"[Title/Abstract] | 0 |
| 62 | "Bayes"[Title/Abstract] | 7,221 |
| 63 | "genetic algorithms"[Title/Abstract] | 1,650 |
| 64 | "metaheuristics"[Title/Abstract] | 139 |
| 65 | "tabu search"[Title/Abstract] | 155 |
| 66 | "simulated annealing"[Title/Abstract] | 3,228 |
| 67 | "support vector machines"[Title/Abstract] | 4,512 |
| 68 | (((((((((((((((((((("support vector machines"[Title/Abstract]) OR ("simulated annealing"[Title/Abstract])) OR ("tabu search"[Title/Abstract])) OR ("metaheuristics"[Title/Abstract])) OR ("genetic algorithms"[Title/Abstract])) OR ("bayes learning"[Title/Abstract])) OR ("predictive analytics"[Title/Abstract])) OR ("decision support"[Title/Abstract])) OR ("decision-support"[Title/Abstract])) OR ("neuronal networks"[Title/Abstract])) OR ("neuronal network"[Title/Abstract])) OR ("reinforcement learning"[Title/Abstract])) OR ("supervised learning"[Title/Abstract])) OR ("machine learning"[Title/Abstract])) OR ("intelligent system"[Title/Abstract])) OR ("learning system"[Title/Abstract])) OR ("AI-System"[Title/Abstract])) OR ("AI"[Title/Abstract])) OR ("artificial intelligence"[Title/Abstract])) OR ("big data"[Title/Abstract])) OR ("deep learning"[Title/Abstract]) | 113,954 |
| 69 | ((((((((((((((((((((("support vector machines"[Title/Abstract]) OR ("simulated annealing"[Title/Abstract])) OR ("tabu search"[Title/Abstract])) OR ("metaheuristics"[Title/Abstract])) OR ("genetic algorithms"[Title/Abstract])) OR ("bayes learning"[Title/Abstract])) OR ("predictive analytics"[Title/Abstract])) OR ("decision support"[Title/Abstract])) OR ("decision-support"[Title/Abstract])) OR ("neuronal networks"[Title/Abstract])) OR ("neuronal network"[Title/Abstract])) OR ("reinforcement learning"[Title/Abstract])) OR ("supervised learning"[Title/Abstract])) OR ("machine learning"[Title/Abstract])) OR ("intelligent system"[Title/Abstract])) OR ("learning system"[Title/Abstract])) OR ("AI-System"[Title/Abstract])) OR ("AI"[Title/Abstract])) OR ("artificial intelligence"[Title/Abstract])) OR ("big data"[Title/Abstract])) OR ("deep learning"[Title/Abstract])) AND (((((((((((((diabet* care[Title/Abstract]) OR (nephrolog* care[Title/Abstract])) OR (anesthe* care[Title/Abstract])) OR (otolaryngolog* care[Title/Abstract])) OR ("obstetric care"[Title/Abstract])) OR (gynecolog* care[Title/Abstract])) OR (dermatolog* care[Title/Abstract])) OR (ophthalmolog* care[Title/Abstract])) OR ("respiratory care"[Title/Abstract])) OR (pulmon* care[Title/Abstract])) OR (((((((((((urolog* care[Title/Abstract]) OR (neurosurg* care[Title/Abstract])) OR (neurolog* care[Title/Abstract])) OR ("psychosomatic care"[Title/Abstract])) OR (psycholog* care[Title/Abstract])) OR ("psychiatric care"[Title/Abstract])) OR (cardiolog* care[Title/Abstract])) OR ("palliative care"[Title/Abstract])) OR (oncolog* care[Title/Abstract])) OR ("emergency care"[Title/Abstract])) OR ("hospital care"[Title/Abstract]))) OR ((((((((("intermediate care"[Title/Abstract]) OR ("critical care"[Title/Abstract])) OR ("intensive care"[Title/Abstract])) OR ("preoperative care"[Title/Abstract])) OR ("perioperative care"[Title/Abstract])) OR ("postoperative care"[Title/Abstract])) OR ("postoperative care"[Title/Abstract])) OR ("orthopedic care"[Title/Abstract])) OR ("surgical care"[Title/Abstract]))) OR ((((((((("dementia care"[Title/Abstract]) OR (gerontolog* care[Title/Abstract])) OR ("gerontolog* care"[Title/Abstract])) OR ("geriatric care"[Title/Abstract])) OR ("care service"[Title/Abstract])) OR ("home care"[Title/Abstract])) OR ("care for the elderly"[Title/Abstract])) OR ("elderly care"[Title/Abstract])) OR ("nursing care"[Title/Abstract]))) | 4,990 |

| **Search number** | **Query** | **Results** |
| --- | --- | --- |
| 1 | Pflege[Title/Abstract] | 99 |
| 2 | Pflege | 8,302 |
| 3 | "Künstliche Intelligenz"[Title/Abstract] | 0 |
| 4 | "KI"[Title/Abstract] | 57,701 |
| 5 | "KI-System"[Title/Abstract] | 16 |
| 6 | Lernende* System[Title/Abstract] | 2 |
| 7 | Intelligente* System[Title/Abstract] | 50 |
| 8 | maschinelles Lernen[Title/Abstract] | 3 |
| 9 | Überwachtes Lernen[Title/Abstract] | 17 |
| 10 | verstärkendes Lernen[Title/Abstract] | 17 |
| 11 | "Deep learning"[Title/Abstract] | 9,188 |
| 12 | "Big Data"[Title/Abstract] | 6,897 |
| 13 | "Neuronale Netze"[Title/Abstract] | 0 |
| 14 | "Entscheidungsunterstützung"[Title/Abstract] | 2 |
| 15 | ((((((((((("Entscheidungsunterstützung"[Title/Abstract]) OR ("Neuronale Netze"[Title/Abstract])) OR ("Big Data"[Title/Abstract])) OR ("Deep learning"[Title/Abstract])) OR (verstärkendes Lernen[Title/Abstract])) OR (Überwachtes Lernen[Title/Abstract])) OR (maschinelles Lernen[Title/Abstract])) OR (Intelligente* System[Title/Abstract])) OR (Lernende* System[Title/Abstract])) OR ("KI-System"[Title/Abstract])) OR ("KI"[Title/Abstract])) OR ("Künstliche Intelligenz"[Title/Abstract]) | 73,599 |
| 16 | (((((((((((("Entscheidungsunterstützung"[Title/Abstract]) OR ("Neuronale Netze"[Title/Abstract])) OR ("Big Data"[Title/Abstract])) OR ("Deep learning"[Title/Abstract])) OR (verstärkendes Lernen[Title/Abstract])) OR (überwachtes Lernen[Title/Abstract])) OR (maschinelles Lernen[Title/Abstract])) OR (Intelligente* System[Title/Abstract])) OR (Lernende* System[Title/Abstract])) OR ("KI-System"[Title/Abstract])) OR ("KI"[Title/Abstract])) OR ("Künstliche Intelligenz"[Title/Abstract])) AND (Pflege) | 3 |

**Database CINAHL,** [**https://www.ebsco.com/products/research-databases/cinahl-database**](https://www.ebsco.com/products/research-databases/cinahl-database)

| **Search Number** | **Search** | **Results** |
| --- | --- | --- |
| S12 | S2 AND S11 | 1,531 |
|  |  |  |
|  |  |  |
| S11 | AB artificial intelligence OR AB deep learning OR AB neuronal network OR AB machine learning OR AB supervised learning OR AB reinforcement learning OR AB predictive analytics OR AB bayes OR AB genetic algorithm | 8,558 |
|  |  |  |
|  |  |  |
| S10 | AB artificial intelligence OR AB deep learning OR AB neuronal OR AB learning OR AB intelligent OR AB machine learning OR AB supervised OR AB reinforcement OR AB predictive analytics OR AB bayes OR AB genetic algorithm | 130,159 |
|  |  |  |
|  |  |  |
| S9 | S2 AND S4 | 37,608 |
|  |  |  |
|  |  |  |
| S8 | S2 AND S3 | 291 |
|  |  |  |
|  |  |  |
| S7 | S2 AND S5 | 179 |
|  |  |  |
|  |  |  |
| S6 | S2 AND S4 | 37,608 |
|  |  |  |
|  |  |  |
| S5 | AB metaheuristics OR AB tabu search OR AB simulated annealing OR AB support vector machine | 1,707 |
|  |  |  |
|  |  |  |
| S4 | AB artificial intelligence OR AB deep learning OR AB neuronal OR AB ai OR AB learning OR AB intelligent OR AB machine learning OR AB supervised OR AB reinforcement OR AB predictive analytics OR AB bayes OR AB genetic algorithm | 114,771 |
|  |  |  |
|  |  |  |
| S3 | AB künstliche intelligenz OR AB KI OR AB intelligentes system OR AB maschinell OR AB überwacht OR verstärkend | 1,501 |
|  |  |  |
|  |  |  |
| S2 | AB nursing OR AB elderly OR AB care OR AB nurse OR AB ( dementia or alzheimer ) OR Pflege | 761,022 |
|  |  |  |
|  |  |  |
| S1 | AB nursing OR AB elderly OR AB care OR AB nurse OR AB ( dementia or alzheimer ) OR Pflege | 947,692 |
|  |  |  |
|  |  |  |

**ACM Digital Library, searched the ACM Full-Text Collection 591,530 Records,** [**https://dl.acm.org/search/advanced**](https://dl.acm.org/search/advanced)

| **Search** | **Results** |
| --- | --- |
| [[Publication Title: "nursing care"] OR [Publication Title: "elderly care"] OR [Publication Title: "nursing"] OR [Publication Title: "home care"] OR [Publication Title: nurs*] OR [Publication Title: "dementia care"] OR [Publication Title: "care for the elderly"] OR [Publication Title: "geriatric care"]] AND [[Abstract: "nursing care"] OR [Abstract: "elderly care"] OR [Abstract: "nursing"] OR [Abstract: "home care"] OR [Abstract: nurs*] OR [Abstract: "dementia care"] OR [Abstract: "care for the elderly"] OR [Abstract: "geriatric care"]] AND [Publication Date: (01/01/2012 TO 05/31/2020)] | 301 |
| [[Publication Title: "nursing care"] OR [Publication Title: "elderly care"] OR [Publication Title: "care for the elderly"] OR [Publication Title: "home care"] OR [Publication Title: "care service"] OR [Publication Title: "geriatric care"] OR [Publication Title: gerontolog* care] OR [Publication Title: "dementia care"] OR [Publication Title: "surgical care"] OR [Publication Title: "orthopedic care"] OR [Publication Title: "social care"] OR [Publication Title: "postoperative care"] OR [Publication Title: "preoperative care"] OR [Publication Title: "perioperative care"] OR [Publication Title: "intensive care"] OR [Publication Title: "critical care"] OR [Publication Title: "intermediate care"] OR [Publication Title: "hospital care"] OR [Publication Title: "emergency care"] OR [Publication Title: oncolog* care] OR [Publication Title: "palliative care"] OR [Publication Title: cardiol* care] OR [Publication Title: "psychiatric care"] OR [Publication Title: psycholog* care] OR [Publication Title: "psychosomatic care"] OR [Publication Title: neurolog* care] OR [Publication Title: neurosurg* care] OR [Publication Title: urolog* care] OR [Publication Title: "pulmonary care"] OR [Publication Title: "respiratory care"] OR [Publication Title: ophthalmolog* care] OR [Publication Title: dermatolog* care] OR [Publication Title: "gynecologic care"] OR [Publication Title: "obstetric care"] OR [Publication Title: otolaryngolog* care] OR [Publication Title: anesthe* care] OR [Publication Title: nephrolog* care] OR [Publication Title: diabet* care]] AND [[Abstract: "nursing care"] OR [Abstract: "elderly care"] OR [Abstract: "care for the elderly"] OR [Abstract: "home care"] OR [Abstract: "care service"] OR [Abstract: "geriatric care"] OR [Abstract: gerontolog* care] OR [Abstract: "dementia care"] OR [Abstract: "surgical care"] OR [Abstract: "orthopedic care"] OR [Abstract: "social care"] OR [Abstract: "postoperative care"] OR [Abstract: "preoperative care"] OR [Abstract: "perioperative care"] OR [Abstract: "intensive care"] OR [Abstract: "critical care"] OR [Abstract: "intermediate care"] OR [Abstract: "hospital care"] OR [Abstract: "emergency care"] OR [Abstract: oncolog* care] OR [Abstract: "palliative care"] OR [Abstract: cardiol* care] OR [Abstract: "psychiatric care"] OR [Abstract: psycholog* care] OR [Abstract: "psychosomatic care"] OR [Abstract: neurolog* care] OR [Abstract: neurosurg* care] OR [Abstract: urolog* care] OR [Abstract: "pulmonary care"] OR [Abstract: "respiratory care"] OR [Abstract: ophthalmolog* care] OR [Abstract: dermatolog* care] OR [Abstract: "gynecologic care"] OR [Abstract: "obstetric care"] OR [Abstract: otolaryngolog* care] OR [Abstract: anesthe* care] OR [Abstract: nephrolog* care] OR [Abstract: diabet* care]] AND [Publication Date: (01/01/2005 TO 05/31/2020)] | 1,590 |
| [[Publication Title: künstliche intelligenz] OR [Publication Title: ki] OR [Publication Title: ki-system] OR [Publication Title: lernende* system] OR [Publication Title: intelligente* system] OR [Publication Title: maschinelles lernen] OR [Publication Title: überwachtes lernen] OR [Publication Title: verstärkendes lernen] OR [Publication Title: deep learning] OR [Publication Title: big data] OR [Publication Title: neuronale netze] OR [Publication Title: entscheidungsunterstützung] OR [Publication Title: artificial intelligence] OR [Publication Title: ai] OR [Publication Title: ai-system] OR [Publication Title: learning system] OR [Publication Title: intelligent system] OR [Publication Title: machine learning] OR [Publication Title: supervised learning] OR [Publication Title: reinforcement learning] OR [Publication Title: deep learning] OR [Publication Title: neuronal networks] OR [Publication Title: decision-support] OR [Publication Title: predictive analytics] OR [Publication Title: bayes learning] OR [Publication Title: genetic algorithms] OR [Publication Title: metaheuristics] OR [Publication Title: tabu search] OR [Publication Title: simulated annealing] OR [Publication Title: support vector machines]] AND [[Abstract: künstliche intelligenz] OR [Abstract: ki] OR [Abstract: ki-system] OR [Abstract: lernende* system] OR [Abstract: intelligente* system] OR [Abstract: maschinelles lernen] OR [Abstract: überwachtes lernen] OR [Abstract: verstärkendes lernen] OR [Abstract: deep learning] OR [Abstract: big data] OR [Abstract: neuronale netze] OR [Abstract: entscheidungsunterstützung] OR [Abstract: artificial intelligence] OR [Abstract: ai] OR [Abstract: ai-system] OR [Abstract: learning system] OR [Abstract: intelligent system] OR [Abstract: machine learning] OR [Abstract: supervised learning] OR [Abstract: reinforcement learning] OR [Abstract: deep learning] OR [Abstract: neuronal networks] OR [Abstract: decision-support] OR [Abstract: predictive analytics] OR [Abstract: bayes learning] OR [Abstract: genetic algorithms] OR [Abstract: metaheuristics] OR [Abstract: tabu search] OR [Abstract: simulated annealing] OR [Abstract: support vector machines]] AND [Publication Date: (01/01/2005 TO 05/31/2020)] | 216,835 |
| [Results for: [[Publication Title: pflege] OR [Publication Title: nursing care] OR [Publication Title: elderly care] OR [Publication Title: care for the elderly] OR [Publication Title: home care] OR [Publication Title: care service] OR [Publication Title: geriatric care] OR [Publication Title: gerontolog* care] OR [Publication Title: dementia care] OR [Publication Title: surgical care] OR [Publication Title: orthopedic care] OR [Publication Title: social care] OR [Publication Title: postoperative care] OR [Publication Title: preoperative care] OR [Publication Title: perioperative care] OR [Publication Title: intensive care] OR [Publication Title: critical care] OR [Publication Title: intermediate care] OR [Publication Title: hospital care] OR [Publication Title: emergency care] OR [Publication Title: oncolog* care] OR [Publication Title: palliative care] OR [Publication Title: cardiol* care] OR [Publication Title: psychiatric care] OR [Publication Title: psycholog* care] OR [Publication Title: psychosomatic care] OR [Publication Title: neurolog* care] OR [Publication Title: neurosurg* care] OR [Publication Title: urolog* care] OR [Publication Title: pulmonary care] OR [Publication Title: respiratory care] OR [Publication Title: ophthalmolog* care] OR [Publication Title: dermatolog* care] OR [Publication Title: gynecologic care] OR [Publication Title: obstetric care] OR [Publication Title: otolaryngolog* care] OR [Publication Title: anesthe* care] OR [Publication Title: nephrolog* care] OR [Publication Title: diabet* care]] AND [[Publication Title: pflege] OR [Publication Title: nursing care] OR [Publication Title: elderly care] OR [Publication Title: care for the elderly] OR [Publication Title: home care] OR [Publication Title: care service] OR [Publication Title: geriatric care] OR [Publication Title: gerontolog* care] OR [Publication Title: dementia care] OR [Publication Title: surgical care] OR [Publication Title: orthopedic care] OR [Publication Title: social care] OR [Publication Title: postoperative care] OR [Publication Title: preoperative care] OR [Publication Title: perioperative care] OR [Publication Title: intensive care] OR [Publication Title: critical care] OR [Publication Title: intermediate care] OR [Publication Title: hospital care] OR [Publication Title: emergency care] OR [Publication Title: oncolog* care] OR [Publication Title: palliative care] OR [Publication Title: cardiol* care] OR [Publication Title: psychiatric care] OR [Publication Title: psycholog* care] OR [Publication Title: psychosomatic care] OR [Publication Title: neurolog* care] OR [Publication Title: neurosurg* care] OR [Publication Title: urolog* care] OR [Publication Title: pulmonary care] OR [Publication Title: respiratory care] OR [Publication Title: ophthalmolog* care] OR [Publication Title: dermatolog* care] OR [Publication Title: gynecologic care] OR [Publication Title: obstetric care] OR [Publication Title: otolaryngolog* care] OR [Publication Title: anesthe* care] OR [Publication Title: nephrolog* care] OR [Publication Title: diabet* care]] AND [Publication Date: (01/01/2005 TO 06/30/2020)]](https://dl.acm.org/search/advanced?AfterMonth=1&AfterYear=2005&BeforeMonth=6&BeforeYear=2020&content=standard&countTerms=true&expand=dl&field1=Title&field2=Title&target=default&text1=Pflege+OR+nursing+care+OR+elderly+care+OR+care+for+the+elderly+OR+home+care+OR+care+service+OR+geriatric+care+OR+gerontolog*+care+OR+dementia+care+OR+surgical+care+OR+orthopedic+care+OR+social+care+OR+postoperative+care+OR+preoperative+care+OR+perioperative+care+OR+intensive+care+OR+critical+care+OR+intermediate+care+OR+hospital+care+OR+emergency+care+OR+oncolog*+care+OR+palliative+care+OR+cardiol*+care+OR+psychiatric+care+OR++psycholog*+care+OR+psychosomatic+care+OR+neurolog*+care+OR+neurosurg*+care+OR+urolog*+care+OR+pulmonary+care+OR+respiratory+care+OR+ophthalmolog*+care+OR+dermatolog*+care+OR+gynecologic+care+OR+obstetric+care+OR+otolaryngolog*+care+OR+anesthe*+care+OR+nephrolog*+care+OR+diabet*+care&text2=Pflege+OR+nursing+care+OR+elderly+care+OR+care+for+the+elderly+OR+home+care+OR+care+service+OR+geriatric+care+OR+gerontolog*+care+OR+dementia+care+OR+surgical+care+OR+orthopedic+care+OR+social+care+OR+postoperative+care+OR+preoperative+care+OR+perioperative+care+OR+intensive+care+OR+critical+care+OR+intermediate+care+OR+hospital+care+OR+emergency+care+OR+oncolog*+care+OR+palliative+care+OR+cardiol*+care+OR+psychiatric+care+OR++psycholog*+care+OR+psychosomatic+care+OR+neurolog*+care+OR+neurosurg*+care+OR+urolog*+care+OR+pulmonary+care+OR+respiratory+care+OR+ophthalmolog*+care+OR+dermatolog*+care+OR+gynecologic+care+OR+obstetric+care+OR+otolaryngolog*+care+OR+anesthe*+care+OR+nephrolog*+care+OR+diabet*+care&editQuery=true&fillQuickSearch=false) | 35,185 |
| [[Publication Title: pflege] OR [Publication Title: nursing care] OR [Publication Title: elderly care] OR [Publication Title: care for the elderly] OR [Publication Title: home care] OR [Publication Title: care service] OR [Publication Title: geriatric care] OR [Publication Title: gerontolog*care] OR [Publication Title: dementia care] OR [Publication Title: surgical care] OR [Publication Title: orthopedic care] OR [Publication Title: social care] OR [Publication Title: postoperative care] OR [Publication Title: preoperative care] OR [Publication Title: perioperative care] OR [Publication Title: intensive care] OR [Publication Title: critical care] OR [Publication Title: intermediate care] OR [Publication Title: hospital care] OR [Publication Title: emergency care] OR [Publication Title: oncolog* care] OR [Publication Title: palliative care] OR [Publication Title: cardiol* care] OR [Publication Title: psychiatric care] OR [Publication Title: psycholog* care] OR [Publication Title: psychosomatic care] OR [Publication Title: neurolog* care] OR [Publication Title: neurosurg* care] OR [Publication Title: urolog* care] OR [Publication Title: pulmonary care] OR [Publication Title: respiratory care] OR [Publication Title: ophthalmolog* care] OR [Publication Title: dermatolog* care] OR [Publication Title: gynecologic care] OR [Publication Title: obstetric care] OR [Publication Title: otolaryngolog* care] OR [Publication Title: anesthe* care] OR [Publication Title: nephrolog* care] OR [Publication Title: diabet* care]] AND [[Publication Title: künstliche intelligenz] OR [Publication Title: ki] OR [Publication Title: ki-system] OR [Publication Title: lernende* system] OR [Publication Title: intelligente* system] OR [Publication Title: maschinelles lernen] OR [Publication Title: überwachtes lernen] OR [Publication Title: verstärkendes lernen] OR [Publication Title: deep learning] OR [Publication Title: big data] OR [Publication Title: neuronale netze] OR [Publication Title: entscheidungsunterstützung] OR [Publication Title: artificial intelligence] OR [Publication Title: ai] OR [Publication Title: ai-system] OR [Publication Title: learning system] OR [Publication Title: intelligent system] OR [Publication Title: machine learning] OR [Publication Title: supervised learning] OR [Publication Title: reinforcement learning] OR [Publication Title: deep learning] OR [Publication Title: neuronal networks] OR [Publication Title: decision-support] OR [Publication Title: predictive analytics] OR [Publication Title: bayes learning] OR [Publication Title: genetic algorithms] OR [Publication Title: metaheuristics] OR [Publication Title: tabu search] OR [Publication Title: simulated annealing] OR [Publication Title: support vector machine]] AND [[Abstract: pflege] OR [Abstract: nursing care] OR [Abstract: elderly care] OR [Abstract: care for the elderly] OR [Abstract: home care] OR [Abstract: care service] OR [Abstract: geriatric care] OR [Abstract: gerontolog* care] OR [Abstract: dementia care] OR [Abstract: surgical care] OR [Abstract: orthopedic care] OR [Abstract: social care] OR [Abstract: postoperative care] OR [Abstract: preoperative care] OR [Abstract: perioperative care] OR [Abstract: intensive care] OR [Abstract: critical care] OR [Abstract: intermediate care] OR [Abstract: hospital care] OR [Abstract: emergency care] OR [Abstract: oncolog*care] OR [Abstract: palliative care] OR [Abstract: cardiol* care] OR [Abstract: psychiatric care] OR [Abstract: psycholog* care] OR [Abstract: psychosomatic care] OR [Abstract: neurolog* care] OR [Abstract: neurosurg* care] OR [Abstract: urolog* care] OR [Abstract: pulmonary care] OR [Abstract: respiratory care] OR [Abstract: ophthalmolog* care] OR [Abstract: dermatolog* care] OR [Abstract: gynecologic care] OR [Abstract: obstetric care] OR [Abstract: otolaryngolog* care] OR [Abstract: anesthe* care] OR [Abstract: nephrolog* care] OR [Abstract: diabet* care]] AND [[Abstract: künstliche intelligenz] OR [Abstract: ki] OR [Abstract: ki-system] OR [Abstract: lernende* system] OR [Abstract: intelligente* system] OR [Abstract: maschinelles lernen] OR [Abstract: überwachtes lernen] OR [Abstract: verstärkendes lernen] OR [Abstract: deep learning] OR [Abstract: big data] OR [Abstract: neuronale netze] OR [Abstract: entscheidungsunterstützung] OR [Abstract: artificial intelligence] OR [Abstract: ai] OR [Abstract: ai-system] OR [Abstract: learning system] OR [Abstract: intelligent system] OR [Abstract: machine learning] OR [Abstract: supervised learning] OR [Abstract: reinforcement learning] OR [Abstract: deep learning] OR [Abstract: neuronal networks] OR [Abstract: decision-support] OR [Abstract: predictive analytics] OR [Abstract: bayes learning] OR [Abstract: genetic algorithms] OR [Abstract: metaheuristics] OR [Abstract: tabu search] OR [Abstract: simulated annealing] OR [Abstract: support vector machine]] AND [Publication Date: (01/01/2005 TO 06/30/2020)] | 21,309 |
| [[[Publication Title: pflege] OR [Publication Title: nursing care] OR [Publication Title: elderly care] OR [Publication Title: care for the elderly] OR [Publication Title: home care] OR [Publication Title: care service] OR [Publication Title: geriatric care] OR [Publication Title: gerontolog* care] OR [Publication Title: dementia care] OR [Publication Title: nurs*]] AND [[Publication Title: künstliche intelligenz] OR [Publication Title: ki] OR [Publication Title: ki-system] OR [Publication Title: lernende* system] OR [Publication Title: intelligente* system] OR [Publication Title: maschinelles lernen] OR [Publication Title: überwachtes lernen] OR [Publication Title: verstärkendes lernen] OR [Publication Title: deep learning] OR [Publication Title: big data] OR [Publication Title: neuronale netze] OR [Publication Title: entscheidungsunterstützung] OR [Publication Title: artificial intelligence] OR [Publication Title: ai] OR [Publication Title: ai-system] OR [Publication Title: learning system] OR [Publication Title: intelligent system] OR [Publication Title: machine learning] OR [Publication Title: supervised learning] OR [Publication Title: reinforcement learning] OR [Publication Title: deep learning] OR [Publication Title: neuronal networks] OR [Publication Title: decision-support] OR [Publication Title: predictive analytics] OR [Publication Title: bayes learning] OR [Publication Title: genetic algorithms] OR [Publication Title: metaheuristics] OR [Publication Title: tabu search] OR [Publication Title: simulated annealing] OR [Publication Title: support vector machine]] AND [[Abstract: pflege] OR [Abstract: nursing care] OR [Abstract: elderly care] OR [Abstract: care for the elderly] OR [Abstract: home care] OR [Abstract: care service] OR [Abstract: geriatric care] OR [Abstract: gerontolog* care] OR [Abstract: dementia care] OR [Abstract: nurs*]] AND [[Abstract: künstliche intelligenz] OR [Abstract: ki] OR [Abstract: ki-system] OR [Abstract: lernende* system] OR [Abstract: intelligente* system] OR [Abstract: maschinelles lernen] OR [Abstract: überwachtes lernen] OR [Abstract: verstärkendes lernen] OR [Abstract: deep learning] OR [Abstract: big data] OR [Abstract: neuronale netze] OR [Abstract: entscheidungsunterstützung] OR [Abstract: artificial intelligence] OR [Abstract: ai] OR [Abstract: ai-system] OR [Abstract: learning system] OR [Abstract: intelligent system] OR [Abstract: machine learning] OR [Abstract: supervised learning] OR [Abstract: reinforcement learning] OR [Abstract: deep learning] OR [Abstract: neuronal networks] OR [Abstract: decision-support] OR [Abstract: predictive analytics] OR [Abstract: bayes learning] OR [Abstract: genetic algorithms] OR [Abstract: metaheuristics] OR [Abstract: tabu search] OR [Abstract: simulated annealing] OR [Abstract: support vector machine]] AND [Publication Date: (01/01/2005 TO 06/30/2020)]](https://dl.acm.org/search/advanced?AfterMonth=1&AfterYear=2005&BeforeMonth=6&BeforeYear=2020&content=standard&countTerms=true&expand=dl&field1=Title&field2=Abstract&target=default&text1=%28Pflege+OR+nursing+care+OR+elderly+care+OR+care+for+the+elderly+OR+home+care+OR+care+service+OR+geriatric+care+OR+gerontolog*+care+OR+dementia+care+OR+nurs*%29++AND++%28K%C3%BCnstliche+Intelligenz+OR+KI+OR+KI-System+OR+Lernende*+System+OR+Intelligente*+System+OR+maschinelles+Lernen+OR+%C3%BCberwachtes+Lernen+OR+verst%C3%A4rkendes+Lernen+OR+Deep+learning+OR+Big+Data+OR+Neuronale+Netze+OR+Entscheidungsunterst%C3%BCtzung+OR+artificial+intelligence+OR+AI+OR+AI-System+OR+learning+system+OR+intelligent+system+OR+machine+learning+OR+supervised+learning+OR+reinforcement+Learning+OR+Deep+Learning+OR+Neuronal+networks+OR+Decision-Support++OR+predictive+analytics+OR+Bayes+learning+OR+Genetic+algorithms+OR+Metaheuristics+OR+tabu+search+OR+simulated+annealing+OR+Support+vector+machine%29&text2=%28Pflege+OR+nursing+care+OR+elderly+care+OR+care+for+the+elderly+OR+home+care+OR+care+service+OR+geriatric+care+OR+gerontolog*+care+OR+dementia+care+OR+nurs*%29++AND++%28K%C3%BCnstliche+Intelligenz+OR+KI+OR+KI-System+OR+Lernende*+System+OR+Intelligente*+System+OR+maschinelles+Lernen+OR+%C3%BCberwachtes+Lernen+OR+verst%C3%A4rkendes+Lernen+OR+Deep+learning+OR+Big+Data+OR+Neuronale+Netze+OR+Entscheidungsunterst%C3%BCtzung+OR+artificial+intelligence+OR+AI+OR+AI-System+OR+learning+system+OR+intelligent+system+OR+machine+learning+OR+supervised+learning+OR+reinforcement+Learning+OR+Deep+Learning+OR+Neuronal+networks+OR+Decision-Support++OR+predictive+analytics+OR+Bayes+learning+OR+Genetic+algorithms+OR+Metaheuristics+OR+tabu+search+OR+simulated+annealing+OR+Support+vector+machine%29&editQuery=true&fillQuickSearch=false) | 8,806 |
| [[Publication Title: pflege] OR [Publication Title: nursing care] OR [Publication Title: elderly care] OR [Publication Title: care for the elderly] OR [Publication Title: home care] OR [Publication Title: care service] OR [Publication Title: geriatric care] OR [Publication Title: gerontolog* care] OR [Publication Title: dementia care] OR [Publication Title: nurs*]] AND [[Publication Title: künstliche intelligenz] OR [Publication Title: ki] OR [Publication Title: ki-system] OR [Publication Title: lernende* system] OR [Publication Title: intelligente* system] OR [Publication Title: maschinelles lernen] OR [Publication Title: überwachtes lernen] OR [Publication Title: verstärkendes lernen] OR [Publication Title: deep learning] OR [Publication Title: big data] OR [Publication Title: neuronale netze] OR [Publication Title: entscheidungsunterstützung] OR [Publication Title: artificial intelligence] OR [Publication Title: ai] OR [Publication Title: ai-system] OR [Publication Title: learning system] OR [Publication Title: intelligent system] OR [Publication Title: machine learning] OR [Publication Title: supervised learning] OR [Publication Title: reinforcement learning] OR [Publication Title: deep learning] OR [Publication Title: neuronal networks] OR [Publication Title: predictive analytics] OR [Publication Title: bayes learning] OR [Publication Title: genetic algorithms] OR [Publication Title: metaheuristics] OR [Publication Title: tabu search] OR [Publication Title: simulated annealing] OR [Publication Title: support vector machine]] AND [[Abstract: pflege] OR [Abstract: nursing care] OR [Abstract: elderly care] OR [Abstract: care for the elderly] OR [Abstract: home care] OR [Abstract: care service] OR [Abstract: geriatric care] OR [Abstract: gerontolog* care] OR [Abstract: dementia care] OR [Abstract: nurs*]] AND [[Abstract: künstliche intelligenz] OR [Abstract: ki] OR [Abstract: ki-system] OR [Abstract: lernende* system] OR [Abstract: intelligente* system] OR [Abstract: maschinelles lernen] OR [Abstract: überwachtes lernen] OR [Abstract: verstärkendes lernen] OR [Abstract: deep learning] OR [Abstract: big data] OR [Abstract: neuronale netze] OR [Abstract: entscheidungsunterstützung] OR [Abstract: artificial intelligence] OR [Abstract: ai] OR [Abstract: ai-system] OR [Abstract: learning system] OR [Abstract: intelligent system] OR [Abstract: machine learning] OR [Abstract: supervised learning] OR [Abstract: reinforcement learning] OR [Abstract: deep learning] OR [Abstract: neuronal networks] OR [Abstract: predictive analytics] OR [Abstract: bayes learning] OR [Abstract: genetic algorithms] OR [Abstract: metaheuristics] OR [Abstract: tabu search] OR [Abstract: simulated annealing] OR [Abstract: support vector machine]] AND [Publication Date: (01/01/2005 TO 06/30/2020)] | 8779 |
| [[Publication Title: pflege] OR [Publication Title: "nursing care"] OR [Publication Title: "elderly care"] OR [Publication Title: "care for the elderly"] OR [Publication Title: "home care"] OR [Publication Title: "care service"] OR [Publication Title: "geriatric care"] OR [Publication Title: gerontolog* care] OR [Publication Title: "dementia care"] OR [Publication Title: nurs*]] AND [[Publication Title: "künstliche intelligenz"] OR [Publication Title: "ki"] OR [Publication Title: "ki-system"] OR [Publication Title: lernende* system] OR [Publication Title: intelligente* system] OR [Publication Title: "maschinelles lernen"] OR [Publication Title: "überwachtes lernen"] OR [Publication Title: "verstärkendes lernen"] OR [Publication Title: "deep learning"] OR [Publication Title: "big data"] OR [Publication Title: "neuronale netze"] OR [Publication Title: "neuronales netz"] OR [Publication Title: "entscheidungsunterstützung"] OR [Publication Title: "artificial intelligence"] OR [Publication Title: "ai"] OR [Publication Title: "ai-system"] OR [Publication Title: "learning system"] OR [Publication Title: "intelligent system"] OR [Publication Title: "machine learning"] OR [Publication Title: "supervised learning"] OR [Publication Title: "reinforcement learning"] OR [Publication Title: "decision support"] OR [Publication Title: "deep learning"] OR [Publication Title: "neuronal network"] OR [Publication Title: "neuronal networks"] OR [Publication Title: "predictive analytics"] OR [Publication Title: "bayes learning"] OR [Publication Title: "genetic algorithm"] OR [Publication Title: "metaheuristics"] OR [Publication Title: "tabu search"] OR [Publication Title: "simulated annealing"] OR [Publication Title: "support vector machine"]] AND [[Abstract: pflege] OR [Abstract: "nursing care"] OR [Abstract: "elderly care"] OR [Abstract: "care for the elderly"] OR [Abstract: "home care"] OR [Abstract: "care service"] OR [Abstract: "geriatric care"] OR [Abstract: gerontolog* care] OR [Abstract: "dementia care"] OR [Abstract: nurs*]] AND [[Abstract: "künstliche intelligenz"] OR [Abstract: "ki"] OR [Abstract: "ki-system"] OR [Abstract: lernende* system] OR [Abstract: intelligente* system] OR [Abstract: "maschinelles lernen"] OR [Abstract: "überwachtes lernen"] OR [Abstract: "verstärkendes lernen"] OR [Abstract: "deep learning"] OR [Abstract: "big data"] OR [Abstract: "neuronale netze"] OR [Abstract: "neuronales netz"] OR [Abstract: "entscheidungsunterstützung"] OR [Abstract: "artificial intelligence"] OR [Abstract: "ai"] OR [Abstract: "ai-system"] OR [Abstract: "learning system"] OR [Abstract: "intelligent system"] OR [Abstract: "machine learning"] OR [Abstract: "supervised learning"] OR [Abstract: "reinforcement learning"] OR [Abstract: "decision support"] OR [Abstract: "deep learning"] OR [Abstract: "neuronal network"] OR [Abstract: "neuronal networks"] OR [Abstract: "predictive analytics"] OR [Abstract: "bayes learning"] OR [Abstract: "genetic algorithm"] OR [Abstract: "metaheuristics"] OR [Abstract: "tabu search"] OR [Abstract: "simulated annealing"] OR [Abstract: "support vector machine"]] AND [Publication Date: (01/01/2005 TO 06/30/2020)] | 487 |

**IEEE Xplore,** [**https://ieeexplore.ieee.org/Xplore/home.jsp**](https://ieeexplore.ieee.org/Xplore/home.jsp)

| **Search** | **Results** |
| --- | --- |
| (("Publication Title": Pflege OR "nursing care" OR "elderly care" OR "care for the elderly" OR "home care" OR "care service" OR "geriatric care" OR gerontolog* care OR "dementia care" OR nurs*) refined by:Year:2005-2020 ) | 4,885 |
| (("Abstract":"Künstliche Intelligenz" OR "KI" OR "KI-System" OR Lernende* System OR Intelligente* System OR "maschinelles Lernen" OR "überwachtes Lernen" OR "verstärkendes Lernen" OR "Deep learning" OR "Big Data" OR "Neuronale Netze" OR "Neuronales Netz" OR "Entscheidungsunterstützung" OR "artificial intelligence" OR "AI" OR "AI-System" OR "learning system" OR "intelligent system" OR "machine learning" OR "supervised learning" OR "reinforcement Learning" OR "decision support" "Deep Learning" OR "Neuronal network" OR "neuronal networks" "predictive analytics" OR "Bayes learning" OR "Genetic algorithm" OR "Metaheuristics" OR "tabu search" OR "simulated annealing" OR "Support vector machine") refined by:Year:2005-2020 ) | 324,548 |
| (((Abstract: Pflege OR "nursing care" OR "elderly care" OR "care for the elderly" OR "home care" OR "care service" OR "geriatric care" OR gerontolog* care OR "dementia care" OR nurs*) refined by:Year:2005-2020 )) AND ((Abstract:"Neuronal network" OR "neuronal networks" "predictive analytics" OR "Bayes learning" OR "Genetic algorithm" OR "Metaheuristics" OR "tabu search" OR "simulated annealing" OR "Support vector machine") refined by:Year:2005-2020 ) | 125 |
| (((Abstract: Pflege OR "nursing care" OR "elderly care" OR "care for the elderly" OR "home care" OR "care service" OR "geriatric care" OR gerontolog* care OR "dementia care" OR nurs*) refined by:Year:2005-2020 )) AND ((Abstract:"artificial intelligence" OR "AI" OR "AI-System" OR "learning system" OR "intelligent system" OR "machine learning" OR "supervised learning" OR "reinforcement Learning" OR "decision support" "Deep Learning") refined by:Year:2005-2020 ) | 305 |

**Computer Science Bibliography (dblp),** [**https://dblp.org/**](https://dblp.org/)

| **Search** | **Results** |
| --- | --- |
| Pflege\|care\|nursing\|nurse\|elderly\|geriatric\|gerontolog\|dementia\|nurs Intelligen\|KI\|KI-System\|maschinell\|überwach\|verstärken\|lernen\|learning\|Neuronal\|Entscheidungsunterstützung\|artificial\|intelligence\|AI\|AI-System\|learning\|intelligent\|machine\|supervised\|reinforcement\|decision\|Deep\|predictive\|Bayes\|algorythm\|Metaheuristics\|tabu\|search\|simulated\|annealing\|vector | 418 |

**Association for Information Systems (AIS) eLibrary,** [**https://aisel.aisnet.org/**](https://aisel.aisnet.org/)

| **Search** | **Results** |
| --- | --- |
| abstract:( Pflege OR nursing care OR elderly care OR care for the elderly OR home care OR care service OR geriatric care OR gerontolog* OR dementia care OR nurs* ) AND abstract:( artificial intelligence OR AI OR AI-System OR learning system OR intelligent system OR machine learning OR supervised learning OR reinforcement Learning OR Deep Learning OR Neuronal networks OR Decision-Support OR predictive analytics OR Bayes learning OR Genetic algorithms OR Metaheuristics OR tabu search OR simulated annealing OR Support vector machines ) | 4 |
| abstract:( Pflege OR nursing care OR elderly care OR care for the elderly OR home care OR care service OR geriatric care OR gerontolog* OR dementia care OR nurs* ) | 23717 |
| abstract:( Pflege OR nursing care OR elderly care OR care for the elderly OR home care OR care service OR geriatric care OR gerontolog* OR dementia care OR nurs* ) AND abstract:( Intelligent OR KI OR KI-System OR Lernende* OR maschinell* OR überwacht* OR verstärkend* OR Deep OR learning OR Data OR Neuronal OR Entscheidungsunterstützung OR artificial OR AI OR AI-System OR learning OR intelligent OR machine OR supervised OR reinforcement OR neuronal OR Decision-Support OR predictive OR Bayes OR algorithm OR Metaheuristics OR tabu OR simulated OR annealing OR Support vector ) | 97 |

**Association for the Advancement of Artificial Intelligence (AAAI) conference,** [**https://aaai.org/Library/library.php**](https://aaai.org/Library/library.php)

| **Search** | **Results** |
| --- | --- |
| nurs, elder, care, patient in Journal of Artificial Intelligence Research, AAAI Conference on Artificial Intelligence, AI Magazine, titles only | 2 |

**Association for Computational Linguistics (ACL) conference,** [**https://acl2020.org/**](https://acl2020.org/)

| **Search** | **Results** |
| --- | --- |
| titles only, nurs, elder, care, patient | 0 |

**Conference on Computer Vision and Pattern Recognition (CVPR),** [**http://cvpr2020.thecvf.com/**](http://cvpr2020.thecvf.com/)

| **Search** | **Results** |
| --- | --- |
| titles only, nurs, elder, care, patient | 0 |

**International Conference on Machine Learning (ICML),** [**https://icml.cc/**](https://icml.cc/)

| **Search** | **Results** |
| --- | --- |
| titles only, nurs, elder, care, patient | 0 |

**International Joint Conferences on Artificial Intelligence Organization (IJCAI),** [**https://www.ijcai.org/**](https://www.ijcai.org/)

| **Search** | **Results** |
| --- | --- |
| Titles and abstract nurs, elder, care, patient | 2 |

**Conference of the Association for Computing Machinery’s Special Interest Group on Knowledge Discovery and Data Mining (SIGKDD),** [**https://www.kdd.org/**](https://www.kdd.org/)

| **Search** | **Results** |
| --- | --- |
| Titles and abstract of Proceedings Archives 2017 to 2020: nurs, elder, care, patient | 1 |

**Conference on Neural Information Processing Systems (NeurIPS),** [**https://nips.cc/**](https://nips.cc/)

| **Search** | **Results** |
| --- | --- |
| Titles of Proceedings: nurs, elder, care, patient | 0 |

**International Conference on the Principles of Knowledge Representation and Reasoning (KR),** [**https://www.aaai.org/ocs/index.php/index/index/search**](https://www.aaai.org/ocs/index.php/index/index/search)

| **Search** | **Results** |
| --- | --- |
| Titles and full texts of proceedings, 2009-2020 available: nurs, elder, care, patient | 0 |

**International Joint Conference on Autonomous Agents and Multiagent Systems (AAMAS),** [**http://www.ifaamas.org/proceedings.html**](http://www.ifaamas.org/proceedings.html)

| **Search** | **Results** |
| --- | --- |
| Titles of proceedings, 2007-2020 available: nurs, elder, care, patient | 10 |

**Conference in Uncertainty in Artificial Intelligence (UAI),** [**https://www.auai.org/#proceedings**](https://www.auai.org/#proceedings)

| **Search** | **Results** |
| --- | --- |
| Titles (2005-2011, 2017, 2019, 2020) and full texts (2012-2016, 2018, ) of proceedings: nurs, elder, care, patient | 49 |

**European Conference on Artificial Intelligence (ECAI),** [**https://www.eurai.org/library/ECAI_proceedings**](https://www.eurai.org/library/ECAI_proceedings)

| **Search** | **Results** |
| --- | --- |
| Titles (2018) and fulltexts (2006-2016, 2020) of proceedings: nurs, elder, care, patient | 139 |
