# Supplementary material for: Application Scenarios for Artificial Intelligence in Nursing Care: Rapid Review
Source: J Med Internet Res. 2021 Nov 29;23(11):e26522. doi: 10.2196/26522 (PMC8669587; doi:10.2196/26522)
Supplement: Multimedia Appendix 2 [file jmir_v23i11e26522_app2.docx]

| Reference | Basic Research, Real-world Scenario, Review or Publication on Ethical, Legal or Social Implications (ELSI) | Country | Setting | Type of AI Approach | Application Context | Purpose: monitoring, tracking, classification, prediction, support of … | Level of Evidence |
| --- | --- | --- | --- | --- | --- | --- | --- |
| [1] Alderden et al. 2019 | Real-world | USA | Hospital | Machine Learning | Automated Planning and Scheduling | Pressure ulcers | VI |
| [2] Antoniadi et al. 2020 | Real-world | IRE | Hospital | Machine Learning | Automated Planning and Scheduling | Quality of Life and Wellbeing of Caregivers | IV |
| [3] Brom et al. 2020 | Real-world | USA | Hospital | Machine Learning | Automated Planning and Scheduling | Nursing assessment/care needs assessment | IV |
| [4] Cho et al. 2013 | Real-world | KOR | Hospital | Machine Learning | Image and Signal Processing | Pressure ulcers | III |
| [5] Davoudi et al. 2019 | Real-world | USA | Hospital | Machine Learning | Image and Signal Processing | Activity and Health | IV |
| [6] Marschollek et al. 2008 | Real-world | GER | Hospital | Machine Learning | Image and Signal Processing | Falls | VI |
| [7] Papageorgiou et al. 2017 | Real-world | GRC | Hospital | Machine Learning | Image and Signal Processing | Mobility, other | VI |
| [8] Tawfik et al. 2020 | Real-world | USA | Hospital | Machine Learning | Automated Planning and Scheduling | Nurse Rostering/Scheduling | VI |
| [9] Ye et al. 2020 | Real-world | USA | Hospital | Machine Learning | Image and Signal Processing | Falls | VI |
| [10] Zhang 2007 | Real-world | USA | Hospital | Machine Learning | Automated Planning and Scheduling | Alarms | VI |
| [11] Zampieri et al. 2019 | Real-world | BRA | Hospital | Machine Learning | Automated Planning and Scheduling | Care Coordination and Communication | VI |
| [12] Ambagtsheer et al. 2020 | Real-world | AUS | sLTC | Machine Learning | Automated Planning and Scheduling | Activity and Health | VI |
| [13] Chen et al. 2007 | Real-world | USA | sLTC | Machine Learning | Image and Signal Processing | Activity and Health | VI |
| [14] Tang et al. 2019 | Real-world | HKG | sLTC | Machine Learning | Automated Planning and Scheduling | Care Coordination and Communication | VI |
| [15] Wai et al. 2010 | Real-world | SGP | sLTC | Machine Learning | Image and Signal Processing | Bladder Control | III |
| [16] Xiong et al. 2019 | Real-world | USA | sLTC | Machine Learning | Image and Signal Processing | Falls | III |
| [17] Bajo et al. 2008 | Real-world | SPA | sLTC | Machine Learning | Automated Planning and Scheduling | Nurse Rostering/Scheduling | VI |
| [18] Ala-Kitula et al. 2017 | Real-world | FIN | aLTC | Machine Learning | Natural Language Processing | Care Coordination and Communication | VI |
| [19] Zhou et al. 2006 | Real-world | USA | aLTC | Machine Learning | Automated Planning and Scheduling | Activity and Health | VI |
| [20] Zhu et al. 2007 | Real-world | CAN | aLTC | Machine Learning | Automated Planning and Scheduling | Nursing assessment/care needs assessment | IV |
| [21] Zhu et al. 2007 | Real-world | CAN | aLTC | Machine Learning | Automated Planning and Scheduling | Nursing assessment/care needs assessment | IV |
| [22] Matsuyama et al. 2009 | Real-world | JPN | Daycare | Machine Learning | Image and Signal Processing | Care Coordination and Communication | VI |
| [23] Yang et al. 2019 | Real-world | CAN | Community | Machine Learning | Image and Signal Processing | Falls | VI |
| [24] Yamamoto et al. 2020 | Real-world | JPN | Education facility | Machine Learning | Image and Signal Processing | Activity and Health | III |
|  | | | | | | | |
| [25] Evans et al. 2014 | Real-world | USA | Hospital | Expert System | Image and Signal Processing | Activity and Health | IV |
| [26] Tapia et al. 2010 | Real-world | SPA | sLTC | Expert System | Image and Signal Processing | Activity and Health | VI |
| [27] Alwan 2009 | Real-world | USA | aLTC | Expert System | Image and Signal Processing | Activity and Health | VI |
| [28] Hirdes et al. 2008 | Real-world | CAN | aLTC | Expert System | Automated Planning and Scheduling | Nursing assessment/care needs assessment | IV |
|  | | | | | | | |
| [29] Abdullah & Fakieh 2020 | Real-world | SAU | Hospital | N/A | N/A | N/A | VI |
| [30] Amato et al. 2018 | Real-world | ITA | sLTC | N/A | Image and Signal Processing | Activity and Health | VI |
| [31] Bickmore et al. 2009 | Real-world | USA | Hospital | not specified | Automated Planning and Scheduling | Care Coordination and Communication | VI |
| [32] Carros et al. 2020 | Real-world | GER | SLTC | not specified | Image and Signal Processing | Social Integration and Participation | VI |
| [33] Mervin et al. 2018 | Real-world | AUS | sLTC | not specified | Image and Signal Processing | Social Integration and Participation | II |
| [34] Gonzalez-Usach et al. 2017 | Real-world | SPA | sLTC | not specified | Image and Signal Processing | Activity and Health | concept |
| [35] Viswanathan et al. 2012 | Real-world | CAN | sLTC | not specified | Image and Signal Processing | Mobility, other | VI |
|  | | | | | | | |
| [36] Abe et al. 2006 | Basic | JPN | Hospital | Machine Learning | not specified | Care Coordination and Communication | concept |
| [37] Annangi et al. 2016 | Basic | NOR | Hospital | Machine Learning | Image and Signal Processing | Bladder Control | VI |
| [38] Antink et al. 2016 | Basic | GER | Hospital | Machine Learning | Image and Signal Processing | Alarms | VI |
| [39] Au-Yeung et al. 2019 | Basic | USA | Hospital | Machine Learning | Image and Signal Processing | Alarms | VI |
| [40] Celi et al. 2008 | Basic | USA | Hospital | Machine Learning | Automated Planning and Scheduling | Parenteral or Enteral Nutrition and Fluid Intake | VI |
| [41] Cismondi et al. 2012 | Basic | USA | Hospital | Machine Learning | Image and Signal Processing | Care Coordination and Communication | VI |
| [42] Clavelle et al. 2019 | Basic | USA | Hospital | Machine Learning | Natural Language Processing | Care Coordination and Communication | VI |
| [43] Cramer et al. 2019 | Basic | USA | Hospital | Machine Learning | Automated Planning and Scheduling | Pressure ulcers | IV |
| [44] Crump et al. 2009 | Basic | USA | Hospital | Machine Learning | Image and Signal Processing | Nursing assessment/care needs assessment | VI |
| [45] Durieux et al. 2018 | Basic | USA | Hospital | Machine Learning | Image and Signal Processing | Care Coordination and Communication | VI |
| [46] Eerikäinen et al. 2016 | Basic | NLD | Hospital | Machine Learning | Image and Signal Processing | Alarms | VI |
| [47] Feng & Narayanan 2020 | Basic | USA | Hospital | Machine Learning | Image and Signal Processing | Activity and Health | VI |
| [48] Gerka et al. 2018 | Basic | GER | Hospital | Machine Learning | Image and Signal Processing | Activity and Health | VI |
| [49] Hever et al. 2019 | Basic | ISR | Hospital | Machine Learning | Image and Signal Processing | Alarms | IV |
| [50] Hiissa et al. 2007 | Basic | FIN | Hospital | Machine Learning | Natural Language Processing | Care Coordination and Communication | VI |
| [51] Kaewprag et al. 2015 | Basic | USA | Hospital | Machine Learning | Automated Planning and Scheduling | Pressure ulcers | VI |
| [52] Kalidas & Tamil 2016 | Basic | USA | Hospital | Machine Learning | Image and Signal Processing | Alarms | VI |
| [53] Kao et al. 2018 | Basic | USA | Hospital | Machine Learning | Automated Planning and Scheduling | Quality of Life and Wellbeing of Caregivers | VI |
| [54] Kundu & Acharyya et al. 2008 | Basic | IND | Hospital | Machine Learning | Automated Planning and Scheduling | Nurse Rostering/Scheduling | VI |
| [55] Lea et al. 2013 | Basic | USA | Hospital | Machine Learning | Image and Signal Processing | Care Coordination and Communication | VI |
| [56] Lehman et al. 2018 | Basic | USA | Hospital | Machine Learning | Image and Signal Processing | Alarms | VI |
| [57] Li & Mathews 2017 | Basic | USA | Hospital | Machine Learning | Image and Signal Processing | Pressure ulcers | VI |
| [58] Lo et al. 2007 | Basic | TWN | Hospital | Machine Learning | Automated Planning and Scheduling | Nurse Rostering/Scheduling | VI |
| [59] Luo et al. 2019 | Basic | CHN | Hospital | Machine Learning | Automated Planning and Scheduling | Nursing assessment/care needs assessment | VI |
| [60] Mamun et al. 2014 | Basic | FJI | Hospital | Machine Learning | Image and Signal Processing | Activity and Health | VI |
| [61] Miyawaki et al. 2005 | Basic | JPN | Hospital | Machine Learning | Image and Signal Processing | Care Coordination and Communication | VI |
| [62] Naya et al. 2011 | Basic | JPN | Hospital | Machine Learning | Automated Planning and Scheduling | Care Coordination and Communication | VI |
| [63] Nii et al. 2007 | Basic | JPN | Hospital | Machine Learning | Natural Language Processing | Care Coordination and Communication | VI |
| [64] Nii et al. 2008 | Basic | JPN | Hospital | Machine Learning | Natural Language Processing | Care Coordination and Communication | VI |
| [65] Nii et al. 2013 | Basic | JPN | Hospital | Machine Learning | Natural Language Processing | Care Coordination and Communication | VI |
| [66] Nii et al. 2010 | Basic | JPN | Hospital | Machine Learning | Natural Language Processing | Care Coordination and Communication | VI |
| [67] Nishio et al. 2020 | Basic | JPN | Hospital | Machine Learning | Automated Planning and Scheduling | Care Coordination and Communication | VI |
| [68] Nouira & Trabelsi 2012 | Basic | TUN | Hospital | Machine Learning | Image and Signal Processing | Alarms | VI |
| [69] Park et al. 2020 | Basic | USA | Hospital | Machine Learning | Image and Signal Processing | Digestion | VI |
| [70] Patterson et al. 2019 | Basic | USA | Hospital | Machine Learning | Automated Planning and Scheduling | Falls | IV |
| [71] Scalzo & Hu   2013 | Basic | USA | Hospital | Machine Learning | Image and Signal Processing | Alarms | VI |
| [72] Steins & Walther 2013 | Basic | SWE | Hospital | Machine Learning | Automated Planning and Scheduling | Nursing assessment/care needs assessment | VI |
| [73] Tejaswini et al. 2018 | Basic | IND | Hospital | Machine Learning | Automated Planning and Scheduling | Pain | VI |
| [74] Todorovic & Petrovic 2013 | Basic | BEL | Hospital | Machine Learning | Automated Planning and Scheduling | Nurse Rostering/Scheduling | VI |
| [75] Urashima et al. 2013 | Basic | JPN | Hospital | Machine Learning | Image and Signal Processing | Care Coordination and Communication | VI |
| [76] Vaquerizo & Corchado 2011 | Basic | SPA | Hospital | Machine Learning | Automated Planning and Scheduling | Nurse Rostering/Scheduling | VI |
| [77] Vazquez-Santacruz et al. 2015 | Basic | MEX | Hospital | Machine Learning | Image and Signal Processing | Mobility, other | VI |
| [78] Cetin Yagmur & Sarucan 2019 | Basic | TUR | Hospital | Machine Learning | Automated Planning and Scheduling | Nurse Rostering/Scheduling | VI |
| [79] Yokota et al. 2017 | Basic | JPN | Hospital | Machine Learning | Automated Planning and Scheduling | Falls | VI |
| [80] Yokota & Ohe 2016 | Basic | JPN | Hospital | Machine Learning | Automated Planning and Scheduling | Falls | VI |
| [81] Yousefi et al. 2011 | Basic | USA | Hospital | Machine Learning | Image and Signal Processing | Pressure ulcers | VI |
| [82] Li & Clifford   2012 | Basic | USA | Hospital | Machine Learning | Image and Signal Processing | Alarms | VI |
| [83] Liao et al. 2015 | Basic | TWN | Hospital | Machine Learning | Automated Planning and Scheduling | Nursing assessment/care needs assessment | VI |
| [84] Lodhi et al. 2017 | Basic | USA | Hospital | Machine Learning | Automated Planning and Scheduling | Nursing assessment/care needs assessment | VI |
| [85] Manna et al. 2019 | Basic | IND | Hospital | Machine Learning | Image and Signal Processing | Alarms | VI |
| [86] Marafino et al. 2015 | Basic | USA | Hospital | Machine Learning | Automated Planning and Scheduling | Nursing assessment/care needs assessment | VI |
| [87] Ostojic et al. 2020 | Basic | CHE | Hospital | Machine Learning | Image and Signal Processing | Alarms | VI |
| [88] Park et al. 2020 | Basic | USA | Hospital | Machine Learning | Automated Planning and Scheduling | Infection Control | VI |
| [89] Patrick & Li   2011 | Basic | AUS | Hospital | Machine Learning | Natural Language Processing | Care Coordination and Communication | VI |
| [90] Scalzo et al. 2013 | Basic | USA | Hospital | Machine Learning | Image and Signal Processing | Alarms | VI |
| [91] Sharma et al. 2020 | Basic | USA | Hospital | Machine Learning | Automated Planning and Scheduling | Parenteral or Enteral Nutrition and Fluid Intake | VII |
| [92] Singh et al. 2017 | Basic | IND | Hospital | Machine Learning | Image and Signal Processing | Care Coordination and Communication | concept |
| [93] Yeung et al. 2019 | Basic | USA | Hospital | Machine Learning | Image and Signal Processing | Activity and Health | VI |
| [94] Yu et al. 2019 | Basic | USA | Hospital | Machine Learning | Image and Signal Processing | Respiratory Care/Weaning | VI |
| [95] Yu et al. 2020 | Basic | KOR | Hospital | Machine Learning | Automated Planning and Scheduling | Care Coordination and Communication | VI |
| [96] Hunter et al. 2008 | Basic | GBR | Hospital | Machine Learning | Automated Planning and Scheduling | Nursing assessment/care needs assessment | VI |
| [97] Alam et al. 2019 | Basic | USA | sLTC | Machine Learning | Image and Signal Processing | Activity and Health | VI |
| [98] Aziz et al. 2017 | Basic | CAN | sLTC | Machine Learning | Image and Signal Processing | Falls | VI |
| [99] Cufoglu & Chin 2015 | Basic | GBR | sLTC | Machine Learning | Automated Planning and Scheduling | Nursing assessment/care needs assessment | VI |
| [100] Gannod et al. 2018 | Basic | USA | sLTC | Machine Learning | Automated Planning and Scheduling | Care Coordination and Communication | VI |
| [101] Gargees et al. 2017 | Basic | USA | sLTC | Machine Learning | Image and Signal Processing | Activity and Health | VI |
| [102] Jing et al. 2017 | Basic | GBR | sLTC | Machine Learning | Image and Signal Processing | Activity and Health | concept |
| [103] Khosla & Chu 2013 | Basic | AUS | sLTC | Machine Learning | Image and Signal Processing | Social Integration and Participation | VI |
| [104] Kingetsu et al. 2019 | Basic | JPN | sLTC | Machine Learning | Image and Signal Processing | Falls | VI |
| [105] Kobayashi et al. 2012 | Basic | JPN | sLTC | Machine Learning | Image and Signal Processing | Care Coordination and Communication | VI |
| [106] Krüger et al. 2017 | Basic | GER | sLTC | Machine Learning | Natural Language Processing | Activity and Health | VI |
| [107] Kubota et al. 2008 | Basic | JPN | sLTC | Machine Learning | Image and Signal Processing | Activity and Health | VI |
| [108] Morita et al. 2018 | Basic | JPN | sLTC | Machine Learning | Automated Planning and Scheduling | Care Coordination and Communication | VI |
| [109] Muckell et al. 2017 | Basic | USA | sLTC | Machine Learning | Image and Signal Processing | Activity and Health | VI |
| [110] Swangnetr & Kaber 2013 | Basic | USA | sLTC | Machine Learning | Image and Signal Processing | Activity and Health | VI |
| [111] Toda & Shinomiya 2019 | Basic | JPN | sLTC | Machine Learning | Image and Signal Processing | Falls | VI |
| [112] Volrathongchai 2005 | Basic | USA | sLTC | Machine Learning | Automated Planning and Scheduling | Falls | VI |
| [113] Wu & Zhao 2018 | Basic | USA | sLTC | Machine Learning | Image and Signal Processing | Activity and Health | VI |
| [114] Yamazaki et al. 2016 | Basic | JPN | sLTC | Machine Learning | Image and Signal Processing | Activity and Health | VI |
| [115] Yu et al. 2013 | Basic | USA | sLTC | Machine Learning | Image and Signal Processing | Activity and Health | VI |
| [116] Alam et al. 2016 | Basic | USA | aLTC | Machine Learning | Automated Planning and Scheduling | Activity and Health | VI |
| [117] Albina & Hernandez 2018 | Basic | PHL | aLTC | Machine Learning | Image and Signal Processing | Activity and Health | VI |
| [118] Easton et al. 2019 | Basic | GBR | aLTC | Machine Learning | Natural Language Processing | Care Coordination and Communication | VI |
| [119] Kuspinar et al. 2019 | Basic | CAN | aLTC | Machine Learning | Automated Planning and Scheduling | Falls | VI |
| [120] Mano 2018 | Basic | BRA | aLTC | Machine Learning | Image and Signal Processing | Activity and Health | VI |
| [121] Shen et al. 2013 | Basic | TWN | aLTC | Machine Learning | Image and Signal Processing | Falls | VI |
| [122] Nguyen-Truong & Fritz 2018 | Basic | USA | aLTC | Machine Learning | N/A | Activity and Health | VII |
| [123] Ghose et al. 2015 | Basic | AUS | Community | Machine Learning | Automated Planning and Scheduling | Activity and Health | VI |
| [124] Su & Chiang   2014 | Basic | TWN | Community | Machine Learning | Image and Signal Processing | Care Coordination and Communication | VI |
| [125] Yuan et al. 2017 | Basic | CHN | Community | Machine Learning | Image and Signal Processing | Activity and Health | concept |
| [126] Uronen et al. 2020 | Basic | FIN | Community | Machine Learning | Natural Language Processing | Quality of Life and Wellbeing of Caregivers | IV |
| [127] Vuono et al. 2018 | Basic | ITA | Community | Machine Learning | Image and Signal Processing | Social integration and participation | concept |
| [128] Babič et al. 2016 | Basic | SVK | Independent Living | Machine Learning | Automated Planning and Scheduling | Activity and Health | VI |
| [129] Bakola et al. 2014 | Basic | GRC | Independent Living | Machine Learning | Image and Signal Processing | Activity and Health | concept |
| [130] Becker et al. 2006 | Basic | GRC | Independent Living | Machine Learning | Image and Signal Processing | Activity and Health | concept |
| [131] Bizjak et al. 2018 | Basic | SLO | Independent Living | Machine Learning | Image and Signal Processing | Activity and Health | VI |
| [132] Fan et al. 2019 | Basic | HKG | Independent Living | Machine Learning | Image and Signal Processing | Activity and Health | VI |
| [133] Feng et al. 2014 | Basic | GBR | Independent Living | Machine Learning | Image and Signal Processing | Falls | VI |
| [134] Fleury et al. 2010 | Basic | FRA | Independent Living | Machine Learning | Image and Signal Processing | Activity and Health | VI |
| [135] Ganesh et al. 2019 | Basic | USA | Independent Living | Machine Learning | Image and Signal Processing | Social Integration and Participation | VI |
| [136] Gochoo et al. 2018 | Basic | USA | Independent Living | Machine Learning | Image and Signal Processing | Activity and Health | VI |
| [137] Gómez-Sebastià et al. 2016 | Basic | SPA | Independent Living | Machine Learning | Automated Planning and Scheduling | Distribution of Medication | concept |
| [138] Grgurić et al. 2019 | Basic | CRO | Independent Living | Machine Learning | Image and Signal Processing | Quality of Life and Wellbeing of Caregivers | IV |
| [139] Haigh et al. 2006 | Basic | USA | Independent Living | Machine Learning | Image and Signal Processing | Activity and Health | VI |
| [140] Howcroft et al. 2017 | Basic | CAN | Independent Living | Machine Learning | Image and Signal Processing | Falls | VI |
| [141] Hsu et al. 2009 | Basic | TWN | Independent Living | Machine Learning | Image and Signal Processing | Activity and Health | VI |
| [142] Hu et al. 2020 | Basic | CHN | Independent Living | Machine Learning | Image and Signal Processing | Activity and Health | VI |
| [143] Juang & Wu 2013 | Basic | CHN | Independent Living | Machine Learning | Image and Signal Processing | Activity and Health | VI |
| [144] Juang & Wu 2015 | Basic | CHN | Independent Living | Machine Learning | Image and Signal Processing | Falls | VI |
| [145] Kang et al. 2012 | Basic | KOR | Independent Living | Machine Learning | Image and Signal Processing | Activity and Health | VI |
| [146] Kasteren et al. 2010 | Basic | NLD | Independent Living | Machine Learning | Image and Signal Processing | Activity and Health | VI |
| [147] Lei et al. 2019 | Basic | CHN | Independent Living | Machine Learning | Image and Signal Processing | Activity and Health | VI |
| [148] Niiuchi et al. 2018 | Basic | JPN | Independent Living | Machine Learning | Image and Signal Processing | Activity and Health | VI |
| [149] Nuutinen et al. 2017 | Basic | FIN | Independent Living | Machine Learning | Automated Planning and Scheduling | Nursing assessment/care needs assessment | IV |
| [150] Okano et al. 2013 | Basic | JPN | Independent Living | Machine Learning | Image and Signal Processing | Nursing assessment/care needs assessment | VI |
| [151] Pahl & Varadarajan 2015 | Basic | GER | Independent Living | Machine Learning | Image and Signal Processing | Activity and Health | VI |
| [152] Richter & Hirtz 2013 | Basic | GER | Independent Living | Machine Learning | Image and Signal Processing | Activity and Health | VI |
| [153] Sadreazami et al. 2018 | Basic | CAN | Independent Living | Machine Learning | Image and Signal Processing | Falls | VI |
| [154] Sadreazami et al. 2019 | Basic | CAN | Independent Living | Machine Learning | Image and Signal Processing | Falls | VI |
| [155] Sadreazami et al. 2019 | Basic | CAN | Independent Living | Machine Learning | Image and Signal Processing | Falls | VI |
| [156] Sakakibara et al. 2017 | Basic | JPN | Independent Living | Machine Learning | Natural Language Processing | Care Coordination and Communication | concept |
| [157] Soni & Choudhary 2019 | Basic | IND | Independent Living | Machine Learning | Image and Signal Processing | Falls | VI |
| [158] Suryadevara & Mukhopadhyay 2014 | Basic | IND | Independent Living | Machine Learning | Image and Signal Processing | Activity and Health | VI |
| [159] Suryadevara et al. 2012 | Basic | IND | Independent Living | Machine Learning | Image and Signal Processing | Activity and Health | VI |
| [160] Suzuki et al. 2011 | Basic | JPN | Independent Living | Machine Learning | Image and Signal Processing | Distribution of Medication | VI |
| [161] Tokunaga et al. 2016 | Basic | JPN | Independent Living | Machine Learning | Image and Signal Processing | Care Coordination and Communication | concept |
| [162] Weimin et al. 2010 | Basic | SGP | Independent Living | Machine Learning | Image and Signal Processing | Speech | VI |
| [163] Wong et al. 2017 | Basic | HKG | Independent Living | Machine Learning | Automated Planning and Scheduling | Nursing assessment/care needs assessment | VI |
| [164] Wu et al. 2016 | Basic | USA | Independent Living | Machine Learning | Image and Signal Processing | Activity and Health | VI |
| [165] Yu & Chan 2017 | Basic | HKG | Independent Living | Machine Learning | Image and Signal Processing | Activity and Health | VI |
| [166] Yu et al. 2012 | Basic | CHN | Independent Living | Machine Learning | Image and Signal Processing | Falls | VI |
| [167] Yu et al. 2013 | Basic | CHN | Independent Living | Machine Learning | Image and Signal Processing | Falls | VI |
| [168] Zeng & Chang 2016 | Basic | TWN | Independent Living | Machine Learning | Image and Signal Processing | Activity and Health | VI |
| [169] Zeng et al. 2017 | Basic | TWN | Independent Living | Machine Learning | Image and Signal Processing | Wounds, other | VI |
| [170] Paul et al. 2014 | Basic | USA | Independent Living | Machine Learning | Image and Signal Processing | Activity and Health | VI |
| [171] Veyron et al. 2019 | Basic | FRA | Independent Living | Machine Learning | Automated Planning and Scheduling | Nursing assessment/care needs assessment | IV |
| [172] An et al. 2012 | Basic | USA | Outpatient | Machine Learning | Automated Planning and Scheduling | Activity and Health | concept |
| [173] Chen et al. 2018 | Basic | TWN | Outpatient | Machine Learning | Image and Signal Processing | Wounds, other | VI |
| [174] Cheng et al. 2015 | Basic | CAN | Outpatient | Machine Learning | Automated Planning and Scheduling | Care Coordination and Communication | VI |
| [175] Clark et al. 2014 | Basic | USA | Outpatient | Machine Learning | Natural Language Processing | Speech | VI |
| [176] Freed et al. 2016 | Basic | USA | Outpatient | Machine Learning | Image and Signal Processing | Parenteral or Enteral Nutrition and Fluid Intake | VI |
| [177] Jung et al. 2016 | Basic | USA | Outpatient | Machine Learning | Automated Planning and Scheduling | Wounds, other | VI |
| [178] Ohura et al. 2019 | Basic | JPN | Outpatient | Machine Learning | Image and Signal Processing | Pressure ulcers | VI |
| [179] Stiglic & Kokol 2005 | Basic | SLO | Outpatient | Machine Learning | Automated Planning and Scheduling | Nurse Rostering/Scheduling | VI |
| [180] Li et al. 2018 | Basic | CHN | Rehabilitation | Machine Learning | Image and Signal Processing | Falls | VI |
| [181] Yang et al. 2016 | Basic | CHN | Rehabilitation | Machine Learning | Image and Signal Processing | Mobility, other | VI |
| [182] Ghosh et al. 2019 | Basic | IND | Multiple | Machine Learning | Image and Signal Processing | Activity and Health | VI |
| [183] Inoue et al. 2019 | Basic | JPN | Multiple | Machine Learning | Image and Signal Processing | Care Coordination and Communication | VI |
| [184] Jalal et al. 2014 | Basic | KOR | Multiple | Machine Learning | Image and Signal Processing | Activity and Health | VI |
| [185] Jin et al. 2012 | Basic | KOR | Multiple | Machine Learning | Image and Signal Processing | Activity and Health | concept |
| [186] Khan et al. 2017 | Basic | CAN | Multiple | Machine Learning | Image and Signal Processing | Activity and Health | VI |
| [187] Kumar et al. 2019 | Basic | FRA | Multiple | Machine Learning | Automated Planning and Scheduling | Nurse Rostering/Scheduling | VI |
| [188] Liu et al. 2018 | Basic | TWN | Multiple | Machine Learning | Natural Language Processing | Social Integration and Participation | concept |
| [189] Machanje et al. 2019 | Basic | KEN | Multiple | Machine Learning | Natural Language Processing | Speech | VI |
| [190] Mori et al. 2013 | Basic | JPN | Multiple | Machine Learning | Image and Signal Processing | Activity and Health | VI |
| [191] Nii et al. 2017 | Basic | JPN | Multiple | Machine Learning | Image and Signal Processing | Parenteral or Enteral Nutrition and Fluid Intake | VI |
| [192] Nii et al. 2014 | Basic | JPN | Multiple | Machine Learning | Natural Language Processing | Care Coordination and Communication | VI |
| [193] Nii et al. 2014 | Basic | JPN | Multiple | Machine Learning | Natural Language Processing | Care Coordination and Communication | VI |
| [194] Nii et al. 2015 | Basic | JPN | Multiple | Machine Learning | Natural Language Processing | Care Coordination and Communication | VI |
| [195] Nii et al. 2016 | Basic | JPN | Multiple | Machine Learning | Natural Language Processing | Care Coordination and Communication | VI |
| [196] Ongenae et al. 2012 | Basic | BEL | Multiple | Machine Learning | Image and Signal Processing | Nurse Rostering/Scheduling | VI |
| [197] Pessemier et al. 2013 | Basic | BEL | Multiple | Machine Learning | Automated Planning and Scheduling | Parenteral or Enteral Nutrition and Fluid Intake | concept |
| [198] Phan et al. 2019 | Basic | AUS | Multiple | Machine Learning | Automated Planning and Scheduling | Care Coordination and Communication | VI |
| [199] Ramanujam & Padmavathi 2019 | Basic | IND | Multiple | Machine Learning | Image and Signal Processing | Falls | VI |
| [200] Tian 2018 | Basic | CHN | Multiple | Machine Learning | Automated Planning and Scheduling | Nursing assessment/care needs assessment | VI |
| [201] Di Pietro et al. 2010 | Basic | CAN | Multiple | Machine Learning | Automated Planning and Scheduling | Care Coordination and Communication | VI |
| [202] Wai et al. 2009 | Basic | SGP | Multiple | Machine Learning | Image and Signal Processing | Pressure ulcers | VI |
| [203] Wickramasinghe et al. 2017 | Basic | AUS | Multiple | Machine Learning | Image and Signal Processing | Activity and Health | VI |
| [204] Sinn et al. 2017 | Basic | CAN | Multiple | Machine Learning | Automated Planning and Scheduling | Care Coordination and Communication | VI |
| [205] Batata et al. 2018 | Basic | FRA | not stated | Machine Learning | Automated Planning and Scheduling | Quality of Life and Wellbeing of Caregivers | VI |
| [206] Gomes et al. 2019 | Basic | BRA | not stated | Machine Learning | Natural Language Processing | Care Coordination and Communication | VII |
| [207] Demmer et al. 2017 | Basic | GER | not stated | Machine Learning | Image and Signal Processing | Activity and Health | VI |
| [208] Esfahani et al. 2020 | Basic | USA | not stated | Machine Learning | Image and Signal Processing | Parenteral or Enteral Nutrition and Fluid Intake | VI |
| [209] Fodeh et al. 2018 | Basic | USA | not stated | Machine Learning | Natural Language Processing | Care Coordination and Communication | VI |
| [210] Hsu et al. 2017 | Basic | TWN | not stated | Machine Learning | Image and Signal Processing | Falls | VI |
| [211] Huimin et al. 2008 | Basic | CHN | not stated | Machine Learning | Image and Signal Processing | Falls | VI |
| [212] Johnson 2006 | Basic | USA | not stated | Machine Learning | Automated Planning and Scheduling | Care Coordination and Communication | concept |
| [213] Lee et al. 2017 | Basic | USA | not stated | Machine Learning | Image and Signal Processing | Falls | VI |
| [214] Luo et al. 2018 | Basic | CHN | not stated | Machine Learning | Image and Signal Processing | Activity and Health | VI |
| [215] Luu et al. 2017 | Basic | AUS | not stated | Machine Learning | Automated Planning and Scheduling | Care Coordination and Communication | VI |
| [216] Magyar et al. 2019 | Basic | JPN | not stated | Machine Learning | Natural Language Processing | Speech | VI |
| [217] Masafumi et al. 2010 | Basic | JPN | not stated | Machine Learning | Automated Planning and Scheduling | Nurse Rostering/Scheduling | VI |
| [218] Mihailidis et al. 2007 | Basic | CAN | not stated | Machine Learning | Image and Signal Processing | Activity and Health | VI |
| [219] Mitabe & Shinomiya 2017 | Basic | JPN | not stated | Machine Learning | Natural Language Processing | Activity and Health | VI |
| [220] Mitabe & Shinomiya 2018 | Basic | JPN | not stated | Machine Learning | Image and Signal Processing | Falls | VI |
| [221] Morita et al. 2015 | Basic | JPN | not stated | Machine Learning | Natural Language Processing | Care Coordination and Communication | VI |
| [222] Si et al. 2007 | Basic | JPN | not stated | Machine Learning | Image and Signal Processing | Activity and Health | concept |
| [223] Si et al. 2007 | Basic | JPN | not stated | Machine Learning | Image and Signal Processing | Activity and Health | VI |
| [224] Takadama et al. 2010 | Basic | JPN | not stated | Machine Learning | Image and Signal Processing | Activity and Health | VI |
| [225] Wei et al. 2013 | Basic | CHN | not stated | Machine Learning | Natural Language Processing | Care Coordination and Communication | VI |
| [226] Wiratanaya et al. 2007 | Basic | JPN/USA | not stated | Machine Learning | Image and Signal Processing | Social Integration and Participation | concept |
| [227] Yamada et al. 2017 | Basic | JPN | not stated | Machine Learning | Image and Signal Processing | Parenteral or Enteral Nutrition and Fluid Intake | VI |
| [228] Asif et al. 2020 | Basic | AUS | not stated | Machine Learning | Image and Signal Processing | Falls | VI |
| [229] Cyras et al. 2020 | Basic | GBR | not stated | Machine Learning | Automated Planning and Scheduling | Nurse Rostering/Scheduling | concept |
| [230] Aziz et al. 2016 | Basic | CAN | N/A | Machine Learning | Image and Signal Processing | Falls | VI |
| [231] Belshaw et al. 2011 | Basic | CAN | N/A | Machine Learning | Image and Signal Processing | Falls | VI |
| [232] Samra et al. 2016 | Basic | AUS | N/A | Machine Learning | Image and Signal Processing | Clinical Education | concept |
|  | | | | | | | |
| [233] Adlassnig et al. 2009 | Basic | AUT | Hospital | Expert System | Automated Planning and Scheduling | Infection Control | VI |
| [234] Arif et al. 2017 | Basic | INT | Hospital | Expert System | Image and Signal Processing | Distribution of Medication | concept |
| [235] Baxter et al. 2005 | Basic | GBR | Hospital | Expert System | Image and Signal Processing | Respiratory Care/Weaning | VI |
| [236] Ding et al. 2017 | Basic | TWN | Hospital | Expert System | Automated Planning and Scheduling | Pressure ulcers | VI |
| [237] Fernandes et al. 2019 | Basic | BRA | Hospital | Expert System | Image and Signal Processing | Alarms | VI |
| [238] Jordan & Rose 2010 | Basic | USA | Hospital | Expert System | Image and Signal Processing | Care Coordination and Communication | VI |
| [239] Dingli et al. 2008 | Basic | MLT | Hospital | Expert System | Image and Signal Processing | Activity and Health | concept |
| [240] Tapia & Corchado et al. 2009 | Basic | SPA | sLTC | Expert System | Image and Signal Processing | Activity and Health | VI |
| [241] Tseng et al. 2013 | Basic | CHN | sLTC | Expert System | Image and Signal Processing | Activity and Health | VI |
| [242] Alnosayan et al. 2014 | Basic | USA | aLTC | Expert System | Automated Planning and Scheduling | Care Coordination and Communication | VI |
| [243] Chen et al. 2012 | Basic | TWN | aLTC | Expert System | Image and Signal Processing | Activity and Health | VI |
| [244] Park et al. 2011 | Basic | KOR | Community | Expert System | Image and Signal Processing | Activity and Health | VI |
| [245] Augustyniak 2013 | Basic | POL | Independent Living | Expert System | Image and Signal Processing | Activity and Health | VI |
| [246] Hsu et al. 2009 | Basic | TWN | Independent Living | Expert System | Image and Signal Processing | Activity and Health | VI |
| [247] Lim et al. 2011 | Basic | USA | Independent Living | Expert System | Image and Signal Processing | Care Coordination and Communication | concept |
| [248] Merico et al. 2013 | Basic | ITA | Independent Living | Expert System | Image and Signal Processing | Activity and Health | concept |
| [249] Shieh & Shieh 2013 | Basic | TWN | Independent Living | Expert System | Image and Signal Processing | Activity and Health | VI |
| [250] Tamamizu et al. 2016 | Basic | JPN | Independent Living | Expert System | Image and Signal Processing | Activity and Health | VI |
| [251] Thomas et al. 2013 | Basic | GBR | Independent Living | Expert System | Image and Signal Processing | Activity and Health | concept |
| [252] Wai et al. 2011 | Basic | SGP | Independent Living | Expert System | Image and Signal Processing | Activity and Health | concept |
| [253] Wingrave et al. 2012 | Basic | USA | Independent Living | Expert System | Image and Signal Processing | Quality of Life and Wellbeing of Caregivers | concept |
| [254] Luperto et al. 2018 | Basic | ITA | Independent Living | Expert System | Natural Language Processing | Nursing assessment/care needs assessment | concept |
| [255] Chouvarda et al. 2014 | Basic | GBR | Outpatient | Expert System | Image and Signal Processing | COPD care | concept |
| [256] Nie et al. 2019 | Basic | USA | Outpatient | Expert System | Image and Signal Processing | Social Integration and Participation | VI |
| [257] Bleda et al. 2018 | Basic | SPA | Multiple | Expert System | Image and Signal Processing | Activity and Health | VI |
| [258] Nii et al. 2009 | Basic | JPN | Multiple | Expert System | Natural Language Processing | Care Coordination and Communication | VI |
| [259] Vairaktarakis et al. 2015 | Basic | GRC | Multiple | Expert System | Automated Planning and Scheduling | Nursing assessment/care needs assessment | concept |
| [260] Jung & Park   2019 | Basic | KOR | not stated | Expert System | Image and Signal Processing | Falls | VI |
| [261] Yang et al. 2013 | Basic | KOR | not stated | Expert System | Image and Signal Processing | Nursing assessment/care needs assessment | VI |
|  | | | | | | | |
| [262] Lu et al. 2006 | Basic | USA | Hospital | Hybrid System | Automated Planning and Scheduling | Activity and Health | VI |
| [263] Bouchard et al. 2008 | Basic | CAN | aLTC | Hybrid System | Image and Signal Processing | Nursing assessment/care needs assessment | VI |
| [264] Zhou et al. 2011 | Basic | SGP | Independent Living | Hybrid System | Image and Signal Processing | Activity and Health | VI |
| [265] Luštrek et al. 2012 | Basic | SLO | Independent Living | Hybrid System | Image and Signal Processing | Falls | VI |
| [266] Yang et al. 2017 | Basic | TWN | not stated | Hybrid System | Image and Signal Processing | Care Coordination and Communication | VI |
|  | | | | | | | |
| [267] Sadahiro et al. 2007 | Basic | JPN | Hospital | not specified | Automated Planning and Scheduling | Care Coordination and Communication | VI |
| [268] Robben et al. 2015 | Basic | NLD | aLTC | not specified | Automated Planning and Scheduling | Care Coordination and Communication | VI |
| [269] Cesta et al. 2005 | Basic | ITA | Independent Living | not specified | Image and Signal Processing | Social Integration and Participation | concept |
| [270] Pascual et al. 2008 | Basic | SPA | Independent Living | not specified | Image and Signal Processing | Activity and Health | concept |
| [271] Wang et al. 2010 | Basic | SGP | Independent Living | not specified | Image and Signal Processing | Falls | VI |
| [272] Weisenberg et al. 2008 | Basic | USA | Independent Living | not specified | Image and Signal Processing | Activity and Health | VI |
| [273] Xu et al. 2016 | Basic | CHN | Independent Living | not specified | Image and Signal Processing | Falls | VI |
| [274] Zejda et al. 2010 | Basic | CZE | Independent Living | not specified | Image and Signal Processing | Social Integration and Participation | concept |
| [275] Annicchiarico et al. 2008 | Basic | SPA | Independent Living | not specified | Image and Signal Processing | Falls | concept |
| [276] Bono et al. 2007 | Basic | JPN | not stated | not specified | Image and Signal Processing | Activity and Health | VI |
| [277] Westra et al. 2016 | Basic | not specified | not stated | not specified | Automated Planning and Scheduling | Care Coordination and Communication | VII |
|  | | | | | | | |
| [278] O'Brien et al. 2020 | Basic | USA | Independent Living | N/A | N/A | Speech | VI |
|  | | | | | | | |
| [279] Shillan et al. 2019 | Review | GBR | Hospital | Machine Learning | N/A | N/A | V |
| [280] Al-Shaqi et al. 2016 | Review | GBR | Independent Living | N/A | N/A | Activity and Health | V |
| [281] Campos et al. 2016 | Review | MEX | Independent Living | not specified | N/A | Social Integration and Participation | V |
| [282] Krishnan et al. 2014 | Review | INT | Multiple | Machine Learning | Image and Signal Processing | Mobility, other | V |
| [283] Rouleau et al. 2017 | Review | CAN | Multiple | N/A | N/A | N/A | V |
| [284] Cresswell et al. 2020 | Review | INT | not stated | Machine Learning | N/A | N/A | I |
| [285] Kahn et al. 2018 | Review | CAN | not stated | Machine Learning | Image and Signal Processing | Activity and Health | V |
| [286] Zahia et al. 2020 | Review | SPA, USA | not stated | Machine Learning | Image and Signal Processing | Pressure ulcers | V |
|  | | | | | | | |
| [287] Gerke et al. 2020 | ELSI | USA | Hospital | Machine Learning | Image and Signal Processing | Activity and Health | VII |
| [288] Wangmo et al. 2019 | ELSI | CHE | Multiple | Machine Learning | N/A | N/A | VI |
| [289] Frize et al. 2005 | ELSI | CAN | Hospital | Expert System | Automated Planning and Scheduling | Care Coordination and Communication | VI |
| [290] Portacolone et al. 2020 | ELSI | USA | Multiple | N/A | N/A | N/A | VI |
| [291] McCradden et al. 2020 | ELSI | CAN | Community | N/A | N/A | N/A | VI |
| [292] Peirce et al. 2019 | ELSI | USA | Various | N/A | N/A | N/A | VII |

**References**

1. Alderden J, Pepper GA, Wilson A, Whitney JD, Richardson S, Butcher R, et al. Predicting Pressure Injury in Critical Care Patients: A Machine-Learning Model. American journal of critical care : an official publication, American Association of Critical-Care Nurses. 2018 Nov;27(6):461-8. PMID: 30385537.

2. Antoniadi AM, Galvin M, Heverin M, Hardiman O, Mooney C. Prediction of caregiver burden in amyotrophic lateral sclerosis: a machine learning approach using random forests applied to a cohort study. BMJ open. 2020 Feb-28;10(2):e033109. PMID: 32114464.

3. Brom H, Brooks Carthon JM, Ikeaba U, Chittams J. Leveraging Electronic Health Records and Machine Learning to Tailor Nursing Care for Patients at High Risk for Readmissions. Journal of nursing care quality. 2020 Jan-Mar;35(1):27-33. PMID: 31136529.

4. Cho I, Park I, Kim E, Lee E, Bates DW. Using EHR data to predict hospital-acquired pressure ulcers: a prospective study of a Bayesian Network model. International journal of medical informatics. 2013 Nov;82(11):1059-67. PMID: 23891086.

5. Davoudi A, Malhotra KR, Shickel B, Siegel S, Williams S, Ruppert M, et al. Intelligent ICU for Autonomous Patient Monitoring Using Pervasive Sensing and Deep Learning. Scientific reports. 2019 May-29;9(1):8020. PMID: 31142754.

6. Marschollek M, Wolf K, Gietzelt M, Nemitz G, Schwabedissen HMz, Haux R, editors. Assessing elderly persons' fall risk using spectral analysis on accelerometric data - a clinical evaluation study. 2008 20-25-Aug.

7. Papageorgiou XS, Chalvatzaki G, Dometios A, Tzafestas CS, Maragos P, editors. Intelligent Assistive Robotic Systems for the elderly: Two real-life use cases. 2017 2017: ACM.

8. Tawfik DS, Profit J, Lake ET, Liu JB, ers LM, Phibbs CS. Development and use of an adjusted nurse staffing metric in the neonatal intensive care unit. Health services research. 2020 Apr;55(2):190-200. PMID: 31869865.

9. Ye C, Li J, Hao S, Liu M, Jin H, Zheng L, et al. Identification of elders at higher risk for fall with statewide electronic health records and a machine learning algorithm. International journal of medical informatics. 2020 2020;137:N.PAG-N.PAG. PMID: 142476870. Language: English. Entry Date: In Process. Revision Date: 20200405. Publication Type: journal article. Journal Subset: Biomedical.

10. Zhang Y. Real-time development of patient-specific alarm algorithms for critical care. Conference proceedings : Annual International Conference of the IEEE Engineering in Medicine and Biology Society IEEE Engineering in Medicine and Biology Society Annual Conference. 2007 2007;2007:4351-4.

11. Zampieri FG, Salluh JIF, Azevedo LCP, Kahn JM, Damiani LP, Borges LP, et al. ICU staffing feature phenotypes and their relationship with patients' outcomes: an unsupervised machine learning analysis. Intensive care medicine. 2019 Nov;45(11):1599-607. PMID: 31595349.

12. Ambagtsheer RC, Shafiabady N, Dent E, Seiboth C, Beilby J. The application of artificial intelligence (AI) techniques to identify frailty within a residential aged care administrative data set. International journal of medical informatics. 2020 Apr;136:104094. PMID: 32058264. doi: 10.1016/j.ijmedinf.2020.104094.

13. Chen D, Bharucha AJ, Wactlar HD, editors. Intelligent Video Monitoring to Improve Safety of Older Persons. 2007 22-26-Aug.

14. Tang V, Siu PKY, Choy KL, Lam HY, Ho GTS, Lee CKM, et al. An adaptive clinical decision support system for serving the elderly with chronic diseases in healthcare industry. Expert Systems. 2019 2019;36(2). doi: 10.1111/exsy.12369.

15. Wai AAP, Fook FS, Jayach, ran M, Biswas J, Lee J-E, et al., editors. Implementation of Context-Aware Distributed Sensor Network System for Managing Incontinence Among Patients with Dementia. 2010 2010: IEEE.

16. Xiong GL, Bayen E, Nickels S, Subramaniam R, Agrawal P, Jacquemot J, et al. Real-time video detection of falls in dementia care facility and reduced emergency care. The American journal of managed care. 2019 Jul;25(7):314-5.

17. Bajo J, Corchardo JM, Rodriguez S. GR-MAS: Multi-Agent System for Geriatric Residences. In: Ghallab M, Spyropoulos CD, Fakotakis N, Avouris N, editors. ECAI 18th European Conference on Artificial Intelligence; Patras, Greece2008.

18. Ala-Kitula A, Talvitie-Lamberg K, Tyrväinen P, Silvennoinen M, editors. Developing Solutions for Healthcare — Deploying Artificial Intelligence to an Evolving Target. International Conference on Computational Science and Computational Intelligence; 2017; Las Vegas, USA.

19. Zhou X, Xu J, Zhao Y. Machine learning methods for anticipating the psychological distress in patients with Alzheimer's disease. Australasian physical & engineering sciences in medicine. 2006 Dec;29(4):303-9.

20. Zhu M, Chen W, Hirdes JP, Stolee P. The K-nearest neighbor algorithm predicted rehabilitation potential better than current Clinical Assessment Protocol. Journal of clinical epidemiology. 2007 Oct;60(10):1015-21. PMID: 17884595.

21. Zhu M, Zhang Z, Hirdes JP, Stolee P. Using machine learning algorithms to guide rehabilitation planning for home care clients. BMC medical informatics and decision making. 2007 Dec-20;7:41. PMID: 18096079.

22. Matsuyama Y, Taniyama H, Fujie S, Kobayashi T. System design of group communication activator: an entertainment task for elderly care. 2009; La Jolla, California, USA: Association for Computing Machinery; 2009. p. 243–4.

23. Yang Y, Hirdes JP, Dubin JA, Lee J. Fall Risk Classification in Community-Dwelling Older Adults Using a Smart Wrist-Worn Device and the Resident Assessment Instrument-Home Care: Prospective Observational Study. JMIR aging. 2019 Jun 7;2(1):e12153. PMID: 31518278. doi: 10.2196/12153.

24. Yamamoto K, Yoshii M, Kinoshita F, Touyama H, editors. Classification vs Regression by CNN for Handwashing Skills Evaluations in Nursing Education. 2020 19-21-Feb.

25. Evans RS, Kuttler KG, Simpson KJ, Howe S, Crossno PF, Johnson KV, et al. Automated detection of physiologic deterioration in hospitalized patients. Journal of the American Medical Informatics Association : JAMIA. 2015 Mar;22(2):350-60. doi: 10.1136/amiajnl-2014-002816.

26. Tapia DI, Rodr\iguez S, Corchado JM. A Distributed Ambient Intelligence Based Multi-Agent System for Alzheimer Health Care. Springer; 2010. p. 181--99.

27. Alwan M. Passive in-home health and wellness monitoring: overview, value and examples. Conference proceedings : Annual International Conference of the IEEE Engineering in Medicine and Biology Society IEEE Engineering in Medicine and Biology Society Annual Conference. 2009 2009;2009:4307-10.

28. Hirdes JP, Poss JW, Curtin-Telegdi N. The Method for Assigning Priority Levels (MAPLe): a new decision-support system for allocating home care resources. BMC medicine. 2008 Mar 26;6:9. PMID: 18366782. doi: 10.1186/1741-7015-6-9.

29. Abdullah R, Fakieh B. Health Care Employees' Perceptions of the Use of Artificial Intelligence Applications: Survey Study. Journal of medical Internet research. 2020 May 14;22(5):e17620. doi: 10.2196/17620.

30. Amato F, Bianchi S, Comai S, Crovari P, Pasquarelli MGG, Imtiaz A, et al., editors. CLONE: a Promising System for the Remote Monitoring of Alzheimer’s Patients: An Experimentation with a Wearable Device in a Village for Alzheimer’s Care. Goodtechs '18: Proceedings of the 4th EAI International Conference on Smart Objects and Technologies for Social Good; 2018 2018; Bologna, Italy: Association for Computing Machinery.

31. Bickmore TW, Pfeifer LM, Jack BW. Taking the time to care. Proceedings of the 27th international conference on Human factors in computing systems - CHI 09; 2009; Boston, MA, USA: Association for Computing Machinery; 2009.

32. Carros F, Meurer J, Löffler D, Unbehaun D, Matthies S, Koch I, et al. Exploring Human-Robot Interaction with the Elderly. Proceedings of the 2020 CHI Conference on Human Factors in Computing Systems; 2020; Honolulu, HI, USA: Association for Computing Machinery; 2020. p. 1-12.

33. Mervin MC, Moyle W, Jones C, Murfield J, Draper B, Beattie E, et al. The Cost-Effectiveness of Using PARO, a Therapeutic Robotic Seal, to Reduce Agitation and Medication Use in Dementia: Findings from a Cluster-Randomized Controlled Trial. Journal of the American Medical Directors Association. 2018 Jul;19(7):619-22 e1. PMID: 29325922. doi: 10.1016/j.jamda.2017.10.008.

34. Gonzalez-Usach R, Yacchirema DC, Collado V, Palau CE, Fortino G, Palau CE, et al., editors. AmI Open Source System for the Intelligent Control of Residences for the Elderly. 2017 2017: Springer.

35. Viswanathan P, Little JJ, Mackworth AK, Mihailidis A, editors. An Intelligent Powered Wheelchair for Users with Dementia: Case Studies with NOAH (Navigation and Obstacle Avoidance Help). 2012 2012: AAAI.

36. Abe A, Ozaku HI, Kuwahara N, Kogure K, editors. Cooperation Between Abductive and Inductive Nursing Risk Management. Sixth IEEE International Conference on Data Mining - Workshops (ICDMW'O6); 2006 18-22-Dec.

37. Annangi P, Frigstad S, Subin SB, Torp A, Ramasubramaniam S, Varna S, editors. An automated bladder volume measurement algorithm by pixel classification using random forests. 2016 16-20-Aug.

38. Antink CH, Leonhardt S, Walter M. Reducing false alarms in the ICU by quantifying self-similarity of multimodal biosignals. Physiological measurement. 2016 Aug;37(8):1233-52. PMID: 27454256. doi: 10.1088/0967-3334/37/8/1233.

39. Au-Yeung WM, Sahani AK, Isselbacher EM, Armoundas AA. Reduction of false alarms in the intensive care unit using an optimized machine learning based approach. NPJ digital medicine. 2019 2019;2:86. PMID: 31508497. doi: 10.1038/s41746-019-0160-7.

40. Celi LA, Hinske LC, Alterovitz G, Szolovits P. An artificial intelligence tool to predict fluid requirement in the intensive care unit: a proof-of-concept study. Critical care (London, England). 2008 2008;12(6):R151. PMID: 19046450. doi: 10.1186/cc7140.

41. Cismondi F, Fialho AS, Xiaoning L, Vieira SM, Gray JE, Reti SR, et al., editors. ANN validation system for ICU neonatal data. WCCI 2012 IEEE World Congress on Computational Intelligence June, 10-15, 2012; 2012 10-15-June; Brisbane, Australia.

42. Clavelle JT, Sweeney CD, Swartwout E, Lefton C, Guney S. Leveraging Technology to Sustain Extraordinary Care: A Qualitative Analysis of Meaningful Nurse Recognition. The Journal of nursing administration. 2019 Jun;49(6):303-9. PMID: 31135637. doi: 10.1097/NNA.0000000000000757.

43. Cramer EM, Seneviratne MG, Sharifi H, Ozturk A, Hernandez-Boussard T. Predicting the Incidence of Pressure Ulcers in the Intensive Care Unit Using Machine Learning. EGEMS (Washington, DC). 2019 Sep 5;7(1):49. PMID: 31534981. doi: 10.5334/egems.307.

44. Crump C, Saxena S, Wilson B, Farrell P, Rafiq A, Silvers CT. Using Bayesian networks and rule-based trending to predict patient status in the intensive care unit. AMIA Annual Symposium proceedings AMIA Symposium. 2009 Nov-14;2009:124-8.

45. Durieux BN, Gramling CJ, Manukyan V, Eppstein MJ, Rizzo DM, Ross LM, et al. Identifying Connectional Silence in Palliative Care Consultations: A Tandem Machine-Learning and Human Coding Method. Journal of palliative medicine. 2018 Dec;21(12):1755-60. PMID: 30328760. doi: 10.1089/jpm.2018.0270.

46. Eerikainen LM, Vanschoren J, Rooijakkers MJ, Vullings R, Aarts RM. Reduction of false arrhythmia alarms using signal selection and machine learning. Physiological measurement. 2016 Aug;37(8):1204-16. PMID: 27454128. doi: 10.1088/0967-3334/37/8/1204.

47. Feng T, Narayanan SS, editors. Modeling Behavioral Consistency in Large-Scale Wearable Recordings of Human Bio-Behavioral Signals. ICASSP 2020 - 2020 IEEE International Conference on Acoustics, Speech and Signal Processing (ICASSP); 2020 4-8-May.

48. Gerka A, Pfingsthorn M, Lupkes C, Sparenberg K, Frenken M, Lins C, et al., editors. Detecting the Number of Persons in the Bed Area to Enhance the Safety of Artificially Ventilated Persons. IEEE 20th International Conference on e-Health Networking, Applications and Services; 2018 20.09.2020.

49. Hever G, Cohen L, O'Connor MF, Matot I, Lerner B, Bitan Y. Machine learning applied to multi-sensor information to reduce false alarm rate in the ICU. Journal of clinical monitoring and computing. 2020 Apr;34(2):339-52. PMID: 30955160. doi: 10.1007/s10877-019-00307-x.

50. Hiissa M, Pahikkala T, Suominen H, Lehtikunnas T, Back B, Karsten H, et al. Towards automated classification of intensive care nursing narratives. International journal of medical informatics. 2007 Dec;76 Suppl 3:S362-8. PMID: 17513166. doi: 10.1016/j.ijmedinf.2007.03.003.

51. Kaewprag P, Newton C, Vermillion B, Hyun S, Huang K, Machiraju R. Predictive Modeling for Pressure Ulcers from Intensive Care Unit Electronic Health Records. AMIA Joint Summits on Translational Science proceedings AMIA Joint Summits on Translational Science. 2015 2015;2015:82-6. PMID: 26306245.

52. Kalidas V, Tamil LS. Cardiac arrhythmia classification using multi-modal signal analysis. Physiological measurement. 2016 Aug;37(8):1253-72. PMID: 27454417. doi: 10.1088/0967-3334/37/8/1253.

53. Kao H, Hosseinmardi H, Yan S, Hasan M, Narayanan S, Lerman K, et al., editors. Discovering Latent Psychological Structures from Self-Report Assessments of Hospital Workers. 2018 12-14-Nov.

54. Kundu S, Acharyya S, editors. A SAT approach for solving the nurse scheduling problem. TENCON 2008 - 2008 IEEE Region 10 Conference; 2008 19-21-Nov.; Hyderabad, India.

55. Lea C, Facker J, Hager G, Taylor R, Saria S. 3D Sensing Algorithms Towards Building an Intelligent Intensive Care Unit. AMIA Joint Summits on Translational Science proceedings AMIA Joint Summits on Translational Science. 2013 2013;2013:136-40. PMID: 24303253.

56. Lehman EP, Krishnan RG, Zhao X, Mark RG, Lehman LH. Representation Learning Approaches to Detect False Arrhythmia Alarms from ECG Dynamics. Proceedings of machine learning research. 2018 Aug;85:571-86. PMID: 31723938.

57. Li D, Mathews C. Automated measurement of pressure injury through image processing. Journal of clinical nursing. 2017 Nov;26(21-22):3564-75. PMID: 28071843. doi: 10.1111/jocn.13726.

58. Lo C, Lin C, Wang C, Dai T, Wong D, editors. Artificial immune systems for intelligent nurse rostering. 2007 IEEE International Conference on Industrial Engineering and Engineering Management; 2007 2-4-Dec.; Singapore.

59. Luo L, Li J, Liu C, Shen W. Using machine-learning methods to support health-care professionals in making admission decisions. International Journal of Health Planning & Management. 2019 2019;34(2):e1236-e46. doi: 10.1002/hpm.2769.

60. Mamun KA, Sharma A, Hoque ASM, Szecsi T, editors. Remote patient physical condition monitoring service module for iWARD hospital robots. Asia-Pacific World Congress on Computer Science and Engineering; 2014 4-5-Nov.; Nadi, Fiji

61. Miyawaki F, Masamune K, Suzuki S, Yoshimitsu K, Vain J. Scrub Nurse Robot System—Intraoperative Motion Analysis of a Scrub Nurse and Timed-Automata-Based Model for Surgery. IEEE Transactions on Industrial Electronics. 2005 2005;52(5):1227-35. doi: 10.1109/tie.2005.855692.

62. Naya F, Ohmura R, Miyamae M, Noma H, Kogure K, Imai M. Wireless sensor network system for supporting nursing context-awareness. International Journal of Autonomous and Adaptive Communications Systems. 2011 2011;4(4):361--82. doi: 10.1504/ijaacs.2011.043477.

63. Nii M, Ando S, Takahashi Y, Uchinuno A, Sakashita R, editors. Nursing-Care Freestyle Text Classification Using Support Vector Machines. 2007 IEEE International Conference on Granular Computing (GRC 2007); 2007 2-4-Nov.; Fremont, CA, USA.

64. Nii M, Ando S, Takahashi Y, Uchinuno A, Sakashita R, editors. Feature extraction from nursing-care texts for classification. 2008 World Automation Congress; 2008 28-Sept.-2; Waikoloa, HI, USA.

65. Nii M, Miyake S, Takahama K, Uchinuno A, Sakashita R, editors. Consideration about Utilizing Text Architecture for Making Feature Vectors in Classifying Nursing-Care Texts. 2013 IEEE International Conference on Systems, Man, and Cybernetics; 2013 13-16-Oct.; Manchester, UK.

66. Nii M, Yamaguchi T, Takahashi Y, Sakashita R, Uchinuno A, editors. Analysis of nursing-care freestyle japanese text classification using ga-based term selection. 2010 World Automation Congress; 2010 19-23-Sept.; Kobe, Japan

67. Nishio S, Hossain B, Yagi N, Nii M, Hiranaka T, Kobashi S, editors. Surgical Phase Recognition Method with a Sequential Consistency for CAOS-AI Navigation System. 2020 IEEE 2nd Global Conference on Life Sciences and Technologies (LifeTech 2020); 2020 10-12-March; Kyoto, Japan.

68. Nouira K, Trabelsi A. Intelligent monitoring system for intensive care units. Journal of medical systems. 2012 Aug;36(4):2309-18. PMID: 21505862. doi: 10.1007/s10916-011-9698-x.

69. Park SM, Won DD, Lee BJ, Escobedo D, Esteva A, Aalipour A, et al. A mountable toilet system for personalized health monitoring via the analysis of excreta. Nature biomedical engineering. 2020 Jun;4(6):624-35. PMID: 32251391. doi: 10.1038/s41551-020-0534-9.

70. Patterson BW, Engstrom CJ, Sah V, Smith MA, Mendonca EA, Pulia MS, et al. Training and Interpreting Machine Learning Algorithms to Evaluate Fall Risk After Emergency Department Visits. Medical care. 2019 Jul;57(7):560-6. PMID: 31157707. doi: 10.1097/MLR.0000000000001140.

71. Scalzo F, Hu X. Semi-supervised detection of intracranial pressure alarms using waveform dynamics. Physiological measurement. 2013 Apr;34(4):465-78. PMID: 23524637. doi: 10.1088/0967-3334/34/4/465.

72. Steins K, Walther SM. A generic simulation model for planning critical care resource requirements. Anaesthesia. 2013 Nov;68(11):1148-55. PMID: 24032602. doi: 10.1111/anae.12408.

73. Tejaswini S, Sriraam N, Pradeep GCM, editors. Cloud-Based Framework for Pain Scale Assessment in NICU- A Primitive Study with Infant Cries. 2018, IEEE Third International Conference on Circuits, Control, Communication and Computing; 2018 3-5-Oct.; Bangalore, India

74. Todorovic N, Petrovic S. Bee Colony Optimization Algorithm for Nurse Rostering. IEEE Transactions on Systems, Man, and Cybernetics: Systems. 2013 2013;43(2):467-73. doi: 10.1109/TSMCA.2012.2210404.

75. Urashima A, Nakamura M, Toriyama T, Oshima J, Nakagawa M, Nomura T. Preliminary results of pointing and calling detection system for nurses. Proceedings of the 11th Asia Pacific Conference on Computer Human Interaction - APCHI '13; 2013; Bangalore, India: Association for Computing Machinery; 2013. p. 192-4.

76. Vaquerizo MB, Á H, Corchado E, editors. Visual analysis of nurse rostering solutions through a bio-inspired intelligent model. 2011 11th International Conference on Intelligent Systems Design and Applications; 2011 22-24-Nov.; Cordoba, Spain

77. Vázquez-Santacruz E, Cruz-Santos W, Gamboa-Zúñiga M. Design and Implementation of an Intelligent System for Controlling a Robotic Hospital Bed for Patient Care Assistance. Computación y Sistemas. 2015 2015;19(3). doi: 10.13053/cys-19-3-2013.

78. Cetin Yagmur E, Sarucan A. Nurse Scheduling with Opposition-Based Parallel Harmony Search Algorithm. Journal of Intelligent Systems. 2019 2019;28(4):633-47. doi: 10.1515/jisys-2017-0150.

79. Yokota S, Endo M, Ohe K. Establishing a Classification System for High Fall-Risk Among Inpatients Using Support Vector Machines. Computers, informatics, nursing : CIN. 2017 Aug;35(8):408-16. PMID: 28800580. doi: 10.1097/CIN.0000000000000332.

80. Yokota S, Ohe K. Construction and evaluation of FiND, a fall risk prediction model of inpatients from nursing data. Japan journal of nursing science : JJNS. 2016 Apr;13(2):247-55. PMID: 27040735. doi: 10.1111/jjns.12103.

81. Yousefi R, Ostadabbas S, Faezipour M, Nourani M, Ng V, Tamil L, et al., editors. A smart bed platform for monitoring & Ulcer prevention. 2011 4th International Conference on Biomedical Engineering and Informatics (BMEI); 2011 15-17-Oct.; Shanghai, China

82. Li Q, Clifford GD. Signal quality and data fusion for false alarm reduction in the intensive care unit. Journal of electrocardiology. 2012 Nov-Dec;45(6):596-603. PMID: 22960167. doi: 10.1016/j.jelectrocard.2012.07.015.

83. Liao PH, Hsu PT, Chu W, Chu WC. Applying artificial intelligence technology to support decision-making in nursing: A case study in Taiwan. Health informatics journal. 2015 Jun;21(2):137-48. PMID: 26021669. doi: 10.1177/1460458213509806.

84. Lodhi MK, Ansari R, Yao Y, Keenan GM, Wilkie D, Khokhar AA. Predicting Hospital Re-admissions from Nursing Care Data of Hospitalized Patients. Advances in data mining Industrial Conference on Data Mining. 2017 2017;2017:181-93. PMID: 29104962. doi: 10.1007/978-3-319-62701-4_14.

85. Manna T, Swetapadma A, Abdar M. Decision Tree Predictive Learner-Based Approach for False Alarm Detection in ICU. Journal of medical systems. 2019 May 21;43(7):191. PMID: 31115734. doi: 10.1007/s10916-019-1337-y.

86. Marafino BJ, Boscardin WJ, Dudley RA. Efficient and sparse feature selection for biomedical text classification via the elastic net: Application to ICU risk stratification from nursing notes. Journal of biomedical informatics. 2015 Apr;54:114-20. PMID: 25700665. doi: 10.1016/j.jbi.2015.02.003.

87. Ostojic D, Guglielmini S, Moser V, Fauchere JC, Bucher HU, Bassler D, et al. Reducing False Alarm Rates in Neonatal Intensive Care: A New Machine Learning Approach. Advances in experimental medicine and biology. 2020 2020;1232:285-90. PMID: 31893422. doi: 10.1007/978-3-030-34461-0_36.

88. Park JI, Bliss DZ, Chi CL, Delaney CW, Westra BL. Knowledge Discovery With Machine Learning for Hospital-Acquired Catheter-Associated Urinary Tract Infections. Computers, informatics, nursing : CIN. 2020 Jan;38(1):28-35. PMID: 31524687. doi: 10.1097/CIN.0000000000000562.

89. Patrick J, Li M. An ontology for clinical questions about the contents of patient notes. Journal of biomedical informatics. 2012 Apr;45(2):292-306. PMID: 22142949. doi: 10.1016/j.jbi.2011.11.008.

90. Scalzo F, Liebeskind D, Hu X. Reducing false intracranial pressure alarms using morphological waveform features. IEEE transactions on bio-medical engineering. 2013 Jan;60(1):235-9. PMID: 22851230. doi: 10.1109/TBME.2012.2210042.

91. Sharma V, Sharma V, Khan A, Wassmer DJ, Schoenholtz MD, Hontecillas R, et al. Malnutrition, Health and the Role of Machine Learning in Clinical Setting. Frontiers in nutrition. 2020 2020;7:44. PMID: 32351968. doi: 10.3389/fnut.2020.00044.

92. Singh H, Yadav G, Mallaiah R, Joshi P, Joshi V, Kaur R, et al. iNICU - Integrated Neonatal Care Unit: Capturing Neonatal Journey in an Intelligent Data Way. Journal of medical systems. 2017 Aug;41(8):132. PMID: 28748430. doi: 10.1007/s10916-017-0774-8.

93. Yeung S, Rinaldo F, Jopling J, Liu B, Mehra R, Downing NL, et al. A computer vision system for deep learning-based detection of patient mobilization activities in the ICU. NPJ digital medicine. 2019 2019;2:11. PMID: 31304360. doi: 10.1038/s41746-019-0087-z.

94. Yu C, Liu J, Zhao H. Inverse reinforcement learning for intelligent mechanical ventilation and sedative dosing in intensive care units. BMC medical informatics and decision making. 2019 Apr 9;19(Suppl 2):57. PMID: 30961594. doi: 10.1186/s12911-019-0763-6.

95. Yu JY, Jeong GY, Jeong OS, Chang DK, Cha WC. Machine Learning and Initial Nursing Assessment-Based Triage System for Emergency Department. Healthcare informatics research. 2020 Jan;26(1):13-9. PMID: 32082696. doi: 10.4258/hir.2020.26.1.13.

96. Hunter J, Gatt A, Portet F, Reiter E, Sripada S, editors. Using Natural Language Generation Technology to Improve Information Flows in Intensive Care Units. ECAI 2008; 2008.

97. Alam MAU, Heching A, Palmarini N, editors. Scaling Longitudinal Functional Health Assessment in Multi-Inhabitant Smarthome. 019 IEEE 39th International Conference on Distributed Computing Systems (ICDCS); 2019 7-10-July; Dallas, TX, USA.

98. Aziz O, Klenk J, Schwickert L, Chiari L, Becker C, Park EJ, et al. Validation of accuracy of SVM-based fall detection system using real-world fall and non-fall datasets. PloS one. 2017 2017;12(7):e0180318. PMID: 28678808. doi: 10.1371/journal.pone.0180318.

99. Cufoglu A, Chin J, editors. Towards an understanding classification of well-being for care of older people. 2015 IEEE 13th International Conference on Industrial Informatics (INDIN); 2015 22-24-July; Cambridge, UK

100. Gannod GC, Abbott KM, Haitsma KV, Martindale N, Jennings RA, Long CN, et al., editors. Using Machine Learning to Facilitate the Delivery of Person Centered Care in Nursing Homes. Proceedings of the Thirty-First International Florida Artificial Intelligence Research Society Conference, FLAIRS 2018; 2018 May 21-23; Melbourne, Florida, USA: AAAI Press.

101. Gargees R, Keller J, Popescu M, editors. Early illness recognition in older adults using transfer learning. 017 IEEE International Conference on Bioinformatics and Biomedicine (BIBM); 2017 13-16-Nov.; Kansas City, MO, USA

102. Jing Y, Eastwood M, Tan B, Konios A, Hamid A, Collinson M. An intelligent well-being monitoring system for residents in extra care homes. Proceedings of the 1st International Conference on Internet of Things and Machine Learning; 2017; Liverpool, United Kingdom: Association for Computing Machinery; 2017. p. 1-6.

103. Khosla R, Chu M-T. Embodying Care in Matilda. ACM Transactions on Management Information Systems. 2013 2013;4(4):1-33. doi: 10.1145/2544104.

104. Kingetsu H, Konno T, Awai S, Fukuda D, Sonoda T, editors. Video-based Fall Risk Detection System for the Elderly. 2019 IEEE 1st Global Conference on Life Sciences and Technologies (LifeTech 2019); 2019 12-14-March; Osaka, Japan.

105. Kobayashi Y, Yamazaki K, Yamazaki A, Gyoda M, Tabata T, Kuno Y, et al. Care robot able to show the order of service provision through bodily actions in multi-party settings. Proceedings of the 2012 ACM annual conference extended abstracts on Human Factors in Computing Systems Extended Abstracts - CHI EA '12; 2012; Austin, Texas, USA: Association for Computing Machinery; 2012.

106. Krüger F, Heine C, Bader S, Hein A, Teipel S, Kirste T, editors. On the applicability of clinical observation tools for human activity annotation. 2017 IEEE International Conference on Pervasive Computing and Communications Workshops (PerCom Workshops); 2017 13-17-March; Kona, HI

107. Kubota N, Hiroyuki K, Taniguchi K, Sawayama T, editors. State estimation based on a spiking neural network using ultrasonic oscillosensors. 2008 World Automation Congress; 2008 28-Sept.-2; Waikoloa, HI, USA

108. Morita T, Taki K, Fujimoto M, Suwa H, Arakawa Y, Yasumoto K, editors. BLE Beacon-based Activity Monitoring System toward Automatic Generation of Daily Report. SmarterAALSmarterAAL'18: Workshop on Advanced Technologies for Smarter Assisted Living solutions: Towards an open Smart Home infrastructure; 2018 19-23-March; Athens, Greece.

109. Muckell J, Young Y, Leventhal M. A Wearable Motion Tracking System to Reduce Direct Care Worker Injuries. Proceedings of the 2017 International Conference on Digital Health; 2017; London, United Kingdom: Association for Computing Machinery; 2017. p. 202-6.

110. Swangnetr M, Kaber DB. Emotional State Classification in Patient–Robot Interaction Using Wavelet Analysis and Statistics-Based Feature Selection. IEEE Transactions on Human-Machine Systems. 2013 2013;43(1):63-75. doi: 10.1109/tsmca.2012.2210408.

111. Toda K, Shinomiya N, editors. Machine learning-based fall detection system for the elderly using passive RFID sensor tags. 2019 13th International Conference on Sensing Technology (ICST); 2019 2-4-Dec.; Sydney, NSW, Australia

112. Volrathongchai K. Predicting falls among the elderly residing in long-term care facilities using knowledge discovery in databases: University of Wisconsin - Madison; 2005.

113. Wu Q, Zhao W, editors. Machine Learning Based Human Activity Detection in a Privacy-Aware Compliance Tracking System. 2018 IEEE International Conference on Electro/Information Technology (EIT); 2018 3-5-May; Rochester, MI, USA.

114. Yamazaki K, Yamazaki A, Ikeda K, Liu C, Fukushima M, Kobayashi Y, et al. “I’ll Be There Next”. ACM Transactions on Interactive Intelligent Systems. 2016 2016;5(4):1-20. doi: 10.1145/2844542.

115. Yu S, Yang Y, Hauptmann A, editors. Harry Potter's Marauder's Map: Localizing and Tracking Multiple Persons-of-Interest by Nonnegative Discretization. 2013 IEEE Conference on Computer Vision and Pattern Recognition; 2013 23-28-June; Portland, OR, USA

116. Alam MAU, Roy N, Holmes S, Gangopadhyay A, Galik E, editors. Automated Functional and Behavioral Health Assessment of Older Adults with Dementia. 2015 IEEE First Conference on Connected Health: Applications, Systems and Engineering Technologies; 2016 27-29-June; Washington, DC, USA

117. Albina EM, Hernandez AA, editors. Designing an Intelligent Elderly Behavior Detection System. 2018 4th International Conference on Biosignals, Images and Instrumentation (ICBSII); 2018 22-24-March; Chennai.

118. Easton K, Potter S, Bec R, Bennion M, Christensen H, Grindell C, et al. A Virtual Agent to Support Individuals Living With Physical and Mental Comorbidities: Co-Design and Acceptability Testing. Journal of medical Internet research. 2019 May 30;21(5):e12996. PMID: 31148545. doi: 10.2196/12996.

119. Kuspinar A, Hirdes JP, Berg K, McArthur C, Morris JN. Development and validation of an algorithm to assess risk of first-time falling among home care clients. BMC geriatrics. 2019 Oct 14;19(1):264. PMID: 31610776. doi: 10.1186/s12877-019-1300-2.

120. Mano LY, editor. Emotional condition in the Health Smart Homes environment: emotion recognition using ensemble of classifiers. 2018 Innovations in Intelligent Systems and Applications (INISTA); 2018 3-5-July; Thessaloniki, Greece

121. Shen VRL, Horng-Yih L, Ah-Fur L, editors. Application of High-Level Fuzzy Petri Nets to fall detection system using smartphone. Proceedings of the 2013 International Conference on Machine Learning and Cybernetics; 2013 14-17-July; Tianjin, China.

122. Nguyen-Truong CKY, Fritz RL. Health-Assistive Smart Homes for Aging in Place: Leading the Way for Integration of the Asian Immigrant Minority Voice. Asian Pac Isl Nurs J. 2018 2018;3(4):154-9. PMID: 31037263. doi: 10.31372/20180304.1087.

123. Ghose S, Mitra J, Karunanithi M, Dowling J. Human Activity Recognition from Smart-Phone Sensor Data using a Multi-Class Ensemble Learning in Home Monitoring...Australian National Health Informatics Conference 2015. Studies in Health Technology & Informatics. 2015 2015;214:62-7. doi: 10.3233/978-1-61499-558-6-62.

124. Su C-J, Chiang C-Y. Pervasive community care platform: Ambient Intelligence leveraging sensor networks and mobile agents. International Journal of Systems Science. 2013 2014;45(4):778-97. doi: 10.1080/00207721.2013.807384.

125. Yuan W, Cao N, Wang Y, Li C, Wang X, Zhou L, editors. The Research of Elderly Health-Care in Wireless Sensor Networks. 2017 IEEE International Conference on Computational Science and Engineering (CSE) and IEEE International Conference on Embedded and Ubiquitous Computing (EUC); 2017 21-24-July; Guangzhou, China.

126. Uronen L, Moen H, Teperi S, Martimo KP, Hartiala J, Salantera S. Towards automated detection of psychosocial risk factors with text mining. Occupational medicine (Oxford, England). 2020 May 27;70(3):203-6. PMID: 32086511. doi: 10.1093/occmed/kqaa022.

127. Vuono A, Luperto M, Banfi J, Basilico N, Borghese NA, Sioutis M, et al. Seeking Prevention of Cognitive Decline in Elders via Activity Suggestion by A Virtual Caregiver (Demonstration). 17th International Conference on Autonomous Agents and Multiagent Systems (AAMAS 2018); Stockholm, Sweden2018. p. 1835-7.

128. Babič F, Jančuš A, Melišová K, editors. Customized Web-based System for Elderly People Using Elements of Artificial Intelligence. 2016 Federated Conference on Computer Science and Information Systems (FedCSIS); 2016 2016; Gdansk, Poland IEEE.

129. Bakola I, Bellos C, Bibas A, Koutsouris DD, Fotiadis DI, editors. An Ambient Intelligent monitoring system to improve the independency of the elderly with balance disorders indoors. IEEE-EMBS International Conference on Biomedical and Health Informatics (BHI); 2014 2014: IEEE.

130. Becker M, Werkman E, Anastasopoulos M, Kleinberger T, Chachques JC, Varshney U, editors. Approaching Ambient Intelligent Home Care Systems. Pervasive Health Conference and Workshops; 2006 2006; Innsbruck, Austria: IEEE.

131. Bizjak J, Gradisek A, Gams M, editors. Intelligent Assistant for Elderly. Twenty-Seventh International Joint Conference on Artificial Intelligence (IJCAI-18); 2018 2018.

132. Fan X, Zhao Y, Wang H, Tsui KL. Forecasting one-day-forward wellness conditions for community-dwelling elderly with single lead short electrocardiogram signals. BMC medical informatics and decision making. 2019 Dec-30;19(1):285. PMID: 31888608. doi: 10.1186/s12911-019-1012-8.

133. Feng P, Yu M, Naqvi SM, Chambers JA, editors. Deep learning for posture analysis in fall detection. 19th International Conference on Digital Signal Processing; 2014 20-23-Aug.; Hong Kong, China.

134. Fleury A, Vacher M, Noury N. SVM-Based Multimodal Classification of Activities of Daily Living in Health Smart Homes: Sensors, Algorithms, and First Experimental Results. IEEE Transactions on Information Technology in Biomedicine. 2010 2010;14(2):274-83. doi: 10.1109/TITB.2009.2037317.

135. Ganesh D, Seshadri G, Sokkanarayanan S, Rajan S, Sathiyanarayanan M, editors. IoT-based Google Duplex Artificial Intelligence Solution for Elderly Care. 2019 International Conference on contemporary Computing and Informatics (IC3I); 2019 12-14-Dec.; Singapore.

136. Gochoo M, Tan T, Velusamy V, Liu S, Bay, uuren D, et al. Device-Free Non-Privacy Invasive Classification of Elderly Travel Patterns in a Smart House Using PIR Sensors and DCNN. IEEE Sensors Journal. 2018 2018;18(1):390-400. doi: 10.1109/JSEN.2017.2771287.

137. Gómez-Sebastià I, Moreno J, Álvarez-Napagao S, Garcia-Gasulla D, Barrué C, Cortés U. Situated Agents and Humans in Social Interaction for Elderly Healthcare: From Coaalas to AVICENA. Journal of medical systems. 2016 Feb;40(2):38. PMID: 26590976. doi: 10.1007/s10916-015-0371-7.

138. Grgurić A, Mošmondor M, Huljenić D. The SmartHabits: An Intelligent Privacy-Aware Home Care Assistance System. Sensors (Basel, Switzerland). 2019 Feb-21;19(4). PMID: 30795587. doi: 10.3390/s19040907.

139. Haigh KZ, Kiff LM, Ho G. The independent lifestyle assistant: lessons learned. Assistive Technology. 2006 Spring2006;18(1):87-106. doi: 10.1080/10400435.2006.10131909.

140. Howcroft J, Kofman J, Lemaire ED. Feature selection for elderly faller classification based on wearable sensors. Journal of NeuroEngineering & Rehabilitation (JNER). 2017 2017;14:1-11. doi: 10.1186/s12984-017-0255-9.

141. Hsu H, Cheng Z, Shih TK, Chen C, editors. RFID-Based Personalized Behavior Modeling. 2009 Symposia and Workshops on Ubiquitous, Autonomic and Trusted Computing; 2009 7-9-July; Brisbane, QLD, Australia

142. Hu X, Li Z, Dai R, Cui Y, Zhou Z, An B, et al. Coarse-to-Fine Activity Annotation and Recognition Algorithm for Solitary Older Adults. IEEE Access. 2020 2020;8:4051-64. doi: 10.1109/ACCESS.2019.2962843.

143. Juang L, Wu M, editors. Abnormal action tracking using robot vision system. 2013 International Joint Conference on Awareness Science and Technology & Ubi-Media Computing (iCAST 2013 & UMEDIA 2013); 2013 2-4-Nov.

144. Juang L-H, Wu M-N. Fall Down Detection Under Smart Home System. Journal of medical systems. 2015 2015;39(10):1-12. doi: 10.1007/s10916-015-0286-3.

145. Kang K-J, Ka B, Kim SJ. A service scenario generation scheme based on association rule mining for elderly surveillance system in a smart home environment. Eng Appl Artif Intell. 2012 2012;25(7):1355--64. doi: 10.1016/j.engappai.2012.02.003.

146. Kasteren TL, Englebienne G, Kröse BJ. An activity monitoring system for elderly care using generative and discriminative models. Personal Ubiquitous Comput. 2010 2010;14(6):489–98. doi: 10.1007/s00779-009-0277-9.

147. Lei X, Chen J, Li G, Chen F, Longshen W, editors. A Design and Implementation of an Intelligent Care System for the Elderly Based on Internet of Things. 2019 Eleventh International Conference on Advanced Computational Intelligence (ICACI); 2019 2019; Guilin, China: IEEE.

148. Niiuchi R, Kang H, Iwamura K, editors. Detection of Human Motion Gestures Using Machine Learning for Actual Emergency Situations. 2018 IEEE 7th Global Conference on Consumer Electronics (GCCE); 2018 9-12-Oct.; Nara, Japan.

149. Nuutinen M, Leskelä R-L, Suojalehto E, Tirronen A, Komssi V. Development and validation of classifiers and variable subsets for predicting nursing home admission. BMC Medical Informatics & Decision Making. 2017 2017;17:1-12. doi: 10.1186/s12911-017-0442-4.

150. Okano T, Kitakoshi D, Suzuki M, editors. A Preliminary Study on Preventive Care System Based on Game Playing with Communication Robots. 2013 Conference on Technologies and Applications of Artificial Intelligence; 2013 6-8-Dec.; Taipei, Taiwan.

151. Pahl C, Varadarajan KM, editors. Emotional feedback for service robots using hapto-acoustic interface. 2015 Pattern Recognition Association of South Africa and Robotics and Mechatronics International Conference (PRASA-RobMech); 2015 26-27-Nov.; Port Elizabeth, South Africa.

152. Richter J, Hirtz G, editors. Novel methods for feature extraction based on motion history images and evaluation with regard to altering viewing angles. 2013 IEEE Third International Conference on Consumer Electronics, Berlin (ICCE-Berlin); 2013 9-11-Sept.; Berlin, Germany.

153. Sadreazami H, Bolic M, Rajan S, editors. On the Use of Ultra Wideband Radar and Stacked LSTM-RNN for at Home Fall Detection. 2018 IEEE Life Sciences Conference (LSC); 2018 28-30-Oct.; Montreal, QC, Canada.

154. Sadreazami H, Bolic M, Rajan S, editors. Residual Network-Based Supervised Learning of Remotely Sensed Fall Incidents using Ultra-Wideband Radar. 2019 IEEE International Symposium on Circuits and Systems (ISCAS); 2019 26-29-May; Sapporo, Japan.

155. Sadreazami H, Bolic M, Rajan S. CapsFall: Fall Detection Using Ultra-Wideband Radar and Capsule Network. IEEE Access. 2019 2019;7:55336-43. doi: 10.1109/ACCESS.2019.2907925.

156. Sakakibara S, Saiki S, Nakamura M, Yasuda K, Duffy VG, editors. Generating Personalized Dialogue Towards Daily Counseling System for Home Dementia Care. International Conference on Digital Human Modeling and Applications in Health, Safety, Ergonomics and Risk Management; 2017 2017; Vancouver, BC, Canada: Springer.

157. Soni PK, Choudhary A, editors. Automated Fall Detection From a Camera Using Support Vector Machine. 2019 Second International Conference on Advanced Computational and Communication Paradigms (ICACCP); 2019 25-28-Feb.: IEEE.

158. Suryadevara NK, Mukhopadhyay SC, editors. An intelligent system for continuous monitoring of wellness of an inhabitant for sustainable future. 2014 IEEE Region 10 Humanitarian Technology Conference (R10 HTC); 2014 6-9-Aug.; Chennai, India.

159. Suryadevara NK, Quazi T, Mukhopadhyay SC, editors. Intelligent Sensing Systems for Measuring Wellness Indices of the Daily Activities for the Elderly. 2012 Eighth International Conference on Intelligent Environments; 2012 2012; Guanajuato, Mexico: IEEE.

160. Suzuki T, Jose Y, Nakauchi Y, editors. A medication support system for an elderly person based on intelligent environment technologies. 2011 IEEE International Conference on Systems, Man, and Cybernetics; 2011 2011; Anchorage, AK, USA: IEEE.

161. Tokunaga S, Horiuchi H, Tamamizu K, Saiki S, Nakamura M, Yasuda K, editors. Deploying service integration agent for personalized smart elderly care. 2016 IEEE/ACIS 15th International Conference on Computer and Information Science (ICIS); 2016 26-29-June; Okayama, Japan.

162. Weimin H, Tuan Kiang C, Haizhou L, Tian Shiang K, Jit B, editors. Scream detection for home applications. 2010 5th IEEE Conference on Industrial Electronics and Applications; 2010 15-17-June; Taichung, Taiwan.

163. Wong B, Ho GTS, Tsui E. Development of an intelligent e-healthcare system for the domestic care industry. Ind Manag Data Syst. 2017 2017;117(7):1426--45. doi: 10.1108/IMDS-08-2016-0342.

164. Wu L, Lu J, Zhang T, Gong J, editors. Robot-assisted intelligent emergency system for individual elderly independent living. 2016 IEEE Global Humanitarian Technology Conference (GHTC); 2016 2016; Seattle, WA, USA IEEE.

165. Yu BXB, Chan KCC, editors. Discovering Knowledge by Behavioral Analytics for Elderly Care. 2017 IEEE International Conference on Big Knowledge (ICBK); 2017 9-10-Aug.; Hefei, China: IEEE.

166. Yu M, Rhuma A, Naqvi SM, Wang L, Chambers J. A Posture Recognition-Based Fall Detection System for Monitoring an Elderly Person in a Smart Home Environment. IEEE Transactions on Information Technology in Biomedicine. 2012 2012;16(6):1274-86. doi: 10.1109/TITB.2012.2214786.

167. Yu M, Yu Y, Rhuma A, Naqvi SMR, Wang L, Chambers JA. An Online One Class Support Vector Machine-Based Person-Specific Fall Detection System for Monitoring an Elderly Individual in a Room Environment. IEEE journal of biomedical and health informatics. 2013 2013;17(6):1002-14. doi: 10.1109/JBHI.2013.2274479.

168. Zeng Y, Chang W, editors. Estimation of sleep status based on wearable free device for elderly care. 2016 IEEE 5th Global Conference on Consumer Electronics; 2016 11-14-Oct.; Kyoto, Japan IEEE.

169. Zeng Y, Liao KH, Wang C, Lin Y, Chang W, editors. Implementation of post-operative wound analytics. 2017 IEEE International Conference on Consumer Electronics - Taiwan (ICCE-TW); 2017 12-14-June; Taipei: IEEE.

170. Paul AS, Wan EA, Adenwala F, Schafermeyer E, Preiser N, Kaye J, et al. MobileRF: A Robust Device-Free Tracking System Based On a Hybrid Neural Network HMM Classifier. Proceedings of the ACM International Conference on Ubiquitous Computing UbiComp (Conference). 2014 2014;2014:159-70. doi: 10.1145/2632048.2632097.

171. Veyron JH, Friocourt P, Jeanjean O, Luquel L, Bonifas N, Denis F, et al. Home care aides' observations and machine learning algorithms for the prediction of visits to emergency departments by older community-dwelling individuals receiving home care assistance: A proof of concept study. PloS one. 2019 2019;14(8):e0220002. PMID: 31408458. doi: 10.1371/journal.pone.0220002.

172. An Y, Khare R, Song I-Y, Hu X. Data exploration and knowledge discovery in a patient wellness tracking (PWT) system at a nurse-managed health services center. IHI '12: Proceedings of the 2nd ACM SIGHIT International Health Informatics Symposium; 2012; Miami, Florida, USA: Association for Computing Machinery; 2012.

173. Chen Y, Hsu J, Hung C, Wu J, Lai F, Kuo S. Surgical Wounds Assessment System for Self-Care. IEEE Transactions on Systems, Man, and Cybernetics: Systems. 2018 2018;50(12):1-16. doi: 10.1109/TSMC.2018.2856405.

174. Cheng L, Zhu M, Poss JW, Hirdes JP, Glenny C, Stolee P. Opinion versus practice regarding the use of rehabilitation services in home care: an investigation using machine learning algorithms. BMC medical informatics and decision making. 2015 Oct-9;15:80. PMID: 26453354. doi: 10.1186/s12911-015-0203-1.

175. Clark DG, Kapur P, Geldmacher DS, Brockington JC, Harrell L, DeRamus TP, et al. Latent information in fluency lists predicts functional decline in persons at risk for Alzheimer disease. Cortex; a journal devoted to the study of the nervous system and behavior. 2014 Jun;55:202-18. PMID: 24556551. doi: 10.1016/j.cortex.2013.12.013.

176. Freed M, Burns B, Heller A, Sanchez D, Beaumont-Bowman S, editors. A Virtual Assistant to Help Dysphagia Patients Eat Safely at Home. IJCAI'16: Proceedings of the Twenty-Fifth International Joint Conference on Artificial Intelligence; 2016 2016.

177. Jung K, Covington S, Sen CK, Januszyk M, Kirsner RS, Gurtner GC, et al. Rapid identification of slow healing wounds. Wound repair and regeneration : official publication of the Wound Healing Society [and] the European Tissue Repair Society. 2016 Jan-Feb;24(1):181-8. PMID: 26606167. doi: 10.1111/wrr.12384.

178. Ohura N, Mitsuno R, Sakisaka M, Terabe Y, Morishige Y, Uchiyama A, et al. Convolutional neural networks for wound detection: the role of artificial intelligence in wound care. Journal of wound care. 2019 Oct-1;28:S13-s24. PMID: 31600101. doi: 10.12968/jowc.2019.28.Sup10.S13.

179. Stiglic G, Kokol P, editors. Intelligent Patient and Nurse Scheduling in Ambulatory Health Care Centers. 2005 IEEE Engineering in Medicine and Biology 27th Annual Conference; 2005 17-18-Jan.; Shanghai, China: IEEE.

180. Li M, Xu G, He B, Ma X, Xie J. Pre-Impact Fall Detection Based on a Modified Zero Moment Point Criterion Using Data From Kinect Sensors. IEEE Sensors Journal. 2018 2018;18(13):5522-31. doi: 10.1109/JSEN.2018.2833451.

181. Yang J, Li J, Bai D, Sun B, Wang S, editors. Assistive standing of omni-direetional mobile rehabilitation training robot based on support vector regression algorithm. 2016 IEEE International Conference on Information and Automation (ICIA); 2016 1-3-Aug.; Ningbo, China: IEEE.

182. Ghosh N, Maity S, Maity K, Saha S, editors. Non-Parametric Learning Technique for Activity Recognition in Elderly Patients. TENCON 2019 - 2019 IEEE Region 10 Conference (TENCON); 2019 17-20-Oct.; Kochi, India: IEEE.

183. Inoue S, Mairittha T, Mairittha N, Hossain T, editors. Integrating Activity Recognition and Nursing Care Records: the System, Experiment, and the Dataset. 2019 Joint 8th International Conference on Informatics, Electronics & Vision (ICIEV) and 2019 3rd International Conference on Imaging, Vision & Pattern Recognition (icIVPR); 2019 30-May-2; Spokane, WA, USA IEEE.

184. Jalal A, Kamal S, Kim D. A Depth Video Sensor-Based Life-Logging Human Activity Recognition System for Elderly Care in Smart Indoor Environments. Sensors. 2014 2014;14(7):11735-59. doi: 10.3390/s140711735.

185. Jin S-Y, Jeong Y-S, Park C, Oh K, Choi H-J. An Intelligent Multi-Sensor Surveillance System for Elderly Care. Smart CR. 2012 2012;2(4):296-307. doi: 10.6029/smartcr.2012.04.006.

186. Khan SS, Zhu T, Ye B, Mihailidis A, Iaboni A, Newman K, et al., editors. DAAD: A Framework for Detecting Agitation and Aggression in People Living with Dementia Using a Novel Multi-modal Sensor Network. 2017 IEEE International Conference on Data Mining Workshops (ICDMW); 2017 18-21-Nov.; New Orleans, LA, USA: IEEE.

187. Kumar M, Teso S, Causmaecker PD, Raedt LD, editors. Automating Personnel Rostering by Learning Constraints Using Tensors. 2019 IEEE 31st International Conference on Tools with Artificial Intelligence (ICTAI); 2019 4-6-Nov.; Portland, OR, USA: IEEE.

188. Liu W, Chuang K, Chen K, editors. The Design and Implementation of a Chatbot's Character for Elderly Care. 2018 International Conference on System Science and Engineering (ICSSE); 2018 28-30-June; New Taipei, Taiwan: IEEE.

189. Machanje DI, Orero JO, Marsala C, editors. Distress Recognition from Speech Analysis: A Pairwise Association Rules-Based Approach. 2019 IEEE Symposium Series on Computational Intelligence (SSCI); 2019 6-9-Dec.; Xiamen, China: IEEE.

190. Mori Y, Ikeura R, Ding M, editors. Using tactile sensors to estimate care receiver position on dual arms of robot. SENSORS, 2013 IEEE; 2013 3-6-Nov.; Baltimore, MD, USA IEEE.

191. Nii M, Okajima S, Sakashita R, Hamada M, Kobashi S, editors. Tongue movement classification in chewing and swallowing using electromyography. 2017 6th International Conference on Informatics, Electronics and Vision & 2017 7th International Symposium in Computational Medical and Health Technology (ICIEV-ISCMHT); 2017 1-3-Sept.; Himeji: IEEE.

192. Nii M, Takahama K, Uchinuno A, Sakashita R, editors. Soft class decision for nursing-care text classification using a k-nearest neighbor based system. 2014 IEEE International Conference on Fuzzy Systems (FUZZ-IEEE); 2014 6-11-July; Beijing, China IEEE.

193. Nii M, Takahama K, Uchinuno A, Sakashita R, editors. A directed graph based feature definition for classifying nursing-care texts. 2014 IEEE International Conference on Systems, Man, and Cybernetics (SMC); 2014 5-8-Oct.; San Diego, CA, USA IEEE.

194. Nii M, Takahama K, Uchinuno A, Sakashita R, editors. A matrix-based feature vector definition and a SVM-BDT-based classification system for classifying nursing-care texts. 2015 IEEE International Conference on Fuzzy Systems (FUZZ-IEEE); 2015 2-5-Aug.; Istanbul, Turkey IEEE.

195. Nii M, Tuchida Y, Iwamoto T, Uchinuno A, Sakashita R, editors. Nursing-care text evaluation using word vector representations realized by word2vec. 2016 IEEE International Conference on Fuzzy Systems (FUZZ-IEEE); 2016 24-29-July; Vancouver, BC, Canada: IEEE.

196. Ongenae F, Duysburgh P, Verstraete M, Sulmon N, Bleumers L, Jacobs A, et al., editors. User-driven design of a context-aware application: An ambient-intelligent nurse call system. 2012 6th International Conference on Pervasive Computing Technologies for Healthcare (PervasiveHealth) and Workshops; 2012 2012; San Diego, CA, USA IEEE.

197. Pessemier TD, Dooms S, Martens L. A food recommender for patients in a care facility. RecSys '13: Proceedings of the 7th ACM conference on Recommender systems; 2013; Hong Kong, China: Association for Computing Machinery; 2013.

198. Phan R, Luu T, Davey R, Chetty G, editors. Enhancing Clinical Name Entity Recognition Based on Hybrid Deep Learning Scheme. 2019 International Conference on Data Mining Workshops (ICDMW); 2019 8-11-Nov.; Beijing, China IEEE.

199. Ramanujam E, Padmavathi S. A Vision-Based Posture Monitoring System for the Elderly Using Intelligent Fall Detection Technique. In: Mahmood Z, editor. Guide to Ambient Intelligence in the IoT Environment. Cham: Springer; 2019. p. 249-69.

200. Tian X. Research on the realization path of smart old-age care in Suzhou based on intelligent recommendation system. J Intell Fuzzy Syst. 2018 2018;35(3):3051-63. doi: 10.3233/JIFS-169660.

201. Di Pietro TL, Doran DM, McArthur G. Supportive decision making at the point of care: refinement of a case-based reasoning application for use in nursing practice. Computers, informatics, nursing : CIN. 2010 Jul-Aug;28(4):235-40. PMID: 20571376. doi: 10.1097/NCN.0b013e3181e1e77a.

202. Wai AAP, Yuan-Wei K, Fook FS, Jayach, ran M, Biswas J, et al., editors. Sleeping patterns observation for bedsores and bed-side falls prevention. 2009 Annual International Conference of the IEEE Engineering in Medicine and Biology Society; 2009 3-6-Sept.; Minneapolis, MN, USA: IEEE.

203. Wickramasinghe A, Ranasinghe DC, Fumeaux C, Hill KD, Visvanathan R. Sequence Learning with Passive RFID Sensors for Real-Time Bed-Egress Recognition in Older People. IEEE journal of biomedical and health informatics. 2017 2017;21(4):917-29. doi: 10.1109/JBHI.2016.2576285.

204. Sinn CJ, Jones A, McMullan JL, Ackerman N, Curtin-Telegdi N, Eckel L, et al. Derivation and validation of the Personal Support Algorithm: an evidence-based framework to inform allocation of personal support services in home and community care. BMC health services research. 2017 Nov-25;17(1):775. PMID: 29178868. doi: 10.1186/s12913-017-2737-7.

205. Batata O, Augusto V, Xie X. Mixed machine learning and agent-based simulation for respite care evaluation. 2018 Winter Simulation Conference (WSC); 2018; Gothenburg, Sweden: IEEE Press; 2018. p. 2668–79.

206. Gomes CD, Silva E. Oliveira LE, Cubas MR, Cabral Moro Barra CM. Use of computational tools as support to the cross-mapping nethod between clinical terminologies. Texto & Contexto Enfermagem. 2019 2019;28:1-12. doi: 10.1590/1980-265x-tce-2017-0187.

207. Demmer J, Kitzig A, Naroska E, editors. Improvements of a retrospective analysis method for a HMM based posture recognition system in a functionalized nursing bed. 2017 IEEE/SICE International Symposium on System Integration (SII); 2017 2017; Taipei, Taiwan: IEEE.

208. Esfahani SN, Muthukumar V, Regentova EE, Taghva K, Trabia M, editors. Complex Food Recognition using Hyper-Spectral Imagery. 2020 10th Annual Computing and Communication Workshop and Conference (CCWC); 2020 6-8-Jan.; Las Vegas, NV, USA: IEEE.

209. Fodeh SJ, Finch D, Bouayad L, Luther SL, Ling H, Kerns RD, et al. Classifying clinical notes with pain assessment using machine learning. Medical & biological engineering & computing. 2018 Jul;56(7):1285-92. PMID: 29280092. doi: 10.1007/s11517-017-1772-1.

210. Hsu CC, Wang MY, Shen HCH, Chiang RH, Wen CHP, editors. FallCare+: An IoT surveillance system for fall detection. 2017 International Conference on Applied System Innovation (ICASI); 2017 13-17-May; Sapporo, Japan IEEE.

211. Huimin Q, Yaobin M, Wenbo X, Zhiquan W, editors. Home environment fall detection system based on a cascaded multi-SVM classifier. 2008 10th International Conference on Control, Automation, Robotics and Vision; 2008 17-20-Dec.; Hanoi, Vietnam IEEE.

212. Johnson WG. CI for a DSS to predict nursing coverage in a geriatric care facility. Studies in health technology and informatics. 2006 2006;122:86-90. PMID: 17102223.

213. Lee H, Kim J, Yang D, Kim J-H. Embedded Real-Time Fall Detection Using Deep Learning For Elderly Care. 31st Conference on Neural Information Processing Systems (NIPS 2017); 2017; Long Beach, CA, USA2017.

214. Luo S, Zhao C, Fu Y, editors. An Intelligent Human Activity Recognition Method with Incremental Learning Capability for Bedridden Patients. 2018 15th International Conference on Control, Automation, Robotics and Vision (ICARCV); 2018 18-21-Nov.; Singapore IEEE.

215. Luu TM, Phan R, Davey R, Chetty G, editors. A Multilevel NER Framework for Automatic Clinical Name Entity Recognition. 2017 IEEE International Conference on Data Mining Workshops (ICDMW); 2017 18-21-Nov.; New Orleans, LA, USA IEEE.

216. Magyar J, Kobayashi M, Nishio S, Sinčák P, Ishiguro H, editors. Autonomous Robotic Dialogue System with Reinforcement Learning for Elderlies with Dementia. 2019 IEEE International Conference on Systems, Man and Cybernetics (SMC); 2019 6-9-Oct.; Bari, Italy IEEE.

217. Masafumi Y, Tomohiro Y, Takeshi F, editors. Study on effect of MOGA with interactive island model using visualization. IEEE Congress on Evolutionary Computation; 2010 18-23-July; Barcelona, Spain IEEE.

218. Mihailidis A, Boger J, Canido M, Hoey J. The use of an intelligent prompting system for people with dementia. Interactions. 2007 2007;14(4):34--7. doi: 10.1145/1273961.1273982.

219. Mitabe N, Shinomiya N, editors. An intelligent care support system for the elderly with an autonomous robot in ambient sensors. 2017 IEEE 6th Global Conference on Consumer Electronics (GCCE); 2017 24-27-Oct; Nagoya, Japan IEEE.

220. Mitabe N, Shinomiya N, editors. An intelligent support system for elderly care with RFID tags and a cleaning robot. 2018 12th International Conference on Sensing Technology (ICST); 2018 4-6-Dec.; Limerick, Ireland IEEE.

221. Morita K, Fuketa M, Aoe J-i, Yasuda K. Improved dialogue communication systems for individuals with dementia. IJCAT. 2015 2015;52(2):127--34. doi: 10.1504/IJCAT.2015.071973.

222. Si H, Kim SJ, Kawanishi N, Morikawa H, editors. An Guidance System Based on Q-Learning for Supporting Dementia Patient's Activities of Daily Living. 2007 4th IEEE Consumer Communications and Networking Conference; 2007 2007; Las Vegas, NV, USA IEEE.

223. Si H, Kim SJ, Kawanishi N, Morikawa H, editors. A Context-aware Reminding System for Daily Activities of Dementia Patients. 27th International Conference on Distributed Computing Systems Workshops (ICDCSW'07); 2007 2007; Toronto, ON, Canada: IEEE Computer Society.

224. Takadama K, Hirose K, Matsushima H, Hattori K, Nakajima N. Learning Multiple Band-Pass Filters for Sleep Stage Estimation: Towards Care Support for Aged Persons. IEICE Trans Commun. 2010 2010;93(4):811-8. doi: 10.1587/transcom.E93.B.811.

225. Wei Z, Ju ZX, Chun X, Hua J, Jin P, editors. An Automatic Electronic Nursing Records Analysis System Based on the Text Classification and Machine Learning. 2013 5th International Conference on Intelligent Human-Machine Systems and Cybernetics; 2013 26-27-Aug.; Hangzhou, China IEEE.

226. Wiratanaya A, Lyons MJ, Butko NJ, Abe S. iMime: an interactive character animation system for use in dementia care. IUI '07: Proceedings of the 12th international conference on Intelligent user interfaces; 2007; Honolulu, Hawaii, USA: Association for Computing Machinery; 2007. p. 262–5.

227. Yamada Y, Saito T, Kawasaki S, Ikeda D, Katagiri M, Nishimura M, et al., editors. A Deep-Learning-Based Method of Estimating Water Intake. 2017 IEEE 41st Annual Computer Software and Applications Conference (COMPSAC); 2017 4-8-July; Turin, Italy: IEEE.

228. Asif U, Von Cavallar S, Tang J, Harrer S. SSHFD: Single Shot Human Fall Detection with Occluded Joints Resilience. ECAI 20082020.

229. Cyras K, Karamlou A, Lee M, Letsios D, Misener R, Toni F. AI-assisted Schedule Explainer for Nurse Rostering. AAMAS '20: Proceedings of the 19th International Conference on Autonomous Agents and MultiAgent Systems; 2020; Auckland, New Zealand: International Foundation for Autonomous Agents and Multiagent Systems; 2020.

230. Aziz O, Musngi M, Park EJ, Mori G, Robinovitch SN. A comparison of accuracy of fall detection algorithms (threshold-based vs. machine learning) using waist-mounted tri-axial accelerometer signals from a comprehensive set of falls and non-fall trials. Medical & biological engineering & computing. 2016 Jan;55(1):45-55. PMID: 27106749. doi: 10.1007/s11517-016-1504-y.

231. Belshaw M, Taati B, Snoek J, Mihailidis A. Towards a single sensor passive solution for automated fall detection. Conference proceedings : Annual International Conference of the IEEE Engineering in Medicine and Biology Society IEEE Engineering in Medicine and Biology Society Annual Conference. 2011 2011;2011:1773-6. PMID: 22254671. doi: 10.1109/IEMBS.2011.6090506.

232. Samra HE, Li AS, Soh B, Alzain MA, editors. A Conceptual Model for an Intelligent Simulation-Based Learning Management System Using a Data Mining Agent in Clinical Skills Education. 2016 4th International Conference on Enterprise Systems (ES); 2016 2-3-Nov.; Melbourne, VIC, Australia IEEE.

233. Adlassnig KP, Blacky A, Koller W. Artificial-intelligence-based hospital-acquired infection control. Studies in health technology and informatics. 2009 2009;149:103-10.

234. Arif D, Ahmad A, Bakar MA, Ihtisham MH, Winberg S. Cost Effective Solution for Minimization of Medical Errors and Acquisition of Vitals By Using Autonomous Nursing Robot. ICISDM '17: Proceedings of the 2017 International Conference on Information System and Data Mining; 2017; Charleston, SC, USA: Association for Computing Machinery; 2017.

235. Baxter GD, Monk AF, Tan K, Dear PR, Newell SJ. Using cognitive task analysis to facilitate the integration of decision support systems into the neonatal intensive care unit. Artificial intelligence in medicine. 2005 Nov;35(3):243-57. PMID: 15994070. doi: 10.1016/j.artmed.2005.01.004.

236. Ding B-F, Chang P, Wang P, Li H-T, Kuo M-C, Gundlapalli AV, et al. Design of an Intelligent Nursing Clinical Pathway and Nursing Order Support System for Traditional Chinese Medicine. Stud Health Technol Inform. 2017 2017;245:1014-8. doi: 10.3233/978-1-61499-830-3-1014.

237. Fernandes CO, Miles S, Lucena CJP, Cowan D. Artificial Intelligence Technologies for Coping with Alarm Fatigue in Hospital Environments Because of Sensory Overload: Algorithm Development and Validation. Journal of medical Internet research. 2019 Nov-26;21(11):e15406. PMID: 31769762. doi: 10.2196/15406.

238. Jordan D, Rose SE. Multimedia abstract generation of intensive care data: the automation of clinical processes through AI methodologies. World journal of surgery. 2010 Apr;34(4):637-45. PMID: 20012610. doi: 10.1007/s00268-009-0319-5.

239. Dingli A, Abela C. A pervasive assistant for nursing and doctoral staff. ECAI 20082008.

240. Tapia DI, Corchado JM. An Ambient Intelligence Based Multi-Agent System for Alzheimer Health Care. IJACI. 2009 2009;1(1):15--26. doi: 10.4018/jaci.2009010102.

241. Tseng KC, Hsu CL, Chuang YH. Designing an intelligent health monitoring system and exploring user acceptance for the elderly. Journal of medical systems. 2013 Dec;37(6):9967. PMID: 24037138. doi: 10.1007/s10916-013-9967-y.

242. Alnosayan N, Lee E, Alluhaidan A, Chatterjee S, Houston-Feenstra L, Kagoda M, et al., editors. MyHeart: An intelligent mHealth home monitoring system supporting heart failure self-care. 2014 IEEE 16th International Conference on e-Health Networking, Applications and Services (Healthcom); 2014 2014; Natal, Brazil: IEEE.

243. Chen Y-L, Chiang H-H, Yu C-W, Chiang C-Y, Liu C-M, Wang J-H. An Intelligent Knowledge-Based and Customizable Home Care System Framework with Ubiquitous Patient Monitoring and Alerting Techniques. Sensors. 2012 2012;12(8):11154-86. doi: 10.3390/s120811154.

244. Park C, Kim J, Chen J, Ma H, Stojmenovic I, editors. A Location and Emergency Monitoring System for Elder Care Using ZigBee. 2011 Seventh International Conference on Mobile Ad-hoc and Sensor Networks; 2011 2011; Beijing, China: IEEE Computer Society.

245. Augustyniak P, editor. Adaptive architecture for assisted living systems. 2013 6th International Conference on Human System Interactions (HSI); 2013 6-8-June; Sopot, Poland.

246. Hsu C-C, Chien YY, Yu G, Koppen M, Chen S-M, Niu X, editors. An Intelligent Fuzzy Affective Computing System for Elderly Living Alone. 2009 Ninth International Conference on Hybrid Intelligent Systems; 2009 2009; Shenyang, China: IEEE Computer Society.

247. Lim S, Chung L, Han O, Kim J-H, Lee S-H, Hanzo L, et al., editors. An interactive cyber-physical system (CPS) for people with disability and frail elderly people. ICUIMC '11: Proceedings of the 5th International Conference on Ubiquitous Information Management and Communication; 2011 2011: ACM.

248. Merico D, Bisiani R, Malizia F, Rizzi G, Ali H. Demonstrating Contexta-CARE: a situation-aware system for supporting independent living. 7th International Conference on Pervasive Computing Technologies for Healthcare; 2013; Venice, Italy: ICST (Institute for Computer Sciences, Social-Informatics and Telecommunications Engineering); 2013.

249. Shieh YY, Shieh M, editors. Consumer electronics-based intelligent alert system for unattended elderly residents. 2013 IEEE International Symposium on Consumer Electronics (ISCE); 2013 2013; Hsinchu, Taiwan: IEEE.

250. Tamamizu K, Tokunaga S, Saiki S, Matsumoto S, Nakamura M, Yasuda K, et al., editors. Towards Person-Centered Anomaly Detection and Support System for Home Dementia Care. International Conference on Digital Human Modeling and Applications in Health, Safety, Ergonomics and Risk Management; 2016 2016: Springer.

251. Thomas AM, Moore P, Evans C, Sharma M, Chima P, Vijay VC, et al., editors. Emotive Sensors for Intelligent Care Systems: A Heuristic Discussion of Autonomic Wireless Sensing Systems. 2013 Seventh International Conference on Complex, Intelligent, and Software Intensive Systems; 2013 2013; Taichung, Taiwan: IEEE Computer Society.

252. Wai AAP, Devi SS, Biswas J, a SK, Abdulrazak B, Giroux S, et al., editors. Pervasive Intelligence System to Enable Safety and Assistance in Kitchen for Home-Alone Elderly. ICOST'11: Proceedings of the 9th international conference on Toward useful services for elderly and people with disabilities: smart homes and health telematics; 2011 2011: Springer.

253. Wingrave CA, Rowe M, Greenstein S, editors. WIP: Designing Smart Systems to Support @Work Caregiver Needs. 2012 AAAI Fall Symposium Series; 2012 2012: AAAI.

254. Luperto M, Romeo M, Lunardini F, Basilico N, Jones R, Cangelosi A, et al. Digitalized Cognitive Assessment Mediated by a Virtual Caregiver. Proceedings of the Twenty-Seventh International Joint Conference on Artificial Intelligence (IJCAI-18)2018. p. 5841-3.

255. Chouvarda I, Philip NY, Natsiavas P, Kilintzis V, Sobnath D, Kayyali R, et al. WELCOME – innovative integrated care platform using wearable sensing and smart cloud computing for COPD patients with comorbidities. Conference proceedings : Annual International Conference of the IEEE Engineering in Medicine and Biology Society IEEE Engineering in Medicine and Biology Society Annual Conference. 2014 2014;2014:3180-3. PMID: 25570666. doi: 10.1109/EMBC.2014.6944298

256. Nie G, Ullal A, Swanson AR, Weitlauf AS, Warren ZE, Sarkar N, et al., editors. Design of an Intelligent and Immersive System to Facilitate the Social Interaction Between Caregivers and Young Children with Autism. HCII 2019: International Conference on Human-Computer Interaction Universal Access in Human-Computer Interaction Multimodality and Assistive Environments 2019 2019: Springer.

257. Bleda AL, Maestre R, Beteta MA, Vidal JA, Dobre C, Melero FJ, et al., editors. AmICare: Ambient Intelligent and Assistive System for Caregivers Support. 2018 IEEE 16th International Conference on Embedded and Ubiquitous Computing (EUC); 2018 2018; Bucharest, Romania: IEEE Computer Society.

258. Nii M, Yamaguchi T, Takahashi Y, Uchinuno A, Sakashita R, editors. Fuzzy Rule Extraction from Nursing-Care Texts. 2009 39th International Symposium on Multiple-Valued Logic; 2009 21-23-May; Naha, Japan IEEE.

259. Vairaktarakis C, Tsiamis V, Soursou G, Lekkas F, Nikolopoulou M, Vasileiadou E, et al. A computer-aided diagnosis system for geriatrics assessment and frailty evaluation. In: P. V, A. A, editors. GeNeDis 2014 Advances in Experimental Medicine and Biology. Cham: Springer; 2015. p. 69-77.

260. Jung H, Park HA. Development and Evaluation of a Prototype CDSS for Fall Prevention. Studies in health technology and informatics. 2019 Aug-21;264:1700-1. PMID: 31438300. doi: 10.3233/SHTI190604

261. Yang J-Y, Kwon O-H, Lim C-S, Kwon D-S. Human-Robot Interaction-Based Intention Sharing of Assistant Robot for Elderly People. In: S L, H C, KJ Y, J L, editors. Intelligent Autonomous Systems 12 Advances in Intelligent Systems and Computing. Berllin, Heidelberg: Springer; 2013. p. 401-9.

262. Lu DF, Street WN, Delaney C. Knowledge discovery: Detecting elderly patients with impaired mobility. Studies in health technology and informatics. 2006 2006;122:121-3. PMID: 17102231.

263. Bouchard B, Roy P, Bouzouane A, Giroux S, Mihailidis A. An Activity Recognition Model for Alzheimer’s Patients: Extension of the COACH Task Guidance System. ECAI 20082008.

264. Zhou F, Jiao J, Chen S, Zhang D. A Case-Driven Ambient Intelligence System for Elderly in-Home Assistance Applications. IEEE Trans Syst Man Cybern Part C. 2011 2011;41(2):179--89. doi: 10.1109/TSMCC.2010.2052456.

265. Luštrek M, Kaluža B, Cvetković B, Dovgan E, Gjoreski H, Mirchevska V, et al. Confidence: Ubiquitous Care System to Support Independent Living. ECAI 20122012.

266. Yang C, Lu M, Tseng S, Fu L, editors. A companion robot for daily care of elders based on homeostasis. 2017 56th Annual Conference of the Society of Instrument and Control Engineers of Japan (SICE); 2017 19-22-Sept.; Kanazawa, Japan: IEEE.

267. Sadahiro T, Hamazaki M, Miyawaki F, Yoshimitsu K, Masamune K, editors. Laparoscopic skill measurement with COP to realize a HAM Scrub Nurse Robot system. 2007 IEEE International Conference on Systems, Man and Cybernetics; 2007 2007; Montreal, QC, Canada: IEEE.

268. Robben S, Bosch L, Wiggers P, Decancq J, Kanis M. Managing flexible care with a context aware system for ageing-in-place. 2015 9th International Conference on Pervasive Computing Technologies for Healthcare (PervasiveHealth); 2015; Istanbul, Turkey: ICST (Institute for Computer Sciences, Social-Informatics and Telecommunications Engineering); 2015.

269. Cesta A, Pecora F, Bickmore TW, editors. The ROBOCARE Project: Intelligent Systems for Elder Care. AAAI’s Fall Symposium; 2005 2005: AAAI Press.

270. Pascual J, Sanz-Bobi MA, Contreras D, Azevedo Li, Londral AR, editors. Intelligent System for Assisting Elderly People at Home. Proceedings of the First International Conference on Health Informatics; 2008 2008: INSTICC - Institute for Systems and Technologies of Information, Control and Communication.

271. Wang P, Low KH, Tani JW, Ch, ra T. Initial Study on a Home-Based Floor-MAT System for Fall Prevention of Elderly Based on Gait Analysis. I J Information Acquisition. 2010 2010;7(2):135-49. doi: 10.1142/S0219878910002129.

272. Weisenberg J, Cuddihy P, Rajiv V. Augmenting motion sensing to improve detection of periods of unusual inactivity. HealthNet '08: Proceedings of the 2nd International Workshop on Systems and Networking Support for Health Care and Assisted Living Environments; 2008; Breckenridge, Colorado: Association for Computing Machinery; 2008.

273. Xu T, Zhou Y, Ma Z, Duffy VG, editors. AtHoCare: An Intelligent Elder Care at Home System. International Conference on Digital Human Modeling and Applications in Health, Safety, Ergonomics and Risk Management DHM 2016; 2016 2016: Springer.

274. Zejda D, Callaghan V, Kameas A, Egerton S, Satoh I, Weber M, editors. Deep Design for Ambient Intelligence: Toward Acceptable Appliances for Higher Quality of Life of the Elderly. 2010 Sixth International Conference on Intelligent Environments; 2010 2010; Kuala Lumpur, Malaysia IEEE Computer Society.

275. Annicchiarico R, Barrué C, Benedico T, Campana F, Cortés U, Matínez-Velasco A. The i-Walker: an intelligent pedestrian mobility ai. In: Ghallab M, Spyropoulos CD, Fakotakis N, Avouris N, editors. ECAI 2008 - 18th European Conference on Artificial Intelligence; Patras, Greece2008.

276. Bono M, Sumi Y, Nishida T, editors. Towards Achieving Complex Medical Engineering to Understand Conversational Dynamics. 2007 IEEE/ICME International Conference on Complex Medical Engineering; 2007 23-27-May; Beijing, China: IEEE.

277. Westra BL, Peterson JJ. Big Data and Perioperative Nursing. AORN journal. 2016 Oct;104(4):286-92. PMID: 27692075. doi: 10.1016/j.aorn.2016.07.009.

278. O'Brien K, Liggett A, Ramirez‐Zohfeld V, Sunkara P, Lindquist LA. Voice‐Controlled Intelligent Personal Assistants to Support Aging in Place. Journal of the American Geriatrics Society. 2020 2020;68(1):176-9. doi: 10.1111/jgs.16217.

279. Shillan D, Sterne JAC, Champneys A, Gibbison B. Use of machine learning to analyse routinely collected intensive care unit data: a systematic review. Critical care (London, England). 2019 Aug-22;23(1):284. PMID: 31439010. doi: 10.1186/s13054-019-2564-9

280. Al-Shaqi R, Mourshed M, Rezgui Y. Progress in ambient assisted systems for independent living by the elderly. SpringerPlus. 2016 2016;5:624. PMID: 27330890. doi: 10.1186/s40064-016-2272-8.

281. Campos W, Rebollar AMi, Sanchez W, Estrada H, Castro-Sanchez NA, ro, et al. A Systematic Review of Proposals for the Social Integration of Elderly People Using Ambient Intelligence and Social Networking Sites. Cogn Comput. 2016 2016;8(3):529--42. doi: 10.1007/s12559-016-9382-z.

282. Krishnan RH, Pugazhenthi S. Mobility assistive devices and self-transfer robotic systems for elderly, a review. Intelligent Service Robotics. 2013 2014;7(1):37-49. doi: 10.1007/s11370-013-0142-6.

283. Rouleau G, Gagnon MP, Côté J, Payne-Gagnon J, Hudson E, Dubois CA. Impact of Information and Communication Technologies on Nursing Care: Results of an Overview of Systematic Reviews. Journal of medical Internet research. 2017 Apr-25;19(4):e122. PMID: 28442454. doi: 10.2196/jmir.6686

284. Cresswell K, Callaghan M, Khan S, Sheikh Z, Mozaffar H, Sheikh A. Investigating the use of data-driven artificial intelligence in computerised decision support systems for health and social care: A systematic review. Health informatics journal. 2020 Jan-22:1460458219900452. PMID: 31964204. doi: 10.1177/1460458219900452.

285. Khan SS, Ye B, Taati B, Mihailidis A. Detecting agitation and aggression in people with dementia using sensors-A systematic review. Alzheimer's & dementia : the journal of the Alzheimer's Association. 2018 Jun;14(6):824-32. PMID: 29571749. doi: 10.1016/j.jalz.2018.02.004.

286. Zahia S, Garcia Zapirain MB, Sevillano X, González A, ro, Kim PJ, et al. Pressure injury image analysis with machine learning techniques: A systematic review on previous and possible future methods. Artificial intelligence in medicine. 2020 2020;102:N.PAG-N.PAG. doi: 10.1016/j.artmed.2019.101742.

287. Gerke S, Yeung S, Cohen IG. Ethical and Legal Aspects of Ambient Intelligence in Hospitals. JAMA: Journal of the American Medical Association. 2020 2020;323(7):601-2. doi: 10.1001/jama.2019.21699.

288. Wangmo T, Lipps M, Kressig RW, Ienca M. Ethical concerns with the use of intelligent assistive technology: findings from a qualitative study with professional stakeholders. BMC medical ethics. 2019 Dec-19;20(1):98. PMID: 31856798. doi: 10.1186/s12910-019-0437-z.

289. Frize M, Yang L, Walker RC, O'Connor AM. Conceptual framework of knowledge management for ethical decision-making support in neonatal intensive care. IEEE transactions on information technology in biomedicine : a publication of the IEEE Engineering in Medicine and Biology Society. 2005 Jun;9(2):205-15. PMID: 16138537. doi: 10.1109/TITB.2005.847187.

290. Portacolone E, Halpern J, Luxenberg J, Harrison KL, Covinsky KE. Ethical Issues Raised by the Introduction of Artificial Companions to Older Adults with Cognitive Impairment: A Call for Interdisciplinary Collaborations. Journal of Alzheimer's disease : JAD. 2020 Mar-30. PMID: 32250295. doi: 10.3233/JAD-190952.

291. McCradden MD, Baba A, Saha A, Ahmad S, Boparai K, Fadaiefard P, et al. Ethical concerns around use of artificial intelligence in health care research from the perspective of patients with meningioma, caregivers and health care providers: a qualitative study. CMAJ open. 2020 Jan-Mar;8(1):E90-e5. PMID: 32071143. doi: 10.9778/cmajo.20190151.

292. Peirce AG, Elie S, George A, Gold M, O'Hara K, Rose-Facey W. Knowledge development, technology and questions of nursing ethics. Nursing ethics. 2020 Feb;27(1):77-87. PMID: 31032700. doi: 10.1177/0969733019840752.
