# Supplementary material for: Application Scenarios for Artificial Intelligence in Nursing Care: Rapid Review
Source: J Med Internet Res. 2021 Nov 29;23(11):e26522. doi: 10.2196/26522 (PMC8669587; doi:10.2196/26522)
Supplement: Multimedia Appendix 3 [file jmir_v23i11e26522_app3.docx]

| **Reference** | **Design** | **No. of participants and details** | **Setting** | **Type of data used  for AI approach** | **Research question/ primary objective** | **Outcome** | **Results** |
| --- | --- | --- | --- | --- | --- | --- | --- |
| Abdulla & Fakieh 2020  [1] | cross-sectional | 250; 121 nurses  (48.4 % of sample) | hospital | not specified | explore the level of employees’ knowledge about AI and perception of AI implementation differentiated by job type | perceptions and attitudes towards the implementation of AI technologies; questionnaire with items on perception, advantages and problems for AI application | 3.11 of 4 respondents feared AI would replace employees and had a general lack of knowledge regarding AI,  most respondents were unaware of the advantages and most common challenges to AI applications, indicating a need for training, technicians were most frequently impacted by AI applications |
| Ala-Kitula et al. 2017 [2] | mixed-methods, pilot testing of use-case | not stated | aLTC | sensor data | identification of use-cases; piloted use case: gather personal health data and to evaluate the home care need and its availability, in real time | daily activity before, during and after rehabilitation | AI technologies would not give any extra benefit for the use case than pre-existing solutions, there would be no need to use AI just for the sake of it. All of the 34 use cases identified used AI techniques, data analytics and NLP were the most frequently used techniques |
| Alderden et al. 2019 [3] | observational | 6376 | hospital | EHR data | develop a model for predicting pressure injuries among critical care patients | HAPIs classified as stage 1 to 4, deep-tissue injury, or unstageable and HAPIs classified as stage 2 to 4, deep-tissue injury, or unstageable | Random forest models were developed to predict stage 1 and greater and stage 2 and greater injuries by using the testing set to evaluate classifier performance. Area under the receiver operating characteristic curve for both models was 0.79. |
| Alwan 2009 [4] | mixed quantitative approaches | not stated; example 4: 22 subjects | aLTC | sensor data | develop a model for geriatric care enabled by in-home monitoring and ambient intelligence technologies | example 4: technological functionality | no detailed results reported |
| Amato et al. 2018 [5] | mixed methods, qualitative interviews | 5 patients, unclear number of doctors, caregivers | sLTC | sensor data | explore the application of remote monitoring technologies able to detect the onset of crises in people with AD, that may alleviate the psychological burden of caregivers | onset of crisis,  psychological burden | no detailed results from the evaluation of sensor data, multiple aspects regarding the interaction of both caregivers and patients emerged as critical, suggesting that the device was not appropriate for the context of use |
| Ambagtsheer et al. 2020 [6] | observational | 592; ≥75 years, Aged Care Funding Instrument (ACFI) assessment within the previous three year period | sLTC | routine data, residential aged care administrative data set | determine the effectiveness of AI algorithms in identifying frailty in comparison with a calculated electronic Frailty Index (eFI) | classification of frailty against a calculated e-Frailty Index, adaptation of Clegg’s 36-item eFI employs a binary checklist of 36 deficits | best prediction result was obtained using a SVM algorithm with 70 input variables (returning accuracy of 93.5%, Cohen’s Kappa and PABAK of 87%, sensitivity of 97.8% and specificity of 89.1%) |
| Antoniadi et al. 2020 [7] | observational | 90 ALS patients and their primary caregiver | hospital | interview data, register data | predict caregiver burden and identify related features using machine learning techniques | caregiver burden, Zarit Burden Interview score | caregiver’s quality of life and psychological  distress were the most predictive features of burden (0.92 sensitivity and 0.78 specificity)* |
| Bajo et al. 2008 [8]* | observational | 10 nurses | sLTC | EHR data, sensor data | develop a multi-agent architecture for facilitating health care | number of nurses needed per hour, time spent on supervision and control tasks | time spent on supervision and control tasks reduced substantially, as well as the time spent attending false alarms, while the time for direct patient care increased |
| Bickmore et al. 2009 [9] | observational | 19 | hospital | N/A | determine the usability and acceptance an animated virtual  nurse that teaches patients about their post-discharge self-care | self-reported ratings of the virtual nurse | patients reported high levels of satisfaction with the VN, thought the information was useful and helpful and 94% indicated the system was easy to use; 74% would prefer  receiving discharge instructions from the virtual nurse over their doctors or nurses |
| Brom et al. 2020 [10] | retrospective observational | 2.165;  242 readmissions (11.2%); mean age 63.6 | hospital | routine data from the health system’s data store | identify patients at risk for readmissions by applying a machine-learning technique, Classification and Regression Tree | all-cause readmission within 30 days of an indexed hospitalization to a medical service | highest risk for readmission among patients who visited the emergency department, had 9 or more comorbidities, were insured through Medicaid, and were 65 years of age and older |
| Carros et al. 2020 [11]* | case study, mixed methods: observations, interviews | 6 residents, 4 caregivers, 1 care home manger from a 119 resident facility | SLTC | not specified | better understand the real-world potential of robot-based assistance | expectations, attitudes, feelings, exercise patterns of residents and caregivers, use practices, performance and usability of the system | humanoid robots can work in a care home but a moderating person, that is in control of the robot, is needed |
| Chen et al. 2007 [12] | observational | 15 residents, 4 nurses, undefined number of nursing assistants of a dementia unit of a nonprofit community nursing home | sLTC | camera audio/image data | to develop a monitoring system and an automatic elopement detection algorithm to reduce the risks of un-witnessed elopements | elopement from ward | the HMM-based detector can detect most elopement-behaviours with reasonable false alarm rates |
| Cho et al. 2013 [13]* | controlled trial, before and after study | 1,214;  866 at risk-patients in intervention group, 348 control patients; 64 nurses | hospital | routine data from a clinical data repository | develop and assess the impact of a decision support intervention to predict Hospital-Acquired Pressure Ulcers (HAPU) | prevalence of HAPU length of stay  user adoption rate and attitudes | intervention group: HAPU prevalence rate fell from 21% to 4.0%, ICU LOS shortened from7.6 to 5.2 days. After adjustment for primary diagnoses and illness severity, the intervention group was significantly less likely than the baseline group to develop HAPU (OR = 0.1) and had a shorter ICU LOS (OR = 0.67) |
| Davoudi et al. 2019 [14] | observational | 22;  critically ill patients with (n=17) and without (n=8) ICU delirium | hospital | sensor data, video data | determine whether the Intelligent ICU system can be used to characterize the difference between patients’ functional status, pain and environmental exposure and test the feasibility of pervasive monitoring of ICU patients | ICU Delirium (CAM-ICU) | facial expressions, functional status entailing extremity movement and postures, and environmental factors including the visitation frequency, light and sound pressure levels at night were significantly different between the delirious and non-delirious patients |
| Evans et al. 2014 [15]* | observational longitudinal | 6289;  patients admitted to a 33-bed medical and oncology floor and a 33-bed non-intensive ICU surgical trauma floor | hospital | physiological sensor data and Electronic Medical Record data | develop and evaluate an automated case detection and response triggering system to monitor patients and identify early signs of physiologic deterioration | physiologic deterioration, Nurse LOC documentation, GCS, Confusion Assessment Method; CAM, RASS) | nurses reported the positive predictive value of alerts was 91-100%. During the intervention year, unit A patients had a significant increase in length of stay, more transfers to ICU, and significantly more medical emergency team calls, and significantly fewer died compared to the pre-intervention year. No significant differences were found on unit B. |
| Hirdes et al. 2008 [16] | observational | 4863 clients  assessed by 14 Community Care Access  Centres | aLTC | RAI-HC assessment data | develop and validate a methodology for prioritizing access to community and facility-based services | presence of signs of caregiver distress, rating oneself or being rated by others as  being better off elsewhere and nursing home admissions within the next  quarter after the RAI-HC assessment | the developed algorithm was a strong predictor of all three outcomes and provides a psychometrically sound decision-support tool that may be used to inform choices related to allocation of home care resources and prioritization of clients needing community or facility-based services |
| Marschollek et al. 2008 [17] | observational | 110 geriatric in-patients with varying degrees of fall risk | hospital | sensor data | assess the fall risk  by using parameters computed  using spectral analysis on data from a triaxial accelerometer  worn at the waist | low or high fall risk determined by TUG, STRATIFY score, Barthel index subscore mobility | TUG-model: high overall prediction accuracy and a high test sensitivity, but very low specificity is very low  STRATIFY-model: lower accuracy, albeit a fair compromise between sensitivity  (78.5%) and specificity (46.7%). The model  Barthel mobility subscore-model: overall predictive accuracy of 70.9% with the highest Kappa value among the models (0.40). |
| Matsuyama et al. 2009 [18]* | observational field experiment | 3 elderly people, 10 caregivers, unclear number of side participants | daycare | camera sensor data | develop a communication robot for activation that participates in group communication | ferquency of smiles and answers in response to the robot system | Frequency of panelists’ answers and frequency of smile were observed as almost the same in two conditions, the utterance variation of questions got a huge response |
| Mervin et al. 2018 [19]* | economic evaluation, nested within a cluster RCT | 415;  people with dementia ≥60 in 28 nursing homes | sLTC | not specified | examine the within-trial costs and cost-effectiveness of using PARO, compared with a plush toy and usual care, for reducing agitation and medication use | incremental cost per CMAI-SF point averted from a provider’s perspective; within-trial costs | within-trial costs: PARO group $50.47 more expensive per resident compared with usual care, plush toy group $37.26 more expensive than usual care, no statistically significant between-group differences in agitation levels after the 10-week intervention, point estimates of the incremental cost-effectiveness ratios were $13.01 for PARO and $12.85 for plush toy per CMAI-SF point averted relative to usual care |
| Papageorgiou et al. 2017 [20] | real-life use cases: 1. walking assistive device, 2. bathing assistance | unclear; patients with moderate to mild impairment at a hospital geriatric center | hospital | sensor data | explore new aspects of assistive living via intelligent assistive robotic systems involving human robot interaction in a natural interface to build assistive robotic systems, in order to increase the independence and safety of these procedures | technological functionality | initial performance assessment of the HMM-based methodology presented; the second use case used healthy subjects and therefore is not of relevance for this sample |
| Tang et al. 2019 [21]* | Observational, case-study | Not specifically stated, study conducte din a 200-resident facility | sLTC | EHR data, nursing care records, web healthcare information | develop a cloud-based nursing care planning system to facilitate decision making of admission staff | efficiency of nursing care plan formulation: response in handling new applications, revision of care plans, adoption of traditional healthcare services, complaint rate | Time in formulating nursing care plan reduced from 7 days to 4 days, time waiting for supporting documents  reduced from 24 hours to 6 hours, time in searching for healthcare information reduced from 90 minutes to 20 minutes |
| Tapia et al. 2019 [22]* | observational | 50 patients, 12 nurses | sLTC | sensor data | enhance the assistance and health care for Alzheimer patients | time spent on indirect nursing tasks (minutes), Number of nurses working simultaneously, detected accessed to restricted zones | average number of minutes spent by all nurses on monitoring  patients reduced from more than 150 daily minutes (before implementation) to approximately 90 daily minutes (implementation); number of accesses to restricted zones detected almost twice as often after implementation |
| Tawfik et al. 2020 [23] | observational | 276,054 infants cared for in 99 neonatal intensive care units | hospital | nurse staffing and organizational data, hospital routine data | develop a nurse staffing prediction model and evaluate deviation from predicted nurse staffing | nursing hours per patient-day,, health care-associated infections (HAIs), length of stay, mortality | prediction model explained 35 percent of the nurse staffing variation, higher-than-predicted nurse staffing was associated with decreased risk-adjusted odds of HAIs (OR: 0.79, 95%  CI: 0.63-0.98), but not with length of stay or mortality |
| Usach et al. 2017 [24] | unclear | not stated | sLTC | sensor data | develop an open source AAL system that aims to enhance quality of life of elderly people nursing houses | unclear, QoL | short description of field study in residences without giving detailed results |
| Viswanathan et al. 2012 [25]* | case study, single subject research design (SSRD) | 6; residents with mild-to-moderate cognitive impairment | sLTC | observations of user behavior, survey data | test the efficacy of a Navigation and Obstacle Avoidance Help system | frontal collisions, collision avoidance ability, compliance with promts | mean collisions are lowered for all participants, with large differences between participants in terms of collision avoidance ability |
| Wai et al. 2010 [26] | experimental, controlled trial | not reported | sLTC | sensor data | to measure the benefit gained from implementing a distributed sensor network system for managing incontinence among resdients with dementia | not specified | The system showed an average of 75% sensitivity and  96% specificity in detecting soiled diapers with actual diaper change, a 75% sensitivity indicates the effects of non-functional,  operational and usability issues |
| Xiong et al. 2019 [27]* | experimental, controlled trial | 55 residents with dementia | sLTC | image data | examine the use of a scalable AI-camera monitoring system to detect falls, videotape falls, and notify care staff to perform video review of the incident | number of EMT and ED visits | reduction in unnecessary EMT and ED visits by providing better understanding of unwitnessed falls. The AI-enabled camera fall-detection system coupled with staff review of fall videos led to more accurate identification of serious falls and incidents  compared with less serious falls |
| Yamamoto et al. 2020 [28]* | observational, comparative | 203;  Nursing students | education facility | image data | test a quantitative evaluation method of handwashing skills based on deep learning | handwashing skills (percentage of palm of the hand unwashed as detected by analysis of ultraviolet images) | experienced hand washers demonstrated almost the same skills as those of beginners |
| Ye et al. 2020 [29] | longitudinal cohort study | 265225; ≥65 years, visiting 35 hospitals, 34 federally qualified health centers over 2 years | hospital | electronic health record data | construct and validate an electronic health record-based fall risk predictive tool | fall risk predicted by a one-year fall prediction model | 50 % of the identified high-risk true positives were confirmed to fall during the first 94 days of next year (model attained a validated C-statistic of 0.807), 58.01 % and 54.93 % of falls that happened within the first 30 and 30–60 days of next year were also captured, XGBoost algorithm captured 157 predictors into the final predictive model, cognitive disorders, abnormalities of gait and balance, Parkinson’s disease, fall history and osteoporosis identified as the top-5 strongest predictors of future fall event |
| Zhang 2007 [30] | observational | 16; critically ill infants to adolescents | hospital | sensor data | examine the feasibility of developing patient-specific alarm algorithms in real time at the bedside and evaluate the potential of these algorithms in helping improve patient monitoring | patient-specific alarm algorithms for critical care | system was capable of training and evaluating patient-specific algorithms in a consistent manner in real time at the bedside, neural networks achieved a sensitivity of 0.96, a specificity of 0.99, a positive predictive value of 0.79, and an accuracy of 0.99 (0.84, 0.98, 0.72, and 0.98 respectively for the classification trees) |
| Zhou & Zhao 2006 [31] | observational | not stated; alzheimer patients and their caregivers | aLTC | interview/ questionnaire data | predict incidence and identify risk factors of psychological distress in AD patients using artificial neural networks, machine learning models, linear regression and decision tree models | psychological distress in AD patients | Among all models, the artificial neural networks with 8 hidden neurons achieved the highest predictive accuracy of 81.92%. In the five machine learning models, the ADTree algorithm made the highest Predictive Accuracy of 77.94%. As for risk factor analysis, the Linear Regression and Decision Tree models reported similar sets of variables that affect the psychological distress of AD patients. Three variables were reported by Linear Regression to be in negative correlation with psychological distress: the use of professional care service, caregiver consuming cigarette, and caregiver consuming alcohol |
| Yang et al. 2019 [32] | prospective, observational | 40 adults aged 65 to 93 | community | sensor data,  RAI-HC assessment data | investigate similarities and differences in physical activity, heart rate, and night sleep in a sample of community-dwelling older adults with varying fall histories using a smart wrist-worn device;  create and evaluate fall risk classification models based on wearable data, the RAI-HC, and (the combination of wearable and RAI-HC data | fall risk classification model | Random Forrest algorithm achieved an accuracy of 83.8% and scored higher accuracy than RAI-HC or sensor data alone. RAI-HC outperformed wearable data in fall risk classification while the best performance was achieved with the combination of the two data sets. |
| Zampieri et al. 2019 [33]* | retrospective, observational | 129680;  ≥16, admitted to 93 medical-surgical ICUs | hospital | primary patient data collection;  cross-sectional survey data from ICU director/chief nurse | study whether ICU staffing features are associated with improved hospital mortality, ICU LOS and duration of mechanical ventilation (MV) using cluster analysis directed by machine learning | in-hospital mortality ICU LOS  durations of MV (staffing variables: average bed to nurse, physiotherapist and physician ratios, presence of 24/7 board-certified intensivists and dedicated pharmacists in the ICU, nurse and physiotherapist autonomy scores) | distinguishing features of three clusters identified: presence of board-certified intensivists in the ICU 24/7 (cluster 3), dedicated pharmacists (clusters 2 and 3), extent of nurse autonomy increased from Clusters 1 to 3), patients in Cluster 3 exhibited the best outcomes, with lower adjusted hospital mortality (OR= 0.92), shorter ICU LOS [subhazard ratio (SHR) for patients surviving to ICU discharge 1.24 and shorter durations of MV [SHR for undergoing extubation 1.61] |
| Zhu et al. 2007a [34] | Retrospective, comparative | 24724;  home-care clients | aLTC | routine data, interRAI-HC | explore the potential to use an automatic, data-driven, machine-learning algorithm in clinical decision making by comparing the performance of a KNN algorithm and a Clinical Assessment Protocol (ADLAP) to predict rehabilitation potential | rehabilitation potential (functional improvement or remaining at home over a follow-up period  of approximately 1 year) | KNN algorithm had a lower false positive rate in all but one of the eight regions in the sample, and lower false negative rates in all regions. Compared using likelihood ratio statistics, KNN was uniformly more informative than the ADLCAP |
| Zhu et al. 2007b [35] | secondary analysis | 24724;  home-care clients | aLTC | routine data, interRAI-HC | investigate the potential of SVM and KNN algorithms to guide rehabilitation planning for home care clients | rehabilitation potential (improvement in ADL functioning or discharge home) | KNN and SVM algorithms achieved similar substantially improved performance over the ADLCAP, although false positive and false negative rates were still fairly high. Results are used to suggest potential revisions to the ADLCAP. |
| Legend: *)Categorized as reporting clinical or organizational effects. ACFI=Aged Care Funding Instrument, AD=Alzheimer’s Disease, ADLCAP =Activities of Daily Living Clinical Assessment Protocol, ALS=Amyotrophic lateral sclerosis, aLTC=ambulatory Long-term Care, CAM-ICU=Confusion Assessment Method for Intensive Care Unit, CMAI-SF=Cohen-Mansfield Agitation Inventory-Short Form, eFI=electronic Frailty Index, GCS=Glasgow Coma Score, HAPI= Hospital-Aquired Pressure Injury, HAPU=Hospital-Aquired Pressure Ulcers, HMM=Hidden Markov Model, KNN=K-Nearest-Neighbor, LOC=Level of Consciousness, LOS=Length of Stay, NLP=Natural Language Processing, PABAK=Prevalence-Adjusted and Bias-Adjusted Kappa, OR=Odds Ratio, RAI-HC=Resident Assessment Instrument Home Care, RASS=Richmond Agitation Sedation Scale, sLTC=stationary Long-term Care, SVM=Support Vector Machine, QoL=Quality of Life. | | | | | | | |

**References**

1. Abdullah R, Fakieh B. Health Care Employees' Perceptions of the Use of Artificial Intelligence Applications: Survey Study. Journal of medical Internet research. 2020 May 14;22(5):e17620. DOI:10.2196/17620. PMID:32406857

2. Ala-Kitula A, Talvitie-Lamberg K, Tyrväinen P, Silvennoinen M, editors. Developing Solutions for Healthcare — Deploying Artificial Intelligence to an Evolving Target. International Conference on Computational Science and Computational Intelligence; 2017; Las Vegas, USA. DOI:10.1109/CSCI.2017.285

3. Alderden J, Pepper GA, Wilson A, Whitney JD, Richardson S, Butcher R, et al. Predicting Pressure Injury in Critical Care Patients: A Machine-Learning Model. American journal of critical care: an official publication, American Association of Critical-Care Nurses. 2018 Nov;27(6):461-8. PMID:30385537

4. Alwan M. Passive in-home health and wellness monitoring: overview, value and examples. Conference proceedings: Annual International Conference of the IEEE Engineering in Medicine and Biology Society IEEE Engineering in Medicine and Biology Society Annual Conference. 2009 2009;2009:4307-10. DOI:10.1109/IEMBS.2009.5333799. PMID:19964350

5. Amato F, Bianchi S, Comai S, Crovari P, Pasquarelli MGG, Imtiaz A, et al., editors. CLONE: a Promising System for the Remote Monitoring of Alzheimer’s Patients: An Experimentation with a Wearable Device in a Village for Alzheimer’s Care. Goodtechs '18: Proceedings of the 4th EAI International Conference on Smart Objects and Technologies for Social Good; 2018 2018; Bologna, Italy: Association for Computing Machinery. DOI:10.1145/3284869.3284906

6. Ambagtsheer RC, Shafiabady N, Dent E, Seiboth C, Beilby J. The application of artificial intelligence (AI) techniques to identify frailty within a residential aged care administrative data set. International journal of medical informatics. 2020 Apr.13,6:104094. DOI:10.1016/j.ijmedinf.2020.104094. PMID:32058264

7. Antoniadi AM, Galvin M, Heverin M, Hardiman O, Mooney C. Prediction of caregiver burden in amyotrophic lateral sclerosis: a machine learning approach using random forests applied to a cohort study. BMJ open. 2020 Feb-28;10(2):e033109. PMID:32114464

8. Bajo J, Corchardo JM, Rodriguez S. GR-MAS: Multi-Agent System for Geriatric Residences. In: Ghallab M, Spyropoulos CD, Fakotakis N, Avouris N, editors. ECAI 18th European Conference on Artificial Intelligence; Patras, Greece 2008. ISBN:978-1-5-58603-891-5

9. Bickmore TW, Pfeifer LM, Jack BW. Taking the time to care. Empowering low health literacy hospital patients with virtual nurse agents. Proceedings of the 27th international conference on Human factors in computing systems - CHI 09; 2009; Boston, MA, USA: Association for Computing Machinery; 2009. DOI:10.1145/1518701.1518891

10. Brom H, Brooks Carthon JM, Ikeaba U, Chittams J. Leveraging Electronic Health Records and Machine Learning to Tailor Nursing Care for Patients at High Risk for Readmissions. Journal of nursing care quality. 2020 Jan-Mar;35(1):27-33. PMID:31136529

11. Carros F, Meurer J, Löffler D, Unbehaun D, Matthies S, Koch I, et al. Exploring Human-Robot Interaction with the Elderly. Proceedings of the 2020 CHI Conference on Human Factors in Computing Systems; 2020; Honolulu, HI, USA: Association for Computing Machinery; 2020. p. 1-12. DOI:10.1145/3313831.3376402

12. Chen D, Bharucha AJ, Wactlar HD, editors. Intelligent Video Monitoring to Improve Safety of Older Persons. 2007 Annu Int Conf IEEE Eng Med Biol Soc. 007;2007:3814-7. DOI:10.1109/IEMBS.2007.4353163. PMID: 18002829

13. Cho I, Park I, Kim E, Lee E, Bates DW. Using EHR data to predict hospital-acquired pressure ulcers: a prospective study of a Bayesian Network model. International journal of medical informatics. 2013 Nov;82(11):1059-67. PMID:23891086

14. Davoudi A, Malhotra KR, Shickel B, Siegel S, Williams S, Ruppert M, et al. Intelligent ICU for Autonomous Patient Monitoring Using Pervasive Sensing and Deep Learning. Scientific reports. 2019 May-29;9(1):8020. PMID:31142754

15. Evans RS, Kuttler KG, Simpson KJ, Howe S, Crossno PF, Johnson KV, et al. Automated detection of physiologic deterioration in hospitalized patients. Journal of the American Medical Informatics Association: JAMIA. 2015 Mar;22(2):350-60. PMID:25164256

16. Hirdes JP, Poss JW, Curtin-Telegdi N. The Method for Assigning Priority Levels (MAPLe): a new decision-support system for allocating home care resources. BMC medicine. 2008 Mar 26;6:9. DOI:10.1186/1741-7015-6-9. PMID:18366782

17. Marschollek M, Wolf K, Gietzelt M, Nemitz G, Schwabedissen HMz, Haux R, editors. Assessing elderly persons' fall risk using spectral analysis on accelerometric data - a clinical evaluation study. 2008 20-25-Aug. DOI:10.1109/IEMBS.2008.4650008

18. Matsuyama Y, Taniyama H, Fujie S, Kobayashi T. System design of group communication activator: an entertainment task for elderly care. 2009; La Jolla, California, USA: Association for Computing Machinery; 2009. p. 243–4. DOI:10.1145/1514095.1514157

19. Mervin MC, Moyle W, Jones C, Murfield J, Draper B, Beattie E, et al. The Cost-Effectiveness of Using PARO, a Therapeutic Robotic Seal, to Reduce Agitation and Medication Use in Dementia: Findings from a Cluster-Randomized Controlled Trial. Journal of the American Medical Directors Association. 2018 Jul;19(7):619-22 e1. DOI:10.1016/j.jamda.2017.10.008. PMID:29325922

20. Papageorgiou XS, Chalvatzaki G, Dometios A, Tzafestas CS, Maragos P, editors. Intelligent Assistive Robotic Systems for the elderly: Two real-life use cases. 2017 2017: ACM. DOI:10.1145/3056540.3076184

21. Tang V, Siu PKY, Choy KL, Lam HY, Ho GTS, Lee CKM, et al. An adaptive clinical decision support system for serving the elderly with chronic diseases in healthcare industry. Expert Systems. 2019 2019;36(2). DOI:10.1111/exsy.12369

22. Tapia DI, Rodr\iguez S, Corchado JM. A Distributed Ambient Intelligence Based Multi-Agent System for Alzheimer Health Care. Springer; 2010. p. 181-99. DOI:10.4018/jaci.2009010102

23. Tawfik DS, Profit J, Lake ET, Liu JB, ers LM, Phibbs CS. Development and use of an adjusted nurse staffing metric in the neonatal intensive care unit. Health services research. 2020 Apr;55(2):190-200. PMID:31869865

24. Gonzalez-Usach R, Collado V, Esteve M, Palau CE, Fortino G, Zhou M, et al., editors. AAL open source system for the monitoring and intelligent control of nursing homes. 2017: IEEE. DOI:10.1109/ICNSC.2017.8000072

25. Viswanathan P, Little JJ, Mackworth AK, Mihailidis A, editors. An Intelligent Powered Wheelchair for Users with Dementia: Case Studies with NOAH (Navigation and Obstacle Avoidance Help). 2012: AAAI.

26. Wai AAP, Fook FS, Jayach, ran M, Biswas J, Lee J-E, et al., editors. Implementation of Context-Aware Distributed Sensor Network System for Managing Incontinence Among Patients with Dementia. 2010 2010: IEEE. DOI:10.1109/BSN.2010.15

27. Xiong GL, Bayen E, Nickels S, Subramaniam R, Agrawal P, Jacquemot J, et al. Real-time video detection of falls in dementia care facility and reduced emergency care. The American journal of managed care. 2019 Jul;25(7):314-5. PMID:31318502

28. Yamamoto K, Yoshii M, Kinoshita F, Touyama H, editors. Classification vs Regression by CNN for Handwashing Skills Evaluations in Nursing Education. 2020 19-21-Feb. DOI:10.1109/ICAIIC48513.2020.9064974

29. Ye C, Li J, Hao S, Liu M, Jin H, Zheng L, et al. Identification of elders at higher risk for fall with statewide electronic health records and a machine learning algorithm. International journal of medical informatics. 2020 2020;137. DOI:10.1016/j.ijmedinf.2020.104105. PMID:142476870

30. Zhang Y. Real-time development of patient-specific alarm algorithms for critical care. Conference proceedings: Annual International Conference of the IEEE Engineering in Medicine and Biology Society IEEE Engineering in Medicine and Biology Society Annual Conference. 2007 2007;2007:4351-4. DOI:10.1109/IEMBS.2007.4353300. PMID:18002966

31. Zhou X, Xu J, Zhao Y. Machine learning methods for anticipating the psychological distress in patients with Alzheimer's disease. Australasian physical & engineering sciences in medicine. 2006 Dec;29(4):303-9. DOI:10.1007/BF03178395. PMID:17260584

32. Yang Y, Hirdes JP, Dubin JA, Lee J. Fall Risk Classification in Community-Dwelling Older Adults Using a Smart Wrist-Worn Device and the Resident Assessment Instrument-Home Care: Prospective Observational Study. JMIR aging. 2019 Jun 7;2(1):e12153. DOI:10.2196/12153. PMID:31518278

33. Zampieri FG, Salluh JIF, Azevedo LCP, Kahn JM, Damiani LP, Borges LP, et al. ICU staffing feature phenotypes and their relationship with patients' outcomes: an unsupervised machine learning analysis. Intensive care medicine. 2019 Nov;45(11):1599-607. PMID:31595349

34. Zhu M, Chen W, Hirdes JP, Stolee P. The K-nearest neighbor algorithm predicted rehabilitation potential better than current Clinical Assessment Protocol. Journal of clinical epidemiology. 2007 Oct;60(10):1015-21. PMID:17884595

35. Zhu M, Zhang Z, Hirdes JP, Stolee P. Using machine learning algorithms to guide rehabilitation planning for home care clients. BMC medical informatics and decision making. 2007 Dec-20;7:41. PMID:18096079
